# Aethiopinolones A–E, New Pregnenolone Type Steroids from the East African Basidiomycete *Fomitiporia aethiopica*

**DOI:** 10.3390/molecules23020369

**Published:** 2018-02-09

**Authors:** Clara Chepkirui, Winnie C. Sum, Tian Cheng, Josphat C. Matasyoh, Cony Decock, Marc Stadler

**Affiliations:** 1 Department of Microbial Drugs, Helmholtz Centre for Infection Research and German Centre for Infection Research (DZIF), Partner Site Hannover/Braunschweig, Inhoffenstrasse 7, 38124 Braunschweig, Germany; clara.chepkirui@helmholtz-hzi.de (C.C.); Tian.Cheng@helmholtz-hzi.de (T.C.); 2 Department of Biochemistry, Egerton University, P.O. BOX 536, Njoro 20115, Kenya; winsumbt@gmail.com; 3 Department of Chemistry, Egerton University, P.O. BOX 536, Njoro 20115, Kenya; josphat2001@yahoo.com; 4 Mycothéque de l’ Universite Catholique de Louvain (BCCM/MUCL), Place Croix du Sud 3, B-1348 Louvain-la-Neuve, Belgium; Cony.Decock@uclouvain.be

**Keywords:** cytotoxicity, fungi, Hymenochaetaceae, triterpenes

## Abstract

A mycelial culture of the Kenyan basidiomycete *Fomitiporia aethiopica* was fermented on rice and the cultures were extracted with methanol. Subsequent HPLC profiling and preparative chromatography of its crude extract led to the isolation of five previously undescribed pregnenolone type triterpenes **1**–**5**, for which we propose the trivial name aethiopinolones A–E. The chemical structures of the aethiopinolones were determined by extensive 1D- and 2D-NMR, and HRMS data analysis. The compounds exhibited moderate cytotoxic effects against various human cancer cell lines, but they were found devoid of significant nematicidal and antimicrobial activities.

## 1. Introduction

The fungal kingdom includes many species that produce various classes of structurally unique and biologically active metabolites [1,2]. Of interest to us are the largely neglected basidiomycetes and more so, those available from rich untapped sources like the African tropics. During the course of our studies on Kenya’s tropical basidiomycetes we have encountered various interesting organisms that yielded new biologically active metabolites such as laetiporins, calocerins, 9-oxostrobilurins and laxitextines [3,4,5]. Another specimen collected in Kenya was identified as *Fomitiporia aethiopica*, a species that had been first reported from the Ethiopian highlands [6]. The mycelial culture showed an interesting secondary metabolite profile when studied by HPLC-MS. Although the taxonomy of the genus *Fomitiporia* in Africa has been reported, the secondary metabolites of these genus have so far not been studied extensively, even though one of its species is involved in the esca disease syndrome of grapevine [6,7,8]. The present paper is dedicated to the first investigation of the secondary metabolite production in mycelial cultures of *Fomitiporia aethiopica*.

## 2. Results and Discussion

### 2.1. Structure Elucidation

Solid phase fermentation on rice of the strain *Fomitiporia aethiopica* was carried out as described in the Materials and Methods section. In the antimicrobial assay the crude extracts initially showed activity against *Bacillus subtilis* but the activity was later attributed to fatty-acid like components of the extracts. However, we found some interesting peaks upon analysis of the HPLC-MS data. A subsequent search in the Dictionary of Natural Products database suggested the presence of hitherto undescribed metabolites [9]. Scale-up of fermentation and subsequent preparative chromatography yielded five new triterpenes **1**–**5**, for which we propose the trivial names aethiopinolones A–E.

Aethiopinolone A (**1**) was isolated as yellow oil with the molecular formula C_21_H_30_O_5_ and seven degrees of unsaturation deduced from the HRMS data. The ^13^C-NMR spectroscopic data of **1** revealed the presence of 21 carbon signals (Table 1). From the DEPT NMR data three methyl groups, six methylene groups, six methane groups and six quaternary carbons were identified. In the ^1^H-NMR spectrum, three methyl singlets resonating at δ 0.58 (H_3_-18), δ 0.93 (H_3_-19) and δ 2.16 (H_3_-21) were recorded. Further, peaks at δ 2.71 (H-17), δ 3.50 (H-3) and 4.74 (H-16) attributed to oxygenated methine groups were observed in the ^1^H-NMR.

**Figure 1:**
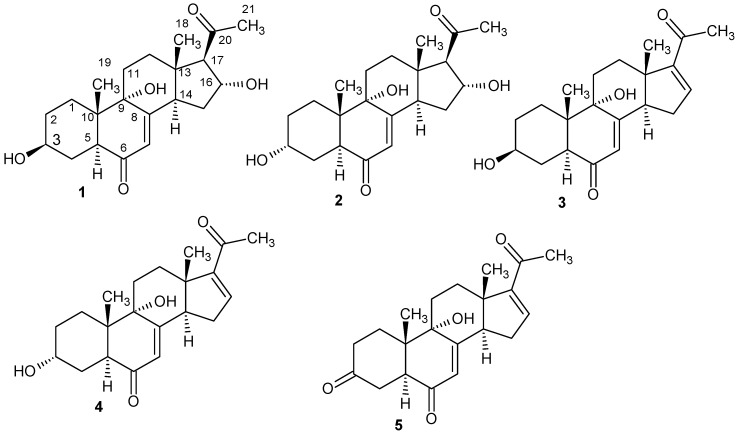
Chemical structures of **1**–**5**.

HMBC correlations of H_3_-18 to C-12/C-13/C-14/C-17, H_3_-19 to C-1/C-5/C-9/C-10, H-5 to C-3/C-4/C-6/C-9/C-10/C-19, H-14 to C-7/C-8/C-9/C-13/C-15/C-18 and H_3_-21 to C-17/C-20 suggested a pregnenolone type of steroids (Figure 1). COSY correlations of H_2_-2 to H_2_-1/H-3, H_2_-4 to H-3/H-5, H_2_-11 to H_2_-12, H-14 to H_2_-15 and H-16 to H_2_-15/H-17 further supported these HMBC correlations. Furthermore, cross peaks observed between H-7 and C-5/C-9/C-14 in the HMBC spectrum and long range COSY correlation to H-14 with a coupling constant of 1.9 Hz helped to confirm the position of the double bond.

**Figure 2:**
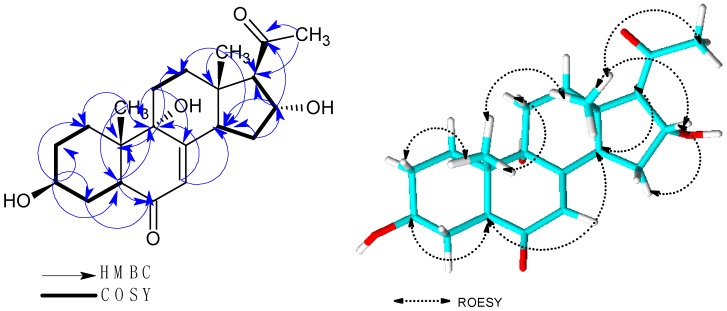
HMBC, COSY and ROSY correlations of **1**.

A network of ROESY correlations were observed between H_3_-18 to H_3_-19/H_2_-12β/H-15β/H-16/H_3_-21 and H_3_-19 to H_3_-18/ H_2_-1β/H_2_-11β, suggesting that these protons are on the same side of the plane (Figure 2). On the other hand, cross peaks in the ROESY spectra were observed between H-3 to H_2_-1α/H_2_-4α/H_2_-2α/H-5, H-14 to H_2_-15α/H_2_-12α/H-17 and H-17 to H_2_-15α/H-14. For H-3, an α orientation was established based on the coupling constant of 4.52 Hz and 11.3 Hz together with the ROESY correlations of H-3 to H-5/H_2_-1α/ H_2_-4α/H_2_-2α. Conversely, H-16 was β oriented as indicated by the small coupling constant of 3.0 Hz and the cross peaks observed between H-16 and H_3_-18/H_2_-15β in the ROESY spectrum. The absolute configuration of **1** was finally determined using Mosher’s method [10]. The difference in chemical shifts Δδ^SR^ = (Δ*s* − δ*R*) for protons neighboring C-3 gave positive values +0.079, +0.041 and +0.015 for H_2_-4α, H_2_-4β and H-5 respectively. On the contrary, negative values of Δδ^SR^ i.e., −0.078 (H_2_-α), −0.110 (H_2_-2β), −0.016 (H-2-1α), −0.061 (H_2_-1β) and −0.011 (H_3_-19 were obtained. Therefore the absolute configuration of the C-3 stereocenter can be assigned as *S* (Supplementary Information, Table S1). Using C-3 as the reference the other stereo centers were assigned as 5*S*, 9*S*, 10*S*, 13*S*, 14*R*, 16*R* and 17*R*.

Aethiopinolone B (**2**) was obtained as a white solid. The molecular formula C_21_H_30_O_5_ and seven degrees of unsaturation were deduced from the HR mass spectrum. The 1D and 2D-NMR data of **2** suggested that it possesses the same planar structure as **1**, the difference being the stereochemistry at C-3. The ROESY correlation of H-3 to H_3_-19 and the OH-3 (δ 4.26) to H-5 pointed to H-3 having β orientation. In the ^1^H-NMR spectrum of compound **1** H-3 resonated as a triple triplet while H-3 of compound **2** resonated as multiplet. Furthermore, C-3 was slightly shielded resonating at δ 63.1 as compared to C-3 of **1**, which occurred at δ 70.3. Consequently the stereochemistry at C-3 was assigned as *R*.

Aethiopinolone C (**3**) with a molecular formula C_21_H_28_O_4_ and eight degrees of unsaturation deduced from HR mass spectrum was further isolated as yellow oil. Analysis of the ^1^H-NMR revealed the absence of the methine proton at δ 2.71 (H-17) and the oxygenated methine proton δ 4.74 (H-16) which were observed in the ^1^H-NMR of **1**. Instead an olefinic doublet of doublet resonating at δ 6.91 (H-16) was recorded. In the ^13^C spectrum new signals at δ 144.3 and δ 155.1 were identified (Table 2). H-16 showed HMBC correlations to C-13/C-14/C-20 and COSY correlations to H_2_-15 implying that **3** has similar planar structure as **1** with the difference being the double bond between C-16/C-17. Further, similar ROESY correlations patterns in compounds **1** and **3** were recorded. Derivatization of **3** with both *S* and *R*- MPA chloride gave similar results as **1** for protons neighboring C-3 hence the absolute stereochemistry was assigned as 3*S*, 5*S*, 9*S*, 10*S*, 13*S* and 14*R* (Table S2, SI).

Aethiopinolone D (**4**) was isolated as yellow oil with molecular formula C_21_H_28_O_4_ and eight degrees of unsaturation established from the HRMS data. The 1D and 2D data of **4** indicated that **4** was analogous to **3** with the difference being the stereochemistry at C-3. The same ROESY correlations of H-3 to H_3_-19, H-3 multiplicity in the ^1^NMR spectrum and the shielding effect reported in ^13^C-NMR data of compound **2** were also observed in the ^13^C-NMR of **4**, evincing the same stereochemistry at C-3 for the two molecules. Esterification of compound **4** with both *S* and *R*- MPA chloride, and subsequent determination of Δδ^SR^ from the resulting esters gave negative values −0.096, −0.012 and −0.11 for H-5, H_2_-4β and H_2_-4α respectively. Positive values +0.005, +0.162, +0.028 for H-19, H_2_-2β and H_2_-2α respectively were obtained (Supplementary Information, Table S3). Therefore the absolute stereochemistry of compound **4** was assigned as 3*R*, 5*S*, 9*S*, 10*S*, 13*S* and 14*R*.

Aethiopinolone E (**5**) was obtained as yellow oil. The molecular formula C_21_H_26_O_4_ and nine degrees of unsaturation were deduced from the HRMS data. Analysis of the ^13^C and DEPT NMR data of **5** indicated that it was similar to **3** with the difference being a keto group at position 3. The oxygenated methine signal of compound **3** resonating at δ 70.3 was missing and instead a keto carbonyl group was observed at δ 210.1. HMBC correlations of diastereotopic protons H_2_-2α (δ 2.35), H_2_-2β (δ 2.26), H_2_-4α (δ 2.39), H_2_-4β (δ 2.51), to this carbon supported the assignment.

### 2.2. Biological Activities

Compounds **1**–**5** were tested for their cytotoxic effects against various mammalian cell lines (Table 3). **3**–**4** showed moderate activity against all the tested cell lines. All compounds apart from **2** showed significant activities against MCF-7 and A431 with IC_50_ in the range 16–20 µg/mL and 14–27 µg/mL respectively. Compound **1** generally showed the strongest activity against the tested cell lines with the highest effects against PC-3 (8 µg/mL). Compound **2** showed moderate activity only against L929 and KB3.1 cells with IC_50_ of 45 µg/mL and 39 µg/mL respectively. Aside from these cytotoxic activities, compounds **1**–**5** were found devoid of significant antimicrobial and nematicidal effects at concentrations ≤300 µg/mL and ≤100 µg/mL respectively.

Although steroids are rather common in the Basidiomycota, they have not been reported yet from the genus *Fomitiporia*. Studies on *Fomitiporia ellipsoidea* metabolites indicated that this fungus produced a large amount of common ergosterol and its derivatives but this species has since been moved to the genus *Phellinus* (currently valid name: *Phellinus ellipsoidea*) [11,12]. The close relationship between these two genera has seen the transfer of several other species previously assigned to the genus *Phellinus* to the genus *Fomitiporia*, examples being species like *F. erecta*, *F. hartigii*, *F. robusta*, *F. punctata*, *F. hippophaeicola* and *F. pseudopunctata* [13]. Styrylpyrones like the protein kinase C inhibitor, bihispidinyl and hypholomin B, which are common metabolites among the Hymenochaetales, have been reported to occur in some *Fomitiporia* species [14,15].

## 3. Materials and Methods

### 3.1. General Experimental Procedures 

Optical rotations were determined with a Perkin-Elmer (Überlingen, Germany) 241 spectrometer; UV spectra were recorded with a Shimadzu (Duisburg, Germany) UV-2450 UV-vis spectrophotometer. NMR spectra were recorded with a Bruker (Bremen, Germany) Ascend 700 spectrometer equipped with a 5 mm TXI cryoprobe (^1^H-700 MHz, ^13^C-175 MHz) and Bruker AV II-600 (^1^H-500 MHz, ^13^C-150 MHz) spectrometers. HR-ESI-MS mass spectra were recorded with a Bruker (Bremen, Germany) Agilent 1260 series HPLC-UV/Vis system (column 2.1 × 50 mm, 1.7 µm, C18 Acquity UPLC BEH (waters), solvent A: H_2_O + 0.1% formic acid; solvent B: AcCN + 0.1% formic acid, gradient: 5% B for 0.5 min increasing to 100% B in 19.5 min and then maintaining 100% B for 5 min, flow rate 0.6 mL/min^−1^, uv/vis detection 200–600 nm combined with ESI-TOF-MS (Maxis, Bruker) [scan range 100–2500 *m*/*z*, capillary voltage 4500 V, dry temperature 200 °C]. Chemicals and solvents were obtained from AppliChem GmbH (Darmstadt, Germany), Avantor Performance Materials (Arnhem, Netherlands), Carl Roth GmbH & Co. KG (Karlsruhe, Germany) and Merck KGaA (Darmstadt, Germany) in analytical and HPLC grade.

### 3.2. Fungal Material

The specimen MUCL 56047 was collected from Mount Elgon, located in the western part of Kenya (1°7′6″ N, 34°31′30″ E) by C. Decock in April 2016 (collection and isolation number KE/16-163). The dried herbarium specimen and culture are deposited at MUCL (Louvain-la-Neuve, Belgium) as MUCL 56047. The fungus was identified as *Fomitiporia aethiopica* by morphological studies and sequencing of the rDNA (5.8S gene region, the internal transcribed spacer ITS1 and ITS2). Genomic DNA Miniprep kit (Bio Basic Canada Inc., Markham, ON, Canada). A Precellys 24 homogenizer (Bertin Technologies, Saint-Quentin-en-Yvelines, France) was used for cell disruption at a speed of 6000 rpm for 2 × 40 s. The gene regions were amplified with primers ITS 1f and ITS 4. Details are given in the Supplementary Material.

### 3.3. Fermentation

The mycelial culture of MUCL 56047 was subjected to solid state fermentation in rice according to [16] with slight modifications. The rice medium was prepared by weighing 90 g of rice into 500 mL Erlenmeyer flasks containing in 90 mL of distilled water and autoclaved twice. A well-grown YMG agar plate of the mycelial culture was cut into small pieces using a 7 mm cork borer and five plugs inoculated into each of the 21 flasks containing sterile rice media. The cultures were incubated in a dark room at 23 °C for 28 days.

### 3.4. Extraction

The cultures were diced into smaller pieces with a spatulum and each of the 21 flasks was soaked in 150 mL of methanol overnight. Repeated extraction and filtration in an ultrasonic bath at 40° C for 30 min until an exhausted residue was yielded was carried out. The residue was discarded and the filtrate evaporated by means of a rotary evaporator. The resulting aqueous phase was suspended in equal amount of distilled water and extracted with equal amount of ethyl acetate four times. The aqueous phase was discarded and the organic phase filtered through anhydrous sodium sulphate. The resulting ethyl acetate extracts were evaporated to dryness by means of rotary evaporator to afford 800 mg of crude product.

### 3.5. Isolation and Physico-Chemical Characteristics of Compounds ***1**–**5***

The crude extract was fractionated using preparative reverse phase liquid chromatography (PLC 2020, Gilson, Middleton, MA, USA). A VP Nucleodur 100-5 C 18 ec column (250 × 40 mm, 7 μm: Macherey-Nagel, Schkeuditz, Germany) was used. Deionized water (Milli-Q, Millipore, Schwalbach, Germany) (solvent A) and acetonitrile (solvent B) were used as the mobile phase. The elution gradient used was 10–100% solvent B in 55 min and thereafter isocratic condition at 100% solvent B for 10 min. UV detection was carried out at 210, 254 and 350 nm. Seven fractions (F1–F7) were collected according to the observed peaks. Fraction F4 was purified by reverse phase LC (solvent A/solvent B), elution gradient 20–35% solvent B for 30 min, followed by a gradient shift from 35% to 100% in 3 min and finally isocratic condition at 100% solvent B for 5 min with a preparative (Kromasil, Mainz, Germany) 250 × 20 mm, 7 μL C-18 column as stationary phase to give compounds **1** (30 mg) and **3** (16 mg). Using the same column and elution gradient 25–40% solvent B for 35 min, fraction F6 was purified to afford 60 mg of compound **2**, as well as 12 mg of **4** and 8 mg of **5**.

*Aethiopinolone A* (**1**): Yellow oil; ${\left[\alpha \right]}_{D}^{25}$ −13° (*c* 0.001, MeOH); UV (MeOH) λ_max_(log ε) 236 (3.73); HREIMS *m*/*z* 363.2166 (calcd. for C_21_H_31_O_5_, 363.2171); ^1^H-NMR (acetone-*d*_6_, 700 MHz) and ^13^C-NMR (acetone-*d*_6_, 175 MHz) data: see Table 1.

*Aethiopinolone B* (**2**): White solid; ${\left[\alpha \right]}_{D}^{25}$ −33°, (*c* 0.001, MeOH); UV (MeOH) λ_max_(log ε) 240 (3.77); HREIMS *m*/*z* 363.2162 (calcd. for C_21_H_31_O_5_, 363.2171); ^1^H-NMR (DMSO, 500 MHz) and ^13^C-NMR (DMSO, 125 MHz) data: see Table 1.

*Aethiopinolone C* (**3**): Yellow oil; ${\left[\alpha \right]}_{D}^{25}$ +15°, (*c* 0.001, MeOH); UV (MeOH) λ_max_(log ε) 234 (3.45); HREIMS *m*/*z* 345.2060 (calcd. for C_21_H_29_O_4_, 345.2065); ^1^H-NMR (acetone-*d*_6_, 500 MHz) and ^13^C-NMR (acetone-*d*_6_, 125 MHz) data: see Table 2.

*Aethiopinolone D* (**4**): Yellow oil; ${\left[\alpha \right]}_{D}^{25}$ +19°, (*c* 0.001, MeOH); UV (MeOH) λ_max_(log ε) 234 (3.38); HREIMS *m*/*z* 345.2052 (calcd. for C_21_H_29_O_4_, 345.2065); ^1^H-NMR (acetone-*d*_6_, 700 MHz) and ^13^C-NMR (acetone-*d*_6_, 175 MHz) data: see Table 2.

*Aethiopinolone E* (**5**): Yellow oil; ${\left[\alpha \right]}_{D}^{25}$ +10°, (*c* 0.001, MeOH); UV (MeOH) λ_max_(log ε) 238 (3.61); HREIMS *m*/*z* 343.1901 (calcd. for C_21_H_27_O_4_, 343.1909); ^1^H-NMR (acetone-*d*_6_, 700 MHz) and ^13^C-NMR (acetone-*d*_6_, 175 MHz) data: see Table 2.

*Aethiopinolone A* (**1**) *3-O-(S)-MTPA ester*: ^1^H-NMR (chloroform-d, 700 MHz) δ 7.55 (2H, m, ArH), 7.43 (3H, m, ArH), 5.766 (1H, d, *J* = 2.2 Hz, H-7), 5.016 (1H, tt, *J* = 4.5, 11.8 Hz, H-3), 3.994 (1H, m, H-16), 3.590 (3H, s, OCH_3_), 3.040 (1H, m, H-14), 2.985 (1H, dd, *J* = 3.9, 12.3, H-5), 2.472 (1H, d, *J* = 5.8 Hz, H-17), 2.443 (1H, m, 15α), 2.388 (1H, m, H-4α), 2.309 (3H, s, H-21), 2.011 (1H, m, H-1α), 1.945 (1H, m, H-2α), 1.631 (1H, m, H-4β), 1.568 (1H, m, H-15α), 1.515 (1H, m, H-2β), 1.303 (1H, m, H-1β), 1.034 (3H, s, H-19), 0.896 (3H, s, H-18). EIMS *m*/*z* 579.27 (calcd. for C_31_H_38_F_3_O_7_, 579.2569)

*Aethiopinolone A* (**1**) *3-O-(R)-MTPA ester*: ^1^H-NMR (chloroform-d, 700 MHz) δ 7.55 (2H, m, ArH), 7.43 (3H, m, ArH), 5.757 (1H, d, *J* = 1.9 Hz, H-7), 5.012 (1H, tt, *J* = 4.7, 11.6 Hz, H-3), 3.994 (1H, m, H-16), 3.570 (3H, s, OCH_3_), 3.038 (1H, m, H-14), 2.970 (1H, dd, *J* = 3.9, 12.5, H-5), 2.473 (1H, d, *J* = 5.6 Hz, H-17), 2.446 (1H, m, 15α), 2.334 (1H, m, H-1α), 2.309 (1H, m, H-4α), 2.310 (3H, s, H-21), 2.024 (1H, m, H-2α), 1.625 (1H, m, H-2β), 1.569 (1H, m, H-15α), 1.590 (1H, m, H-4β), 1.364 (1H, m, H-1β), 1.045 (3H, s, H-19), 0.897 (3H, s, H-18). EIMS *m*/*z* 579.29 (calcd. for C_31_H_38_F_3_O_7_, 579.2569)

*Aethiopinolone C* (**3**) *S-MTPA ester*: ^1^H-NMR (pyridine-*d*_5_, 700 MHz) δ 7.40–7.48 (5H, m, ArH), 6.615 (1H, dd, *J* = 1.9, 3.2, H-16), 5.956 (1H, d, *J* = 2.2 Hz, H-7), 5.290 (1H, tt, *J* = 4.7, 11.6, H-3), 3.528 (1H, dd, *J* = 3.7, 12.3 Hz, H-5), 3.695 (3H, s, OCH3), 3.413 (1H, ddd, *J* = 1.9, 6.5, 11.6 Hz, H-14), 2.711 (1H, m, H-4α), 2.397 (2H, m, H-1), 2.2351 (1H, m, 15α), 2.238 (1H, m, H-15β), 2.251(3H, s, H-21), 1.983 (1H, m, H-2α), 1.926(1H, m, H-4β), 1.556 (1H, m, H-2β), 1.019 (3H, s, H-19), 0.948 (3H, s, H-18). EIMS *m*/*z* 561.28 (calcd. for C_31_H_36_F_3_O_6_, 561.2463)

*Aethiopinolone C* (**3**) *R-MTPA ester*: ^1^H-NMR (pyridine-*d*_5_, 700 MHz) δ 7.40–7.47 (5H, m, ArH), 6.612 (1H, dd, *J* = 1.7, 3.2, H-16), 5.944 (1H, d, *J* = 2.2 Hz, H-7), 5.287 (1H, tt, *J* = 4.5, 11.8, H-3), 3.506 (1H, dd, *J* = 3.9, 12.5 Hz, H-5), 3.852 (3H, s, OCH3), 3.399 (1H, ddd, *J* = 2.2, 6.0, 11.6 Hz, H-14), 2.669 (1H, m, H-4α), 2.418 (2H, m, H-1), 2.2350 (1H, m, 15α), 2.232 (1H, m, H-15β), 2.250(3H, s, H-21), 2.049 (1H, m, H-2α), 1.846(1H, m, H-4β), 1.685 (1H, m, H-2β), 1.027 (3H, s, H-19), 0.950 (3H, s, H-18). EIMS *m*/*z* 561.26 (calcd. for C_31_H_36_F_3_O_6_, 561.2463)

*Aethiopinolone D* (**4**) *S-MTPA ester*: ^1^H-NMR (pyridine-*d*_5_, 700 MHz) δ 7.40–7.47 (5H, m, ArH), 6.600 (1H, dd, *J* = 1.9, 3.4, H-16), 5.910 (1H, d, *J* = 2.2 Hz, H-7), 5.639 (1H, m, H-3), 3.619 (1H, dd, *J* = 4.1, 12.5 Hz, H-5), 3.850 (3H, s, OCH3), 3.367 (1H, ddd, *J* = 2.2, 6.0, 11.6 Hz, H-14), 2.657 (1H, 1β, H-1), 2.530 (1H, m, H-4α), 2.296 (1H, m, 15α), 2.222 (1H, m, H-15β), 2.078 (1H, m, H-2α), 2.015(3H, s, H-21), 1.999 (1H, m, H-4β), 1.741 (1H, m, H-2β), 1.361 (1H, m, 1α), 1.067 (3H, s, H-19), 0.971 (3H, s, H-18). EIMS *m*/*z* 561.30 (calcd. for C_31_H_36_F_3_O_6_, 561.2463)

*Aethiopinolone D* (**4**) *R-MTPA ester*: ^1^H-NMR (pyridine-*d*_5_, 700 MHz) δ 7.40–7.47 (5H, m, ArH), 6.600 (1H, dd, *J* = 1.7, 3.2, H-16), 5.945 (1H, d, *J* = 2.2 Hz, H-7), 5.640 (1H, m, H-3), 3.715 (1H, dd, *J* = 4.1, 12.2 Hz, H-5), 3.698 (3H, s, OCH3), 3.366 (1H, ddd, *J* = 1.94, 6.2, 11.8 Hz, H-14), 2.640 (1H, m, H-4α), 2.507 (1H, 1β, H-1), 2.300 (1H, m, 15α), 2.229 (1H, m, H-15β), 1.976 (1H, m, H-2α), 2.015(3H, s, H-21), 2.011 (1H, m, H-4β), 1.713 (1H, m, H-2β), 1.298 (1H, m, 1α), 1.062 (3H, s, H-19), 0.959 (3H, s, H-18). EIMS *m*/*z* 561.29 (calcd. for C_31_H_36_F_3_O_6_, 561.2463)

### 3.6. Preparation of the (R)- and (S)-MTPA Ester Derivatives 

Compound **1** (3 mg) were dissolved in pyridine (6 mL) and transferred into two vials (3 mL each). (*R*)-(−)-α-Methoxy-α-(trifluoromethyl)phenylacetyl chloride (5 μL) was added to one of the vials and (*S*)-(+)-α-methoxy-α-(trifluoromethyl)phenylacetyl chloride (5 μL) was added into the other vial, and stirred for 1 h. The products were purified by reverse phase LC (acetonitrile (B)/H_2_O (A), elution gradient 40–100% solvent B for 25 mins followed by isocratic condition at 100% solvent B for 5 min with a preparative (VP 250/10 NUCLEODUR 100-5 C18 ec, Macherey-Nagel, Schkeuditz, Germany) 250 × 10 mm, C-18 column as stationary phase and 6mL/min flow rate. ^1^H-NMR and ^1^H, ^1^H COSY of the samples were recorded after wards. Compound **3** and **4** (3 mg each) were dissolved in deuterated pyridine (6 mL) and transferred into two vials (3mL each). (*R*)-(−)-α-Methoxy-α-(trifluoromethyl)phenylacetyl chloride (5 μL) was added to one of the vials and (*S*)-(+)-α-methoxy-α-(trifluoromethyl)phenylacetyl chloride (5 μL) was added into the other vial, and stirred for 3 h. ^1^NMR and ^1^H, ^1^H COSY of the samples were recorded after wards.

### 3.7. Antimicrobial Assay

Minimum Inhibition Concentrations (MIC) against different test organisms were determined in serial dilution assay as described previously [17] against *Candida tenuis* MUCL 29982, *Mucor plumbeus* MUCL 49355, *Escherichia coli* DSM498 and *Bacillus subtilis* DSM10. The assays were carried out in 96-well microtiter plates in YMG media for filamentous fungi and yeast and MH for bacteria. The stock solution concentration was 300 μg/mL.

### 3.8. Cytotoxicity Assay

In vitro cytotoxicity (IC_50_) of the pure compounds **1**–**5** was determined against a panel of mammalian cell lines including mouse fibroblast L929, HeLa (KB-3-1), epidermoid carcinoma cells A431, breast cancer cells MCF-7, prostate cancer cells PC-3 and adenocarcinomic human alveolar basal epithelial cells A549. The cell lines were cultured in DMEM (Gibco, ThermoFisher Scientific, Hilden, Germany) and MCF-7 in RPMI (Lonza, Cologne, Germany) media, all supplemented with 10% of fetal bovine serum (Gibco) under 10% CO_2_ at 37 °C. The cytotoxicity assay was performed according to the MTT (3-(4,5-dimethylthiazol-2-yl)-2,5 diphenyltetrazolium bromide) method in 96-well microplates (ThermoFisher Scientific). Briefly 60 μL aliquots of serial dilutions from an initial stock of 1 mg/mL in MeOH of the test compounds were added to 120 μL aliquots of a cell suspension (5.0 × 10^4^ cells/mL) in 96-well microplates. After 5 days incubation, a MTT assay was performed, and the absorbance measured at 590 nm using an ELISA plate reader (Victor, PerkinElmer, Überlingen, Germany). The concentration at which the growth of cells was inhibited to 50% of the control (IC_50_) was obtained from the dose response curves. The negative control was methanol.

### 3.9. Nematicidal Assay

Compounds **1**–**5** were assessed for nematicidal activity against *Caenorhabditis elegans* according to [18] with slight modifications. *Caenorhabditis elegans* were inoculated monoxenically on nematode agar at room temperature for 4–5 days. Thereafter, nematodes were washed down from the plates with M9 buffer. The final nematodes concentration was adjusted to 500 nematodes/mL of M9 buffer. Assay was performed in 24-well microtiter plate at four different concentration (100, 50, 25 and 12.5 µ/mL) of each compound. Ivermectin was used as the positive control and methanol as a negative control. The plates were incubated at 20 °C in the shaker in the dark and nematicidal activity was recorded after 18 h of incubation and expressed as a LD_90_.

## 4. Conclusions

In our continuous search for novel and bioactive compounds from tropical basidiomycetes, we found five novel steroids from mycelial cultures of *Fomitiporia aethiopica*. The metabolites are the first steroids from the genus in the current circumscription, even though triterpenoids and steroids in particular are of widespread occurrence in Basidiomycota. The new metabolites were tested in various bioassays, but only moderate to weak cytotoxic activities were observed, and their biological functions remain obscure. Although closely related pregnane-type steroids have been reported before from *Phellinus igniarius* and the marine alga-derived fungus *Phaeosphaeria spartinae*, such pregnenolone-like compounds are unprecedented in fungal metabolism [19,20]. Accumulating evidence on triterpenoids broad spectrum pharmacological activities coupled with a low toxicity profile has sparked discussion with regard to their application, especially in cancer treatment.

## Figures and Tables

**Table 1:** NMR data for compounds **1** (in acetone-*d*_6_) and **2** (in DMSO-*d*_6_).

	1			2
Position	∂_C_, Type	∂_H_ (*J* in Hz)	∂_C_, Type	∂_H_ (*J* in Hz)
1	30.5, CH_2_	β:1.31, m ^b^; α:1.48, m ^b^	24.2, CH_2_	β:1.01, m ^b^; α:2.27, m ^b^
2	31.6, CH_2_	β:1.32, m ^b^; α:1.77, m ^b^	27.4, CH_2_	α:1.52, m ^b^, β:1.40, m ^b^
3	70.3, CH	3.50, tt, (4.5, 11.3)	63.1, CH	3.91, m
4	31.5, CH_2_	β:2.05, m ^b^; α:2.12 m ^b^	27.9, CH_2_	β:1.35, m ^b^; α:1.80, m ^b^
5	47.2, CH	2.90, dd, (12.2, 3.8)	41.4, CH	3.17 dd, (12.05, 3.9)
6	199.3, C		200.7, C	-
7	124.2, CH	5.48, d, (2.3)	122.6, CH	5.39, d(1.9)
8	160.8, C		160.4, C	
9	74.2, C		72.6, C	
10	42.7, C		41.9, C	
11	28.5, CH_2_	α:1.84, m ^b^; β:2.01, m ^b^	26.8, CH_2_	α:1.69, dd, (13.6, 3.9); β:1.78, dd, (13.6, 4.43)
12	35.5, CH_2_	β:1.97, m ^b^, α:2.07, m ^b^	34.0, CH_2_	β:1.83, m; α:1.94, m
13	46.8, C		45.6, C	
14	50.1, CH	3.12, ddd, (12.8, 6.7, 2.3)	48.8, CH	3.01, ddd, (12.4, 6.6, 1.9)
15	35.1, CH_2_	β:1.61, m ^b^; α:2.02, m ^b^	33.8, CH_2_	α:1.47, m ^b^; α:1.88, m ^b^
16	71.6, CH	4.74, bt, (3.0)	69.9, CH	4.55, bt, (3.1)
17	74.3, CH	2.71, d, (6.0)	73.0, CH	2.63, d, (6.1)
18	14.8, CH_3_	0.58, s	14.2, CH_2_	0.46, s
19	17.2, CH_3_	0.93, s	15.8, CH_3_	0.80, s
20	207.6, C		207.7, C	
21	31.9, CH_3_	2.16, s	31.6, CH_3_	2.15, s

^b^ Signals partially obscured, b- broad.

**Table 2:** NMR data for compounds **3**–**5** in acetone-*d*_6_.

3			4		5	
Pos.	∂_C_, Type	∂_H_ (*J* in Hz)	∂_C_, Type	∂_H_ (*J* in Hz)	∂_C_, Type	∂_H_ (*J* in Hz)
1.	30.4, CH_2_	β:1.29, m ^b^; α:1.49, m ^b^	25.3, CH_2_	β:1.16, m ^b^; α:2.47, m ^b^	32.2, CH_2_	β:1.82, m ^b^; α:1.91, m ^b^
2.	30.4, CH_2_	β:1.33 m ^b^; α:1.77, m ^b^	28.7, CH_2_	α:1.60, m ^b^; β:1.65, m ^b^	37.5, CH_2_	β:2.26, m ^b^; α:2.35, m ^b^
3.	70.3, CH	3.47, tt, (4.4, 11.3)	65.0, CH	4.05, m	210.1, CH	
4.	31.5, CH_2_	β:2.10, m ^b^; α:2.13 m ^b^	29.2, CH_2_	β:1.54, m ^b^; α:1.97, m ^b^	37.6, CH_2_	α:2.39, m ^b^; β:2.51, m ^b^
5.	47.3, CH	2.91, dd, (12.2, 3.8)	42.7, CH	3.32, dd, (12.3, 4.1)	49.0, CH	3.30, dd, (12.7, 4.9)
6.	199.4, C		201.1, C		198.4, C	
7.	123.4, CH	5.60, d, (2.1)	123.5, CH	5.58, d, (2.2)	123.1, CH	5.68, d, (2.2)
8.	160.2, C		160.0, C		160.8, C	
9.	74.4, C		74.5, C		74.6, C	
10.	42.8, C		43.4, C		43.1, C	
11.	31.3, CH_2_	α:1.77, m ^b^; β:2.15, m ^b^	29.4, CH_2_	β:1.84, m ^b^; α:2.13, m ^b^	29.6, CH_2_	α:1.92, m ^b^; β:2.24, m ^b^
12.	32.1, CH_2_	β:1.81, m ^b^; α:2.30 m ^b^	32.1, CH_2_	β:1.80, m ^b^; α:2.32, m ^b^	32.1, CH_2_	β:1.82, m ^b^; α:2.35, m ^b^
13.	46.8, C		49.0, C		49.0, C	
14.	52.9, CH	3.11, ddd, (11.6, 6.6, 2.1)	52.7, CH	3.13, ddd, (11.6, 6.5, 2.2)	52.7, CH	3.14, ddd (11.6, 2.2 Hz, 6.2)
15.	31.3, CH_2_	β:2.39, m; α:2.47, m	31.3, CH_2_	β:2.41, m; α:2.47, m	31.3, CH_2_	β:2.42, m; α:2.51, m
16.	144.3, CH	6.91, dd, (1.9, 3.4)	144.3, CH	6.91, dd, (1.9, 3.2)	144.3, CH	6.92, dd, (3.4, 1.9)
17.	155.1, C		155.2, C		155.1, C	
18.	16.4, CH_3_	0.88, s	16.4, CH_3_	0.88, s	16.4, CH_3_	0.91, s
19.	17.2, CH_3_	0.99, s	16.5, CH_3_	0.99, s	16.5, CH_3_	1.24, s
	196.3, C		196.3, C	-	196.3, C	
	27.14, CH_3_	2.25, s	27.2, CH_3_	2.26, s	27.1, CH_3_	2.26, s

^b^ Signals partially obscured.

**Table 3:** Cytotoxic activities of compounds **1**–**5**.

Cell Lines	Cytotoxicity IC_50_ (μg/mL)					
	1	2	3	4	5	Epothilon B
L929	28	45	40	45	40	0.0014
KB3.1	19	39	35	39	33	0.00022
A431	22	-	27	21	14	0.0006
A549	nt	-	70	52	43	0.005
PC-3	8	-	45	40	39	0.0002
SKOV-3	26	-	38	36	34	0.0014
MCF-7	20	-	18	17	16	0.0004

Not active, nt- not tested.

## Supplementary Materials

UV, HRMS, ^1^H- and ^13^C-NMR, ^1^H-^1^H COSY, HSQC, and HMBC and ROESY spectra of compounds **1–5** and data on the producing organism are available as Supplementary Material.
