# Supplementary material for: Aethiopinolones A–E, New Pregnenolone Type Steroids from the East African Basidiomycete Fomitiporia aethiopica
Source: Molecules. 2018 Feb 9;23(2):369. doi: 10.3390/molecules23020369 (PMC6017562; doi:10.3390/molecules23020369)

Supporting information

Aethiopinolones A-E, new pregnenolone type steroids from the East African basidiomycete *Fomitiporia aethiopica*

Clara Chepkirui [1,†], Winnie C. Sum [2,†], Tian Cheng[1], Josphat C. Matasyoh [3], Cony Decock [4] and Marc Stadler [1,*]

[1] Department of Microbial Drugs, Helmholtz Centre for Infection Research and German Centre for Infection Research (DZIF), partner site Hannover/Braunschweig, Inhoffenstrasse 7, 38124 Braunschweig, Germany;

[2] Egerton University, Department of Biochemistry, P.O BOX 536, 20115, Njoro, Kenya

[3] Egerton University, Department of Chemistry, P.O BOX 536, 20115, Njoro, Kenya

[4] Mycothéque de l′ Universite catholique de Louvain (BCCM/MUCL), Place Croix du Sud 3, B-1348 Louvain-la-Neuve, Belgium

* Correspondence: marc.stadler@helmholtz-hzi.de; Tel.: +49 531 6181-4240; Fax: 49 531 6181-9499

† These authors contributed equally to this work.

Contents

LIST OF TABLES iii

LIST OF FIGURES iv

1 and 2D NMR data for Aethiopinolone A (1) 1

Figure 1:^{1}H NMR spectrum of Aethiopinolone A (1) in acetone-d_6 (700 MHz) 1

1 and 2D NMR data for Aethiopinolone B (2).................... 7

1 and 2D NMR data for Aethiopinolone C (3) 11

1 and 2D NMR data for Aethiopinolone D (4) 17

1 and 2D NMR data for Aethiopinolone E (5).................... 23

ITS sequences of the producing organism.................... 28

Pictures of the herbarium and the culture of *Fomitiporia aethiopica*.................... 28

LIST OF TABLES

Table 1: *S*-MTPA ester and *R*-MTPA ester chemical shifts analysis for Aethiopinolone A (1) 27
Table 2: *S*-MTPA ester and *R*-MTPA ester chemical shifts analysis for Aethiopinolone C (3) 27
Table 3: *S*-MTPA ester and *R*-MTPA ester chemical shifts analysis for Aethiopinolone D (4) 27

LIST OF FIGURES

Figure 1: ^{1}H NMR spectrum of Aethiopinolone A (1) in acetone-d_6 (700 MHz) 1
Figure 2: ^{13}C NMR spectrum of Aethiopinolone A (1) in acetone-d_6 (175 MHz) 1
Figure 3:DEPT spectrum of Aethiopinolone A (1) in acetone-d_6 (175 MHz) 2
Figure 4: ^{1}H, ^{13}C HSQC spectrum of Aethiopinolone A (1) in acetone-d_6 (700 MHz, 175MHz) 2
Figure 5: ^{1}H, ^{13}C HMBC spectrum of Aethiopinolone A (1) in acetone-d_6 (700 MHz, 175 MHz). 3
Figure 6: ^{1}H, ^{1}H COSY spectrum of Aethiopinolone A (1) in acetone-d_6 (500 MHz): 3
Figure 7: ^{1}H, ^{1}H ROESY spectrum of Aethiopinolone A (1) in acetone-d_6 (500 MHz) 4
Figure 8: HRMS spectrum of Aethiopinolone A (1) 4
Figure 9: ^{1}H NMR spectrum of Aethiopinolone A (1) (*S*)- MTPA ester in chloroform –d (700 MHz) 5
Figure 10: ^{1}H,^{1}H COSY NMR spectrum of Aethiopinolone A (1) (*S*)- MTPA ester in chloroform –d (700 MHz) 5
Figure 11: ^{1}H NMR spectrum of Aethiopinolone A (1) (*R*)- MTPA ester in chloroform –d (700 MHz) 6
Figure 12: ^{1}H,^{1}H COSY NMR spectrum of Aethiopinolone A (1) (*R*)- MTPA ester in chloroform –d (700 MHz) 6
Figure 13: ^{1}H NMR spectrum of Aethiopinolone B (2) in DMSO-d_6 (500 MHz) 7
Figure 14: ^{13}C NMR spectrum of Aethiopinolone B (2) in in DMSO-d_6 (125 MHz) 7
Figure 15: DEPT NMR spectrum of Aethiopinolone B (2) in in DMSO-d_6 (125 MHz) 8
Figure 16: ^{1}H, ^{13}C HSQC spectrum of Aethiopinolone B (2) in in DMSO-d_6 (500 MHz, 125 MHz) 8
Figure 17: ^{1}H, ^{13}C HMBC spectrum of Aethiopinolone B (2) in in DMSO-d_6 (500 MHz, 125 MHz) 9
Figure 18: ^{1}H, ^{1}H COSY spectrum of Aethiopinolone B (2) in in DMSO-d_6 (500 MHz) 9
Figure 19: ^{1}H, ^{1}H ROESY spectrum of Aethiopinolone B (2) in in DMSO-d_6 (500 MHz) 10
Figure 20: HRMS spectrum of Aethiopinolone B (2) 10
Figure 21: ^{1}H NMR spectrum of Aethiopinolone C (3) in acetone-d_6 (500 MHz) 11
Figure 22: ^{13}C NMR spectrum of Aethiopinolone C (3) in acetone-d_6 (125 MHz) 11
Figure 23: DEPT NMR spectrum of Aethiopinolone C (3) in acetone-d_6 (125 MHz) 12
Figure 24: ^{1}H, ^{13}C HSQC spectrum of Aethiopinolone C (3) in acetone-d_6 (500MHz, 125 MHz) 12
Figure 25: ^{1}H, ^{13}C HMBC spectrum of Aethiopinolone C (3) in acetone-d_6 (500MHz, 125 MHz) 13
Figure 26: ^{1}H,^{1}H COSY spectrum of Aethiopinolone C (**3**) in acetone-d_6 (500MHz) 13
Figure 27: ^{1}H, ^{1}H ROESY spectrum of Aethiopinolone C (3) in acetone-d_6 (500MHz) 14
Figure 28: HRMS data for Aethiopinolone C (3) 14
Figure 29: ^{1}H NMR spectrum of Aethiopinolone C (3) (*S*)- MTPA ester in pyridine –d_5 (700 MHz) 15

Figure 30:^1H, ^1HCOSY spectrum of Aethiopinolone C (3) (S)- MTPA ester in pyridine –d_5 (700 MHz) 15
Figure 31:^1H NMR spectrum of Aethiopinolone C (3) (*R*)- MTPA ester in pyridine –d_5 (700 MHz) 16
Figure 32:^1H, ^1HCOSY spectrum of Aethiopinolone C (3) (R)- MTPA ester in pyridine –d_5 (700 MHz) 16
Figure 33:^1H NMR spectrum for Aethiopinolone D (4) in acetone-d_6 (175 MHz) 17
Figure 34: ^{13}C NMR spectrum of Aethiopinolone D (4) in acetone-d_6 (175 MHz) 17
Figure 35: DEPT NMR spectrum of Aethiopinolone D (4) in acetone-d6 (175 MHz) Figure 36:^1H, ^{13}C HSQC spectrum of Aethiopinolone D (4) in acetone-d_6 (700 MHz, 175 MHz) 18
Figure 37:^1H,^{13}C HMBC spectrum of Aethiopinolone D (4) in acetone-d_6 (700 MHz, 175 MHz) 19
Figure 38:^1H, ^1H COSY spectrum of Aethiopinolone D (4) in acetone-d_6 (700 MHz) 19
Figure 39: ^1H, ^1H ROESY spectrum of Aethiopinolone D (4) in acetone-d_6 (700 MHz) 20
Figure 40: HRMS spectrum for Aethiopinolone D (4) 20
Figure 41:^1H NMR spectrum of Aethiopinolone D (4) (*S*)- MTPA ester in pyridine –d_5 (700 MHz) 21
Figure 42:^1H, ^1H COSY spectrum of Aethiopinolone D (4) (*S*)- MTPA ester in pyridine –d_5 (700 MHz) 21
Figure 43:^1H NMR spectrum of Aethiopinolone D (4) (R)- MTPA ester in pyridine –d_5 (700 MHz) 22
Figure 44:^1H, ^1H COSY spectrum of Aethiopinolone D (4) (*R*)- MTPA ester in pyridine –d_5 (700 MHz) 22
Figure 45:^1H NMR spectrum of Aethiopinolone E (5) in acetone-d_6 (700 MHz) 23
Figure 46:^{13}C NMR spectrum of Aethiopinolone E (5) in acetone-d_6 (175 MHz) 23
Figure 47: DEPT NMR spectrum of Aethiopinolone E (5) in acetone-d_6 (175 MHz) 24
Figure 48:^1H, ^{13}C HSQC NMR spectrum of Aethiopinolone E (5) in acetone-d_6 (700 MHz, 175 MHz) 24
Figure 49:^1H, ^{13}C HMBC NMR spectrum of Aethiopinolone E (5) in acetone-d_6 (700 MHz, 175 MHz) 25
Figure 50:^1H, ^1H COSY NMR spectrum of Aethiopinolone E (5) in acetone-d_6 (700 MHz) 25
Figure 51:^1H, ^1H ROESY NMR spectrum of Aethiopinolone E (5) in acetone-d_6 (700 MHz) 26
Figure 52: HRMS spectrum for Aethiopinolone **E** (**5**) 26

1 and 2D NMR data for Aethiopinolone A (1)

Figure 1:^{1}H NMR spectrum of Aethiopinolone A (**1**) in acetone-d₆ (700 MHz)

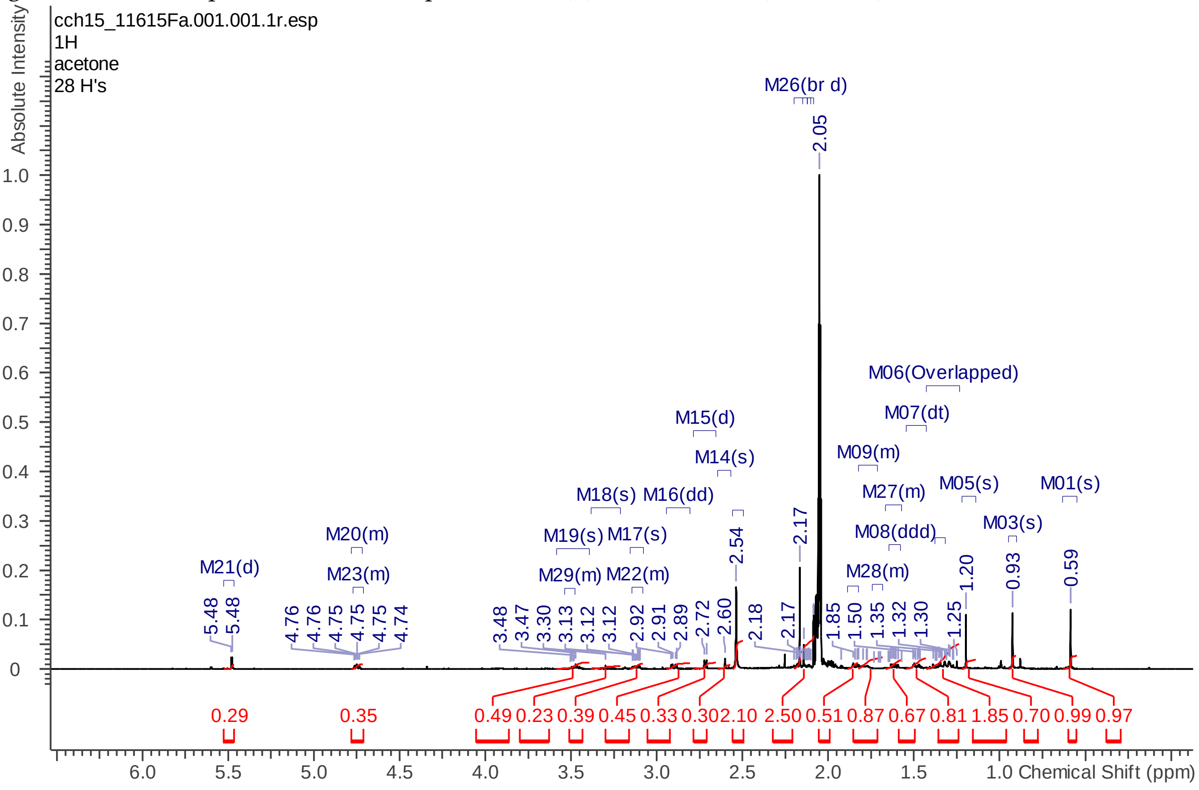

Figure 2:^{13}C NMR spectrum of Aethiopinolone A (**1**) in acetone-d₆ (175 MHz)

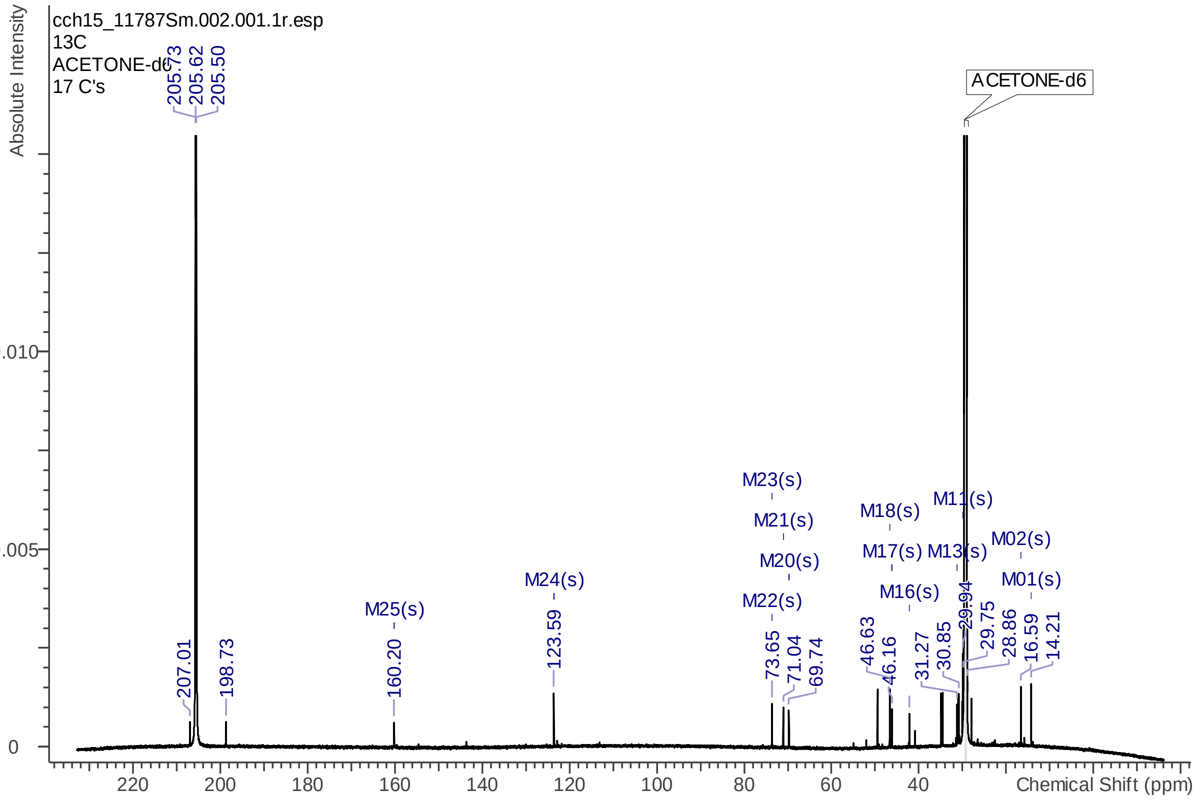

Figure 3:DEPT spectrum of Aethiopinolone A (**1**) in acetone-d₆ (175 MHz)

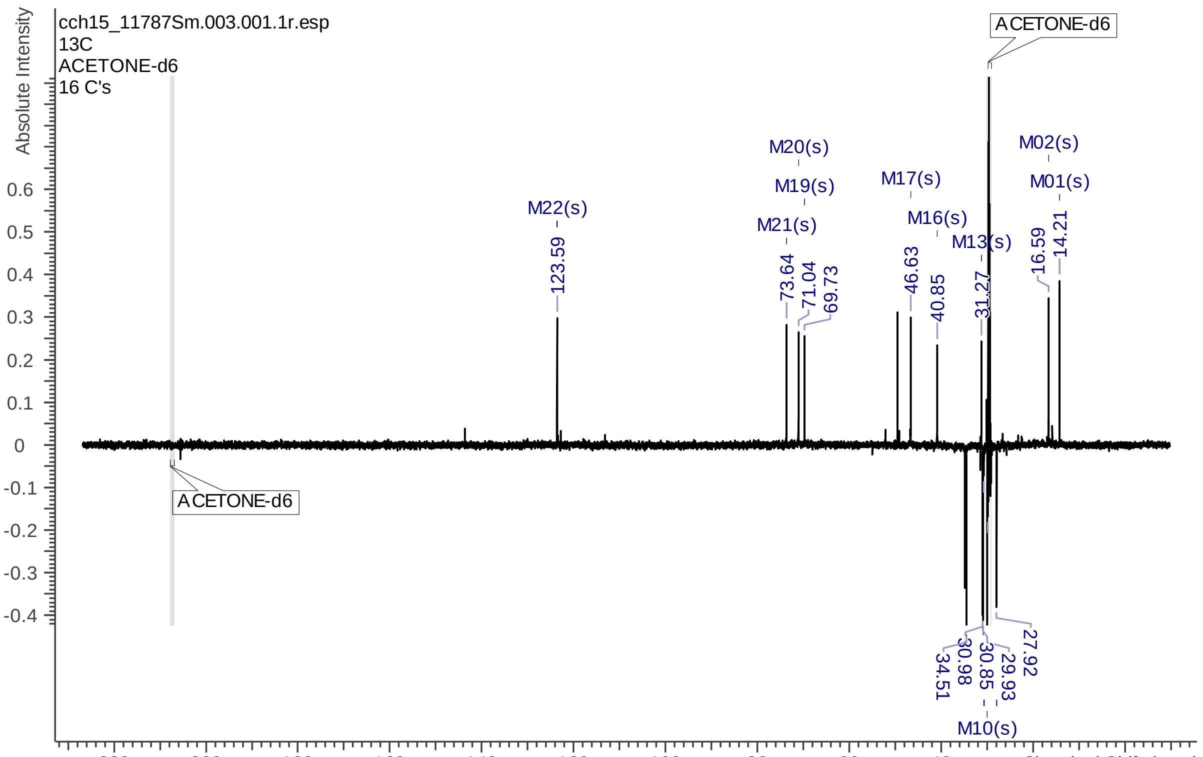

Figure 4:¹H, ¹³C HSQC spectrum of Aethiopinolone A (1) in acetone-d₆ (700 MHz, 175MHz)

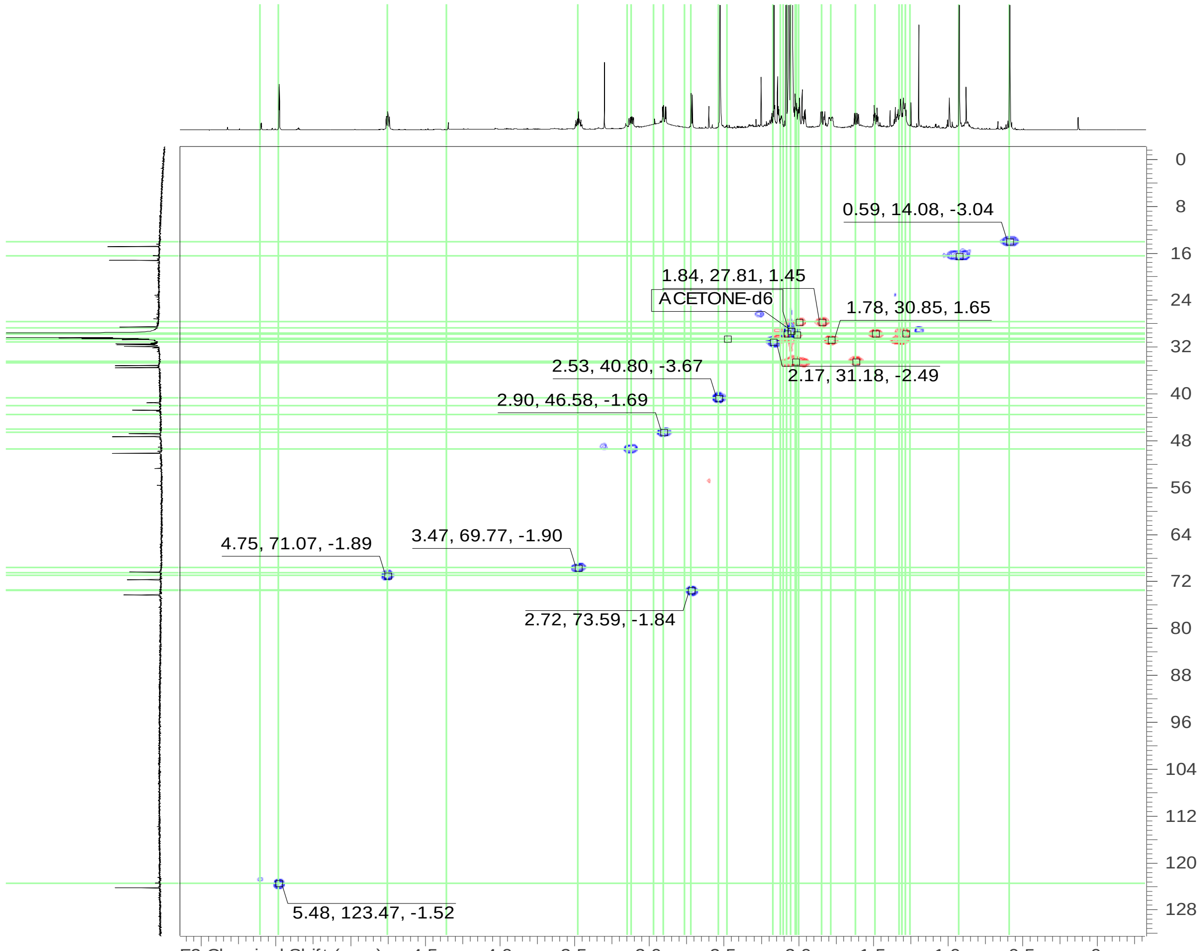

Figure 5:^{1}H, ^{13}C HMBC spectrum of Aethiopinolone A (**1**) in acetone-d6 (700 MHz, 175 MHz).

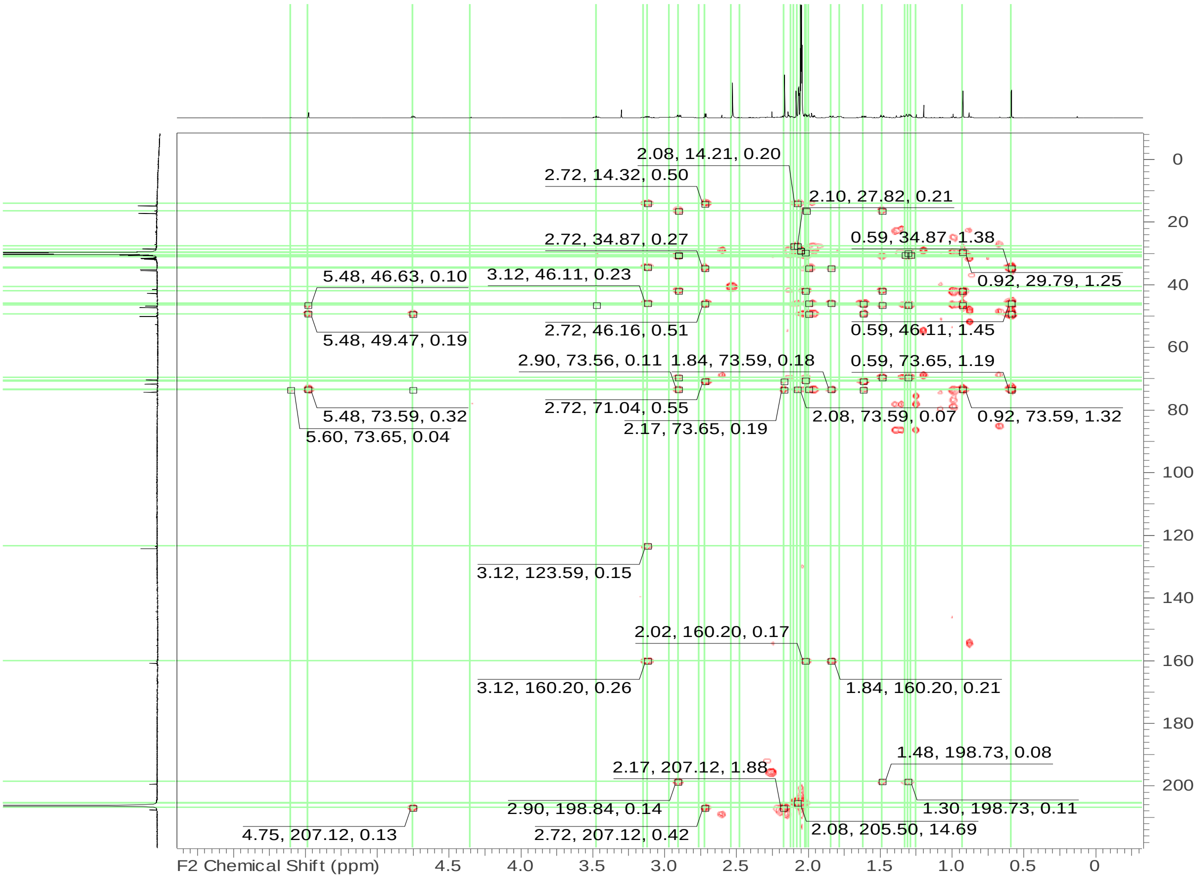

Figure 6:^{1}H, ^{1}H COSY spectrum of Aethiopinolone A (**1**) in acetone-d6 (500 MHz):

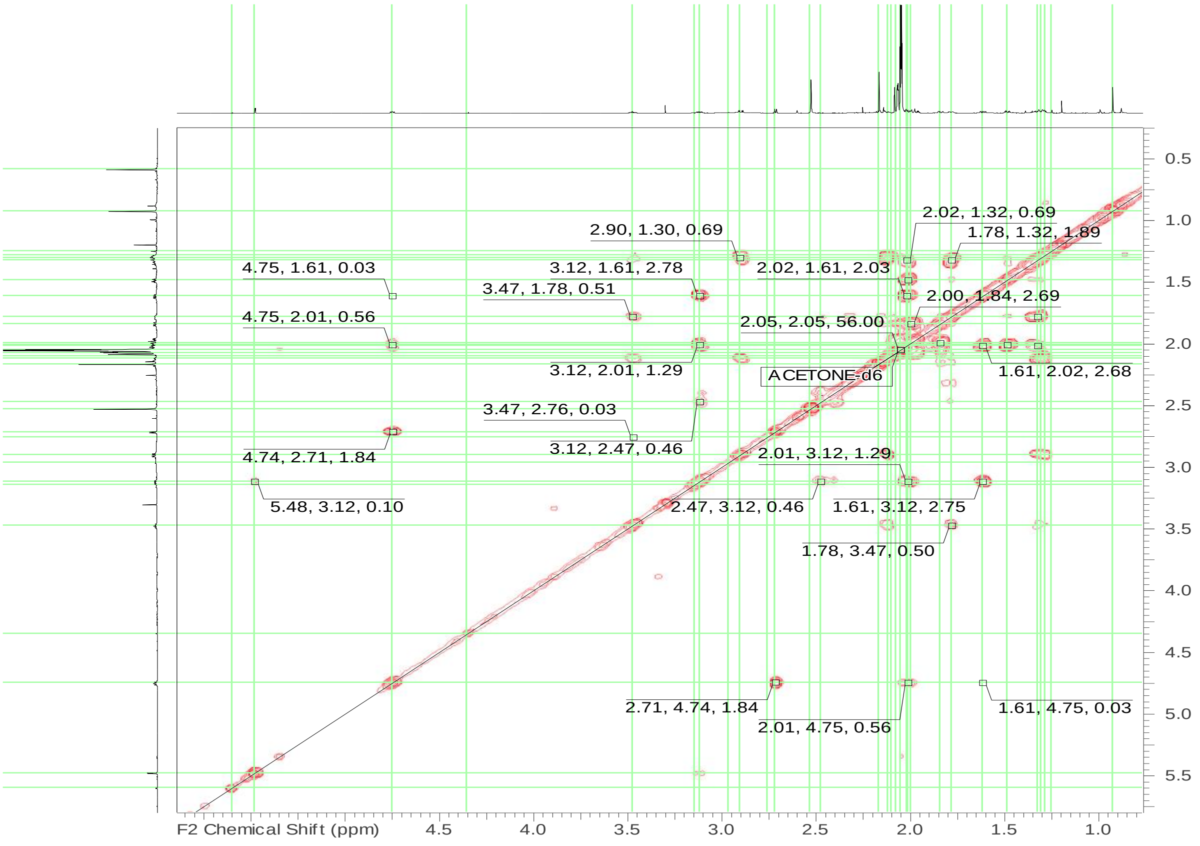

Figure 7:^{1}H, ^{1}H ROESY spectrum of Aethiopinolone A (**1**) in acetone-d_6 (500 MHz)

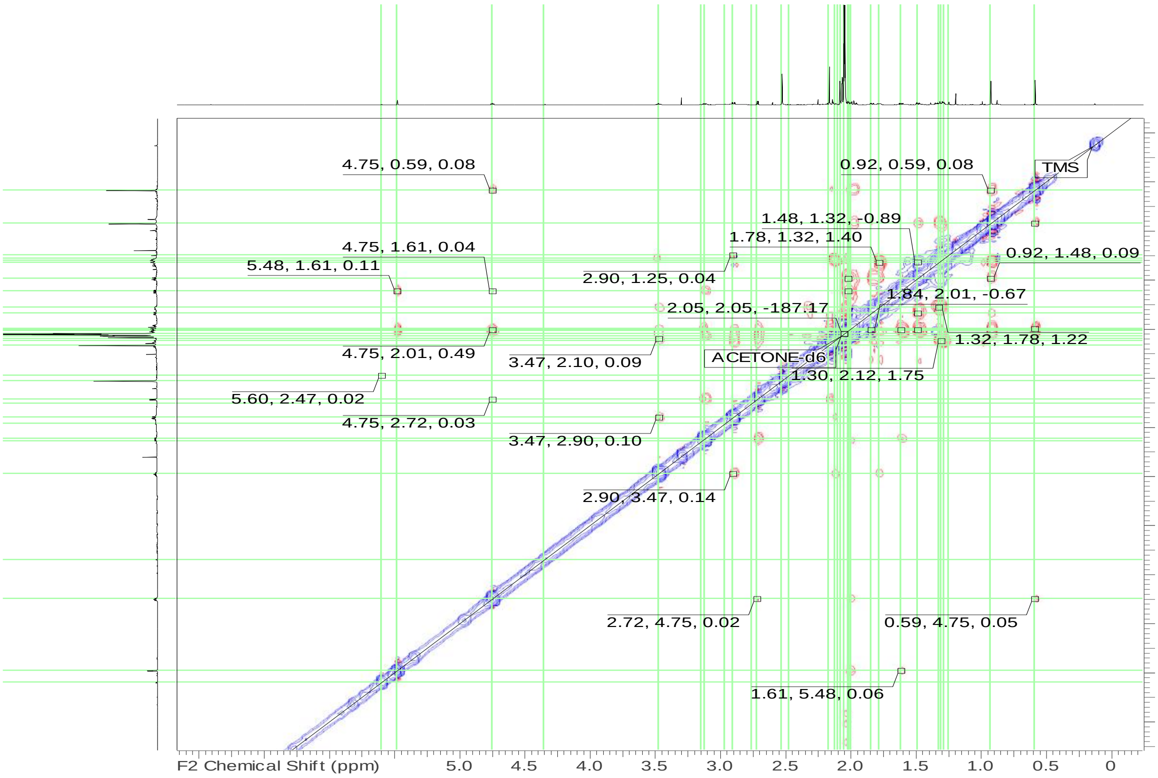

Figure 8: HRMS spectrum of Aethiopinolone A (**1**)

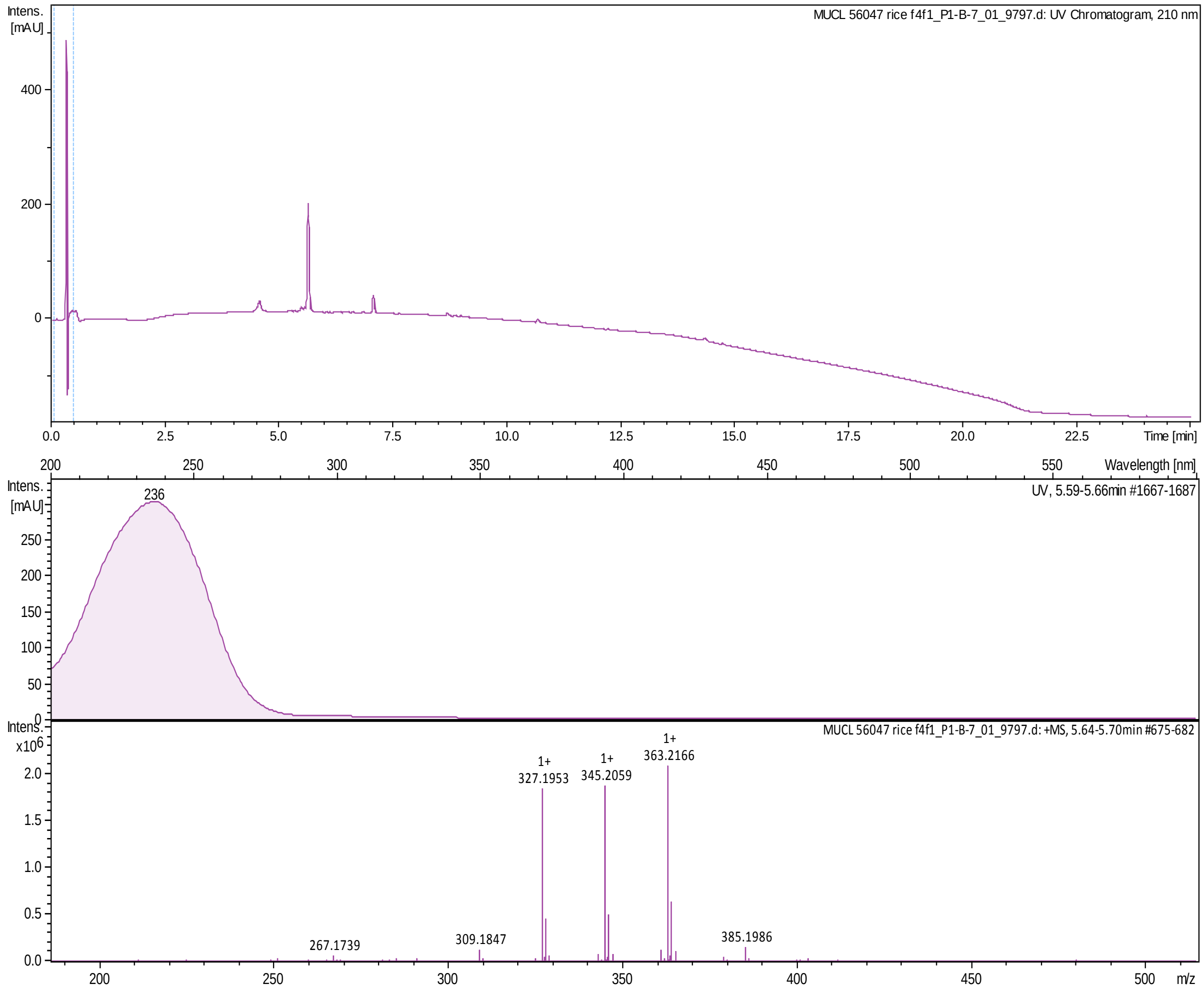

Figure 9: ^{1}H NMR spectrum of Aethiopinolone A (**1**) (*S*)- MTPA ester in chloroform –d (700 MHz)

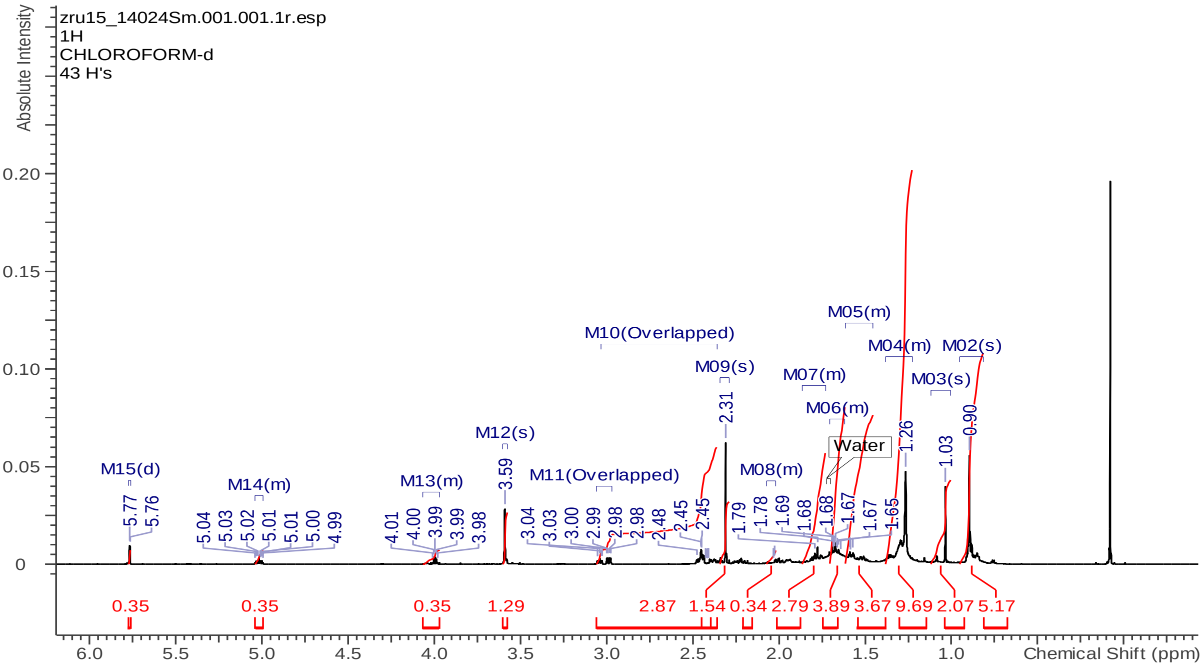

Figure 10: ^{1}H,^{1}H COSY NMR spectrum of Aethiopinolone A (**1**) (*S*)- MTPA ester in chloroform –d (700 MHz)

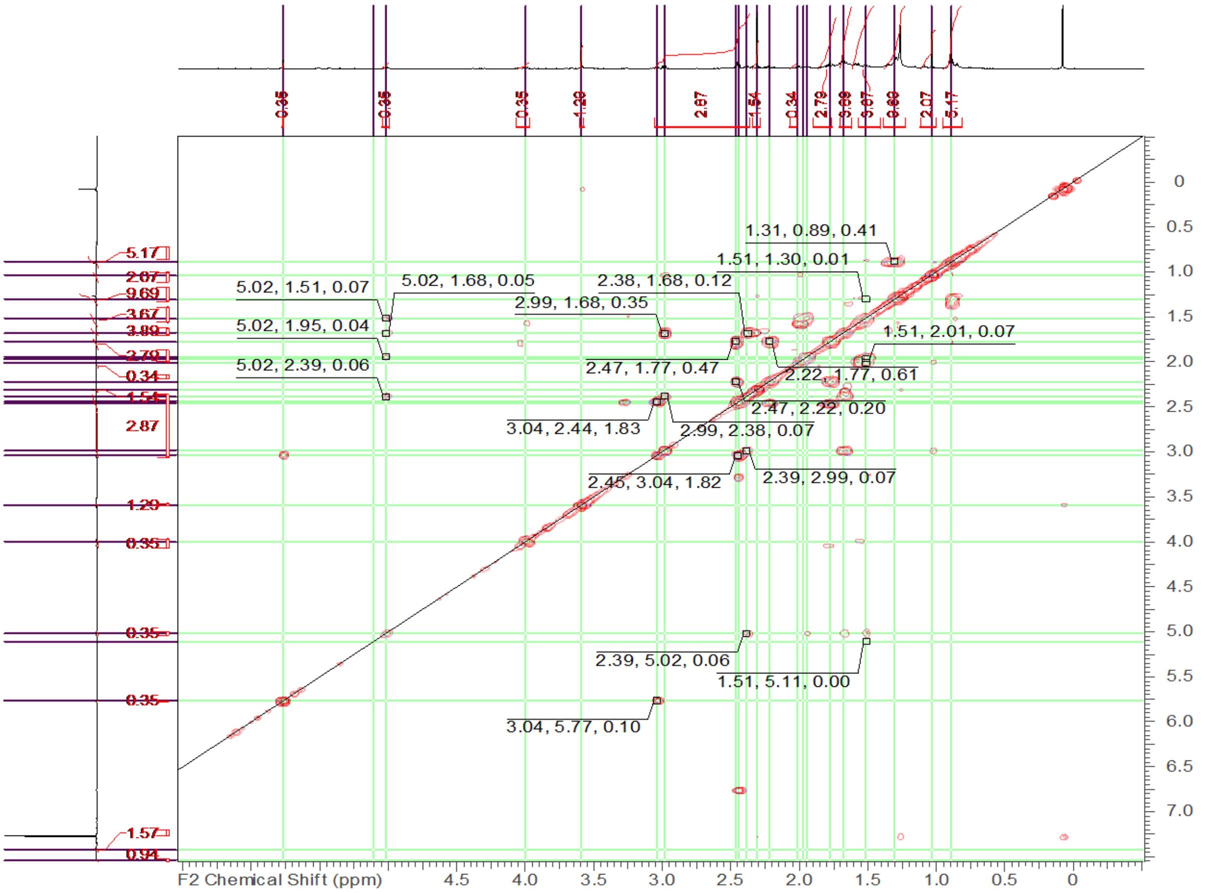

Figure 11: ^{1}H NMR spectrum of Aethiopinolone A (**1**) (*R*)- MTPA ester in chloroform –d (700 MHz)

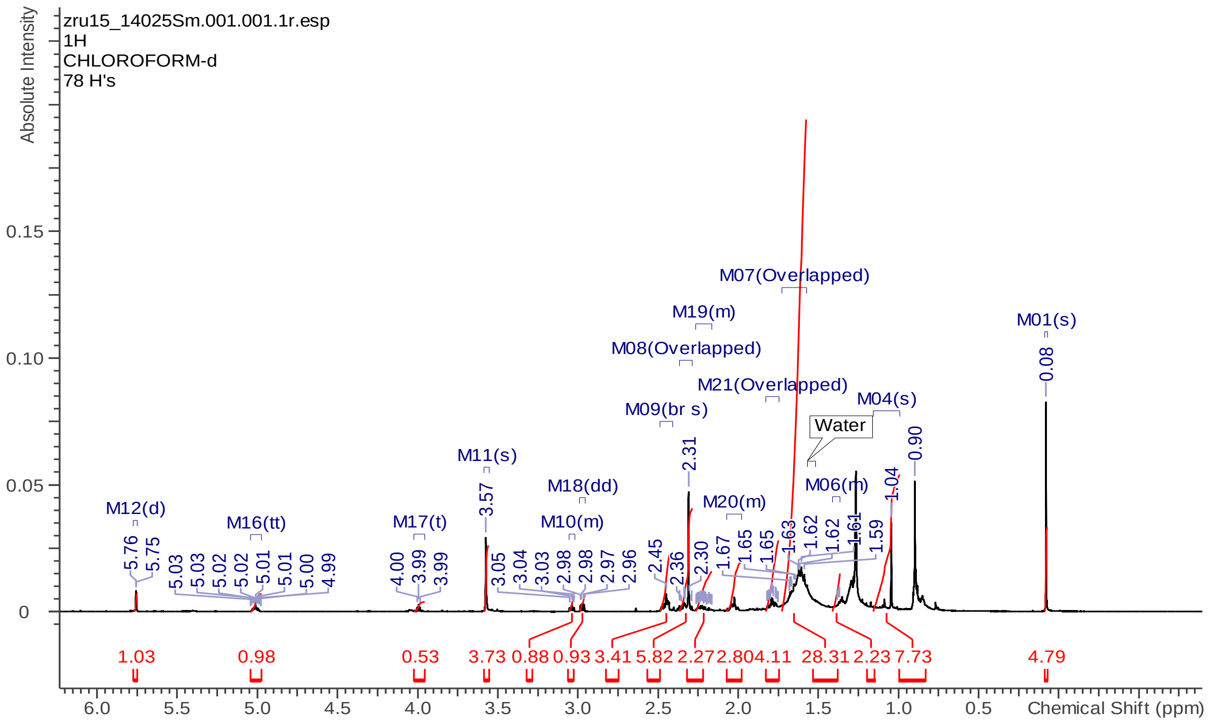

Figure 12: ^{1}H,^{1}H COSY NMR spectrum of Aethiopinolone A (**1**) (*R*)- MTPA ester in chloroform –d (700 MHz)

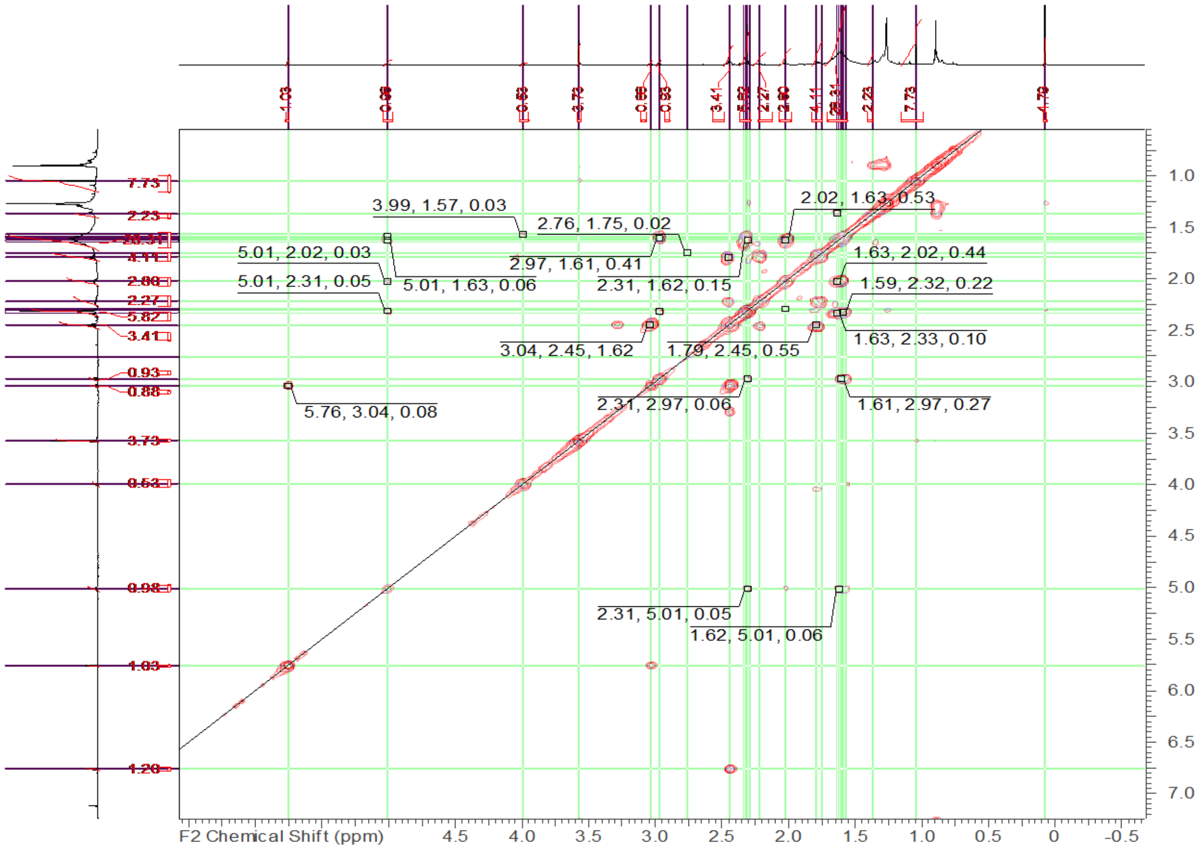

1 and 2D NMR data for Aethiopinolone B (2)

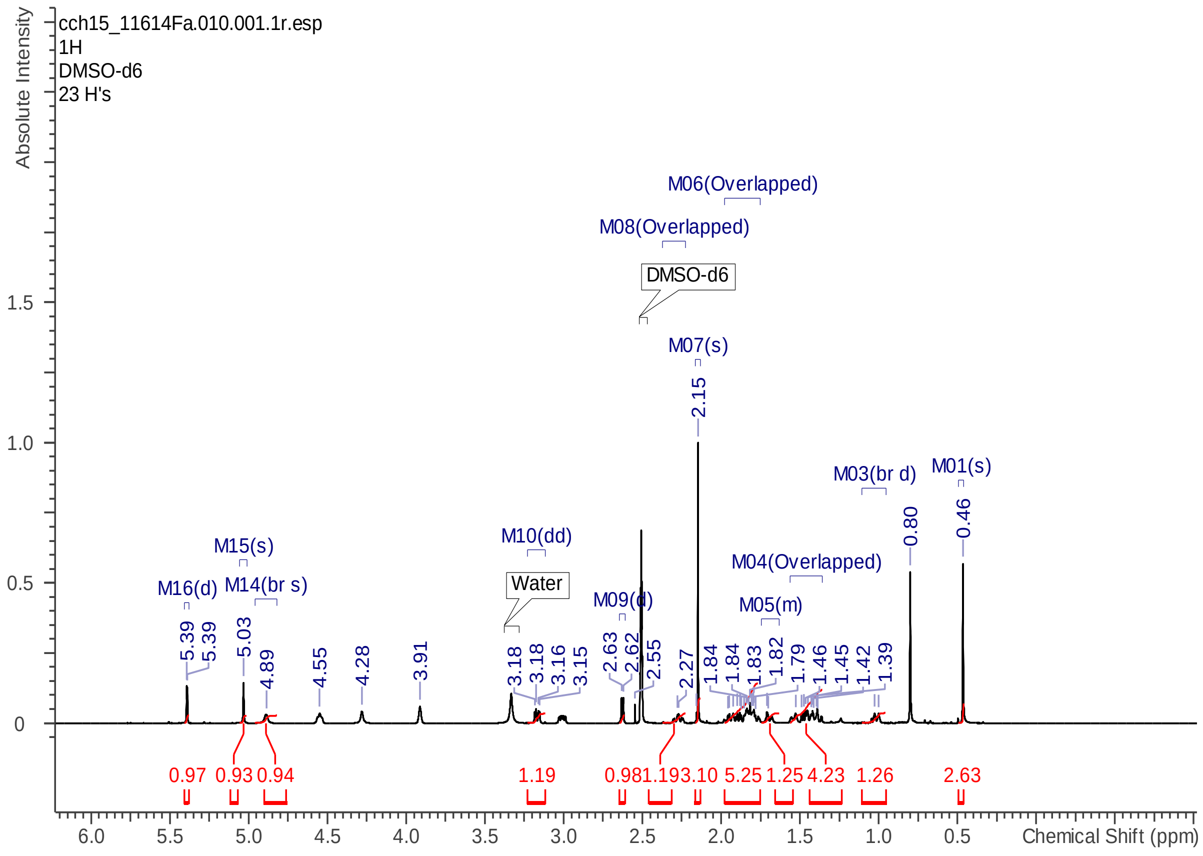

Figure 13: ^{1}H NMR spectrum of Aethiopinolone B (**2**) in DMSO-d_6 (500 MHz)

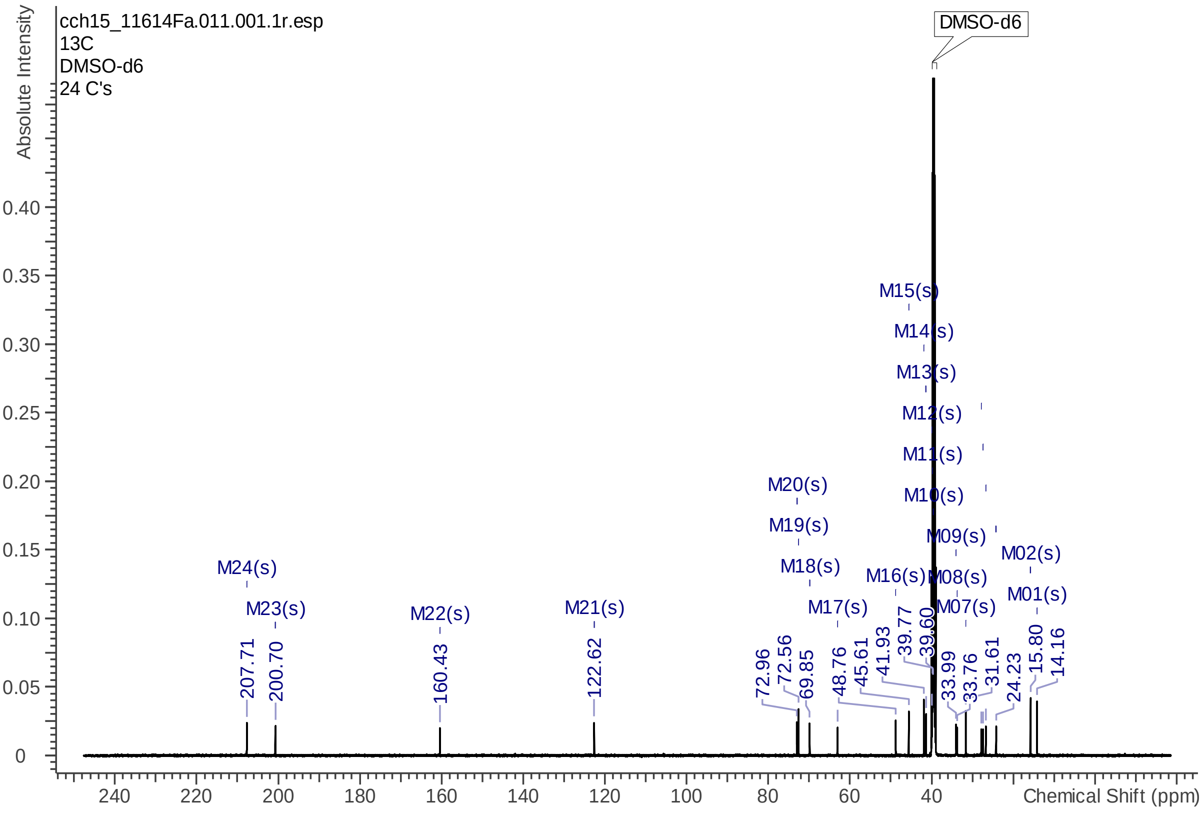

Figure 14: ^{13}C NMR spectrum of Aethiopinolone B (**2**) in in DMSO-d_6 (125 MHz)

Figure 15: DEPT NMR spectrum of Aethiopinolone B (**2**) in in DMSO-d_6 (125 MHz)

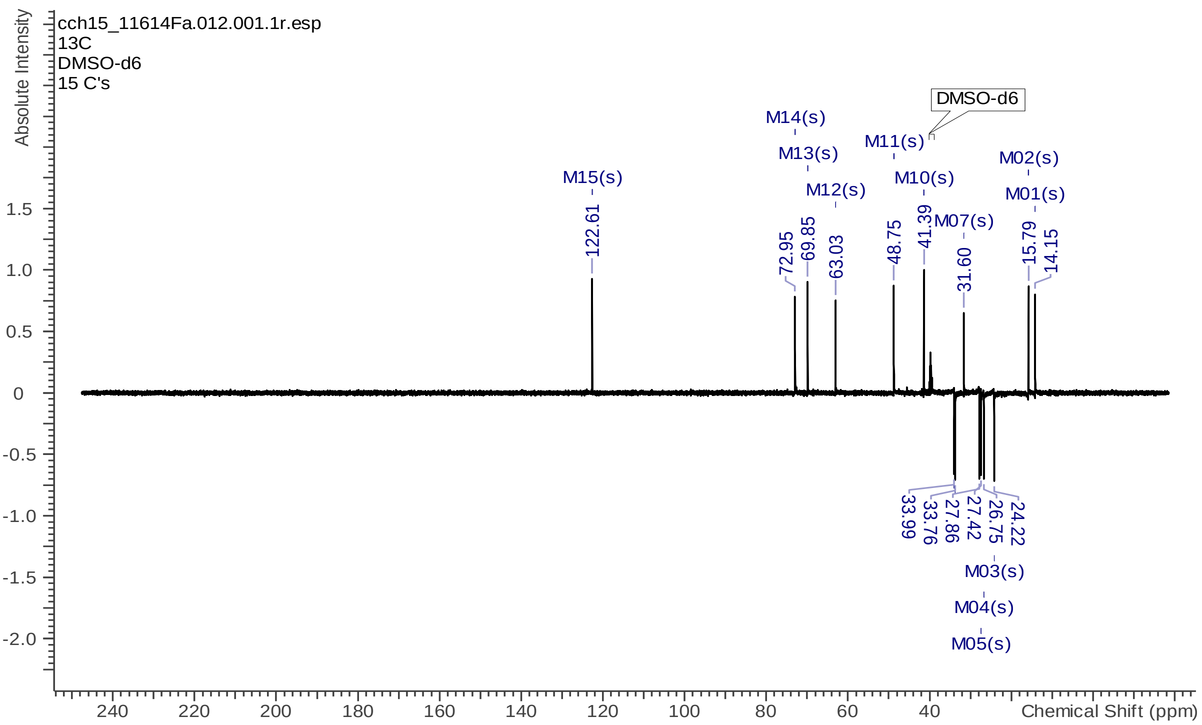

Figure 16: ^1H, ^{13}C HSQC spectrum of Aethiopinolone B (**2**) in in DMSO-d_6 (500 MHz, 125 MHz)

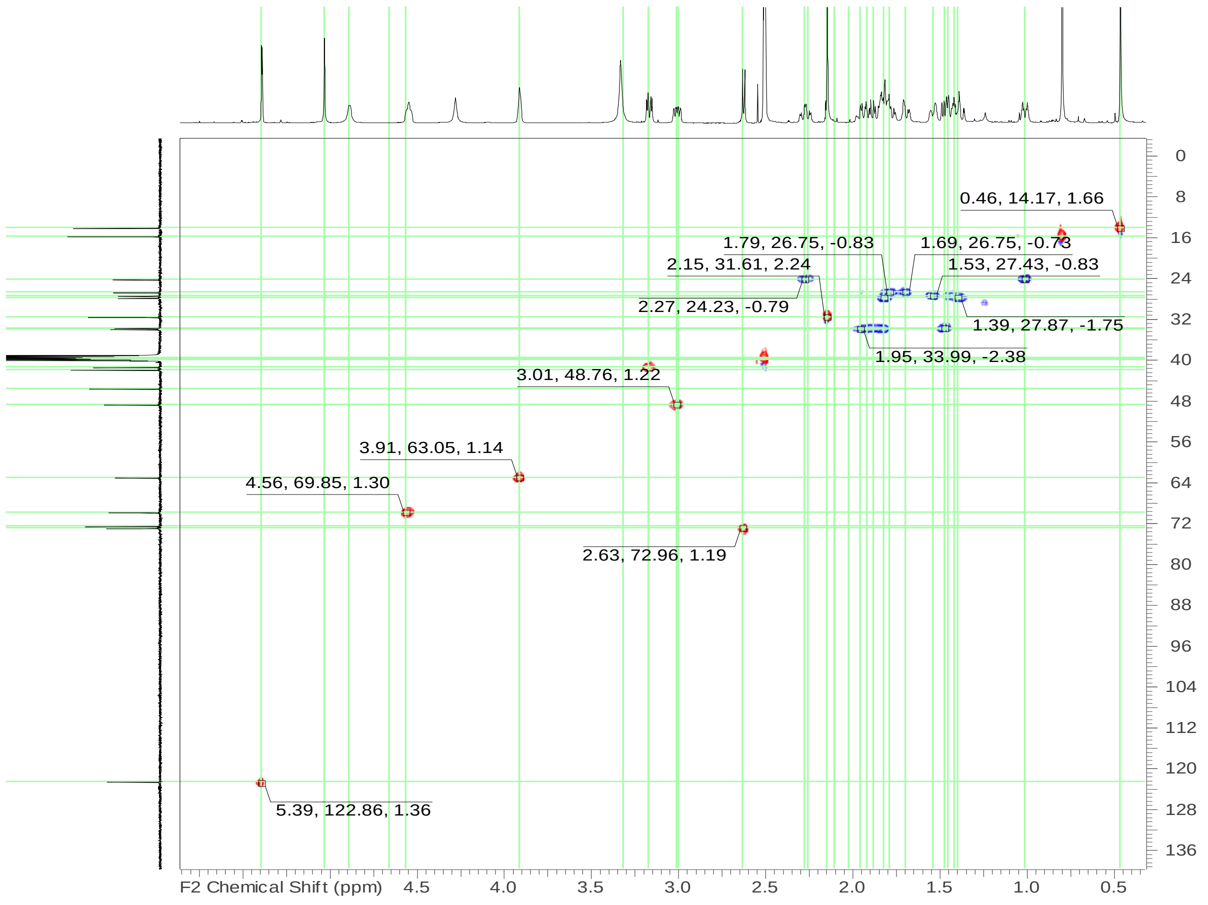

Figure 17: ¹H, ¹³C HMBC spectrum of Aethiopinolone B (**2**) in in DMSO-d₆ (500 MHz, 125 MHz)

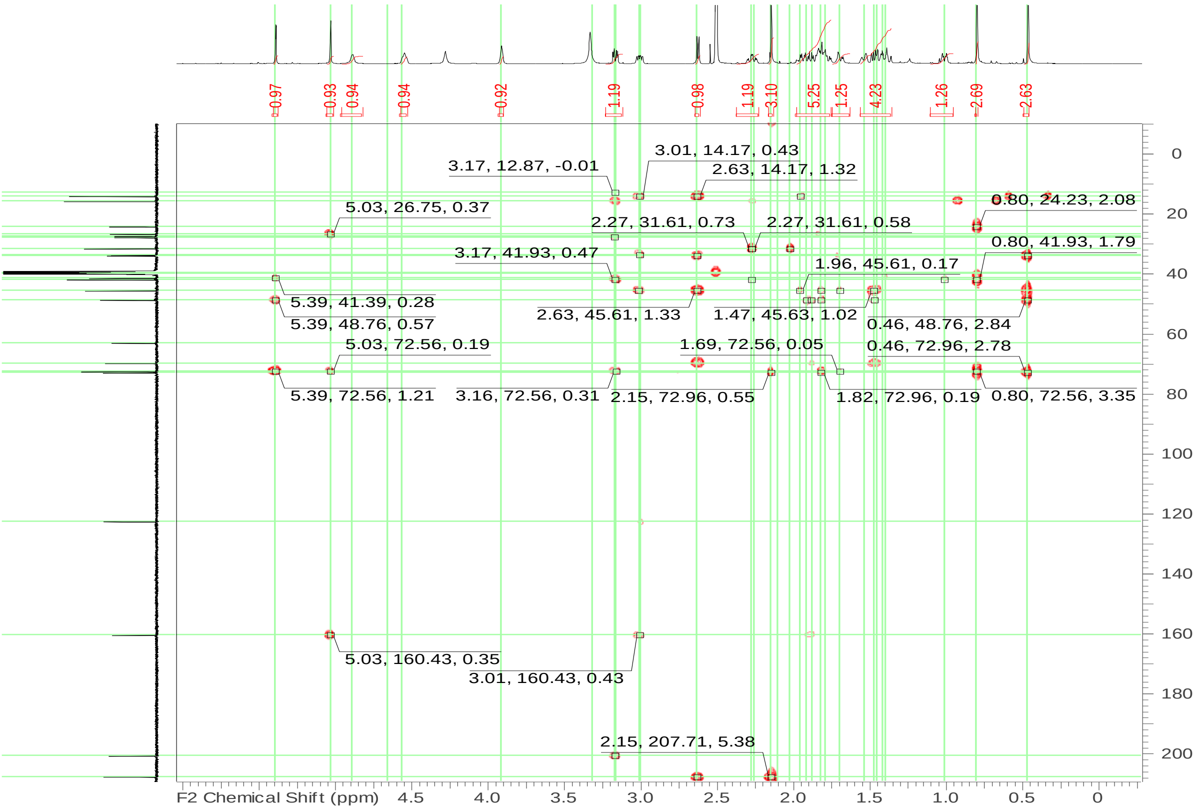

Figure 18: ¹H, ¹H COSY spectrum of Aethiopinolone B (**2**) in in DMSO-d₆ (500 MHz)

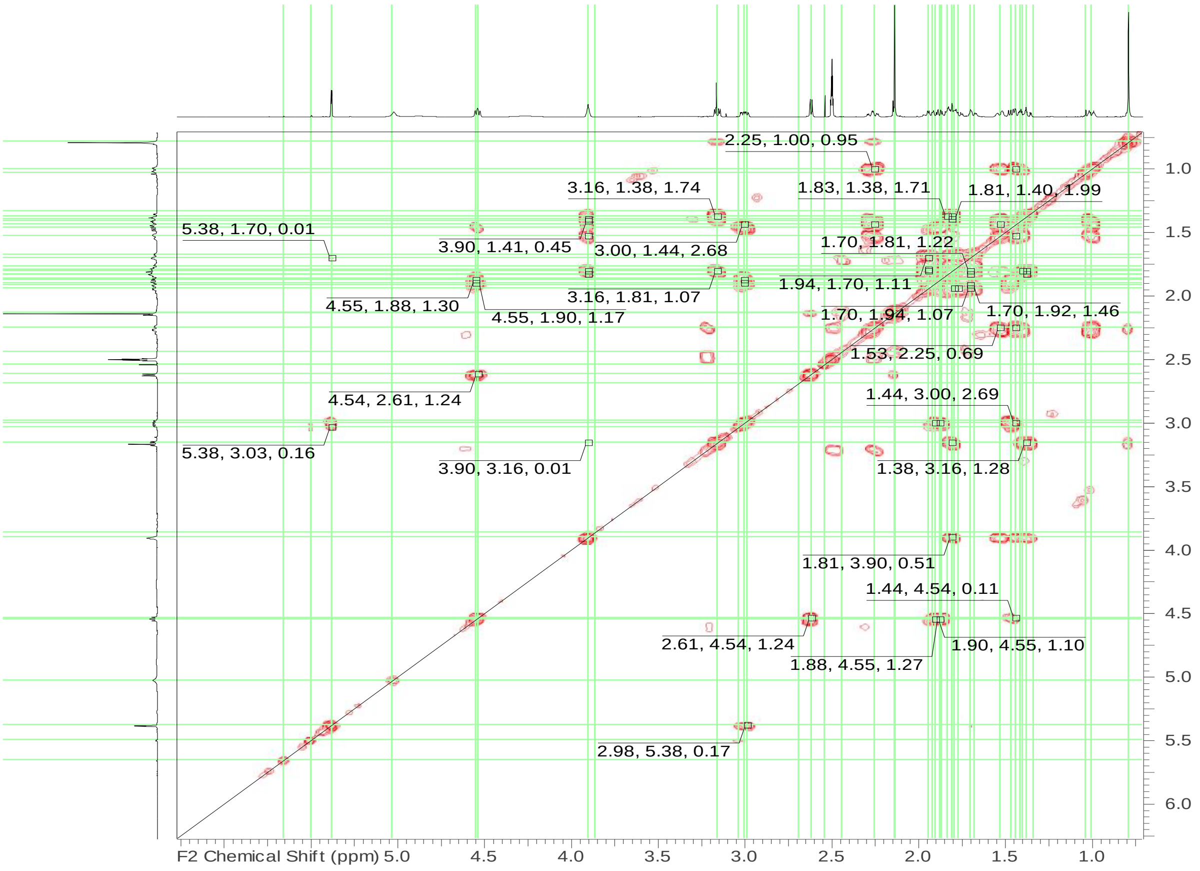

Figure 19:¹H, ¹H ROESY spectrum of Aethiopinolone B (**2**) in in DMSO-d₆ (500 MHz)

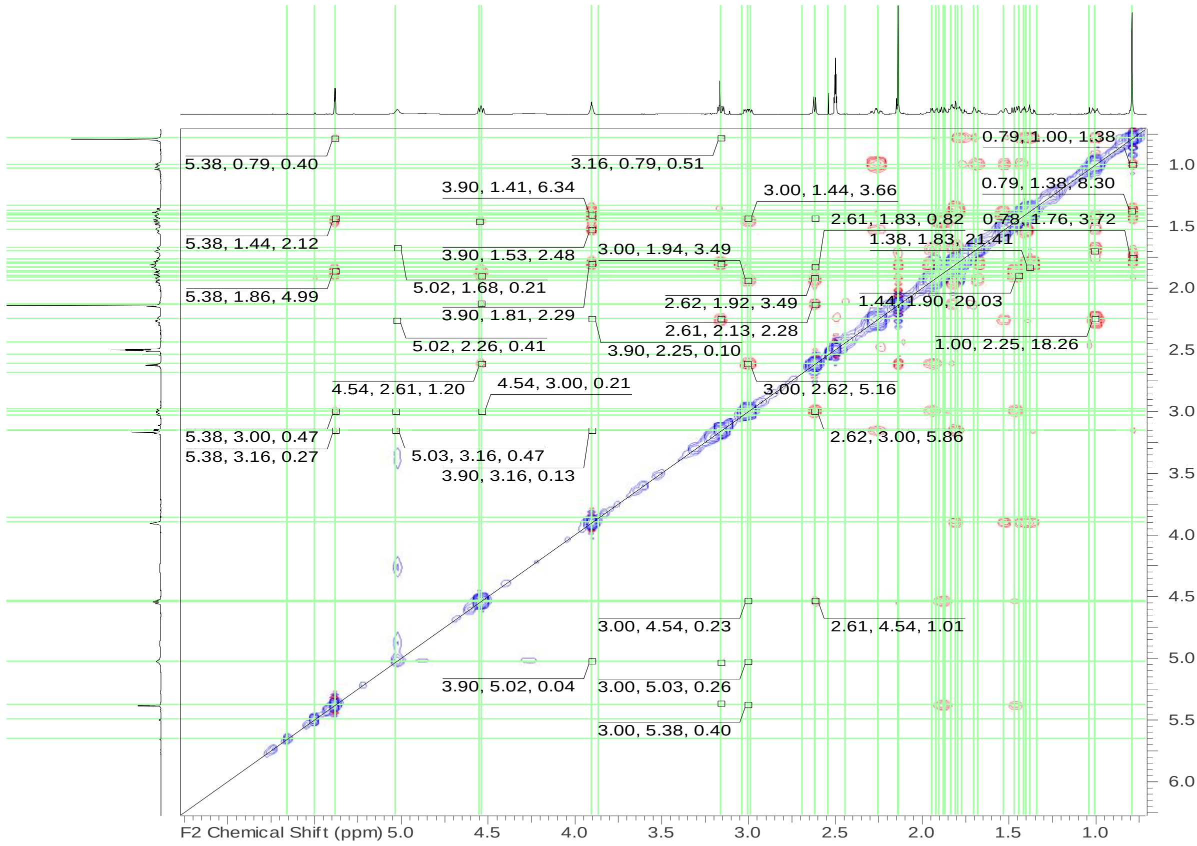

Figure 20: HRMS spectrum of Aethiopinolone B (**2**)

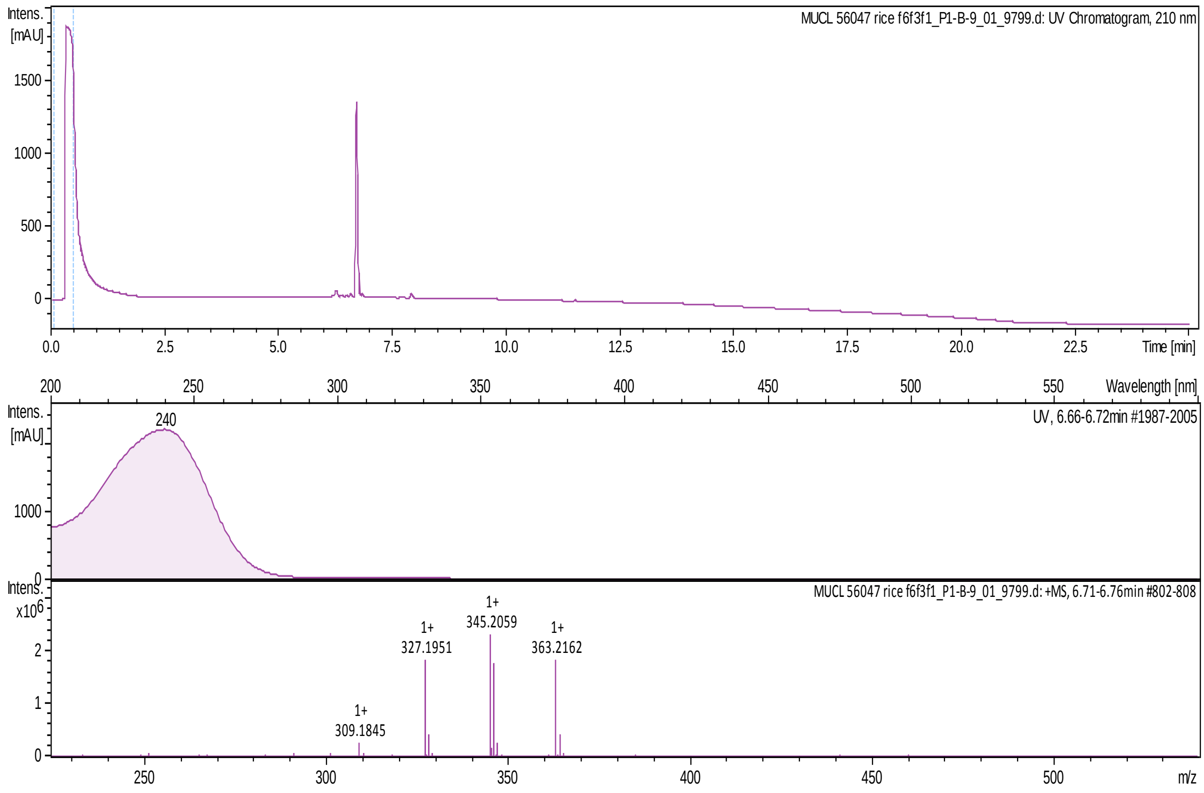

1 and 2D NMR data for Aethiopinolone C (3)

Figure 21: ^{1}H NMR spectrum of Aethiopinolone C (**3**) in acetone-d6 (500 MHz)

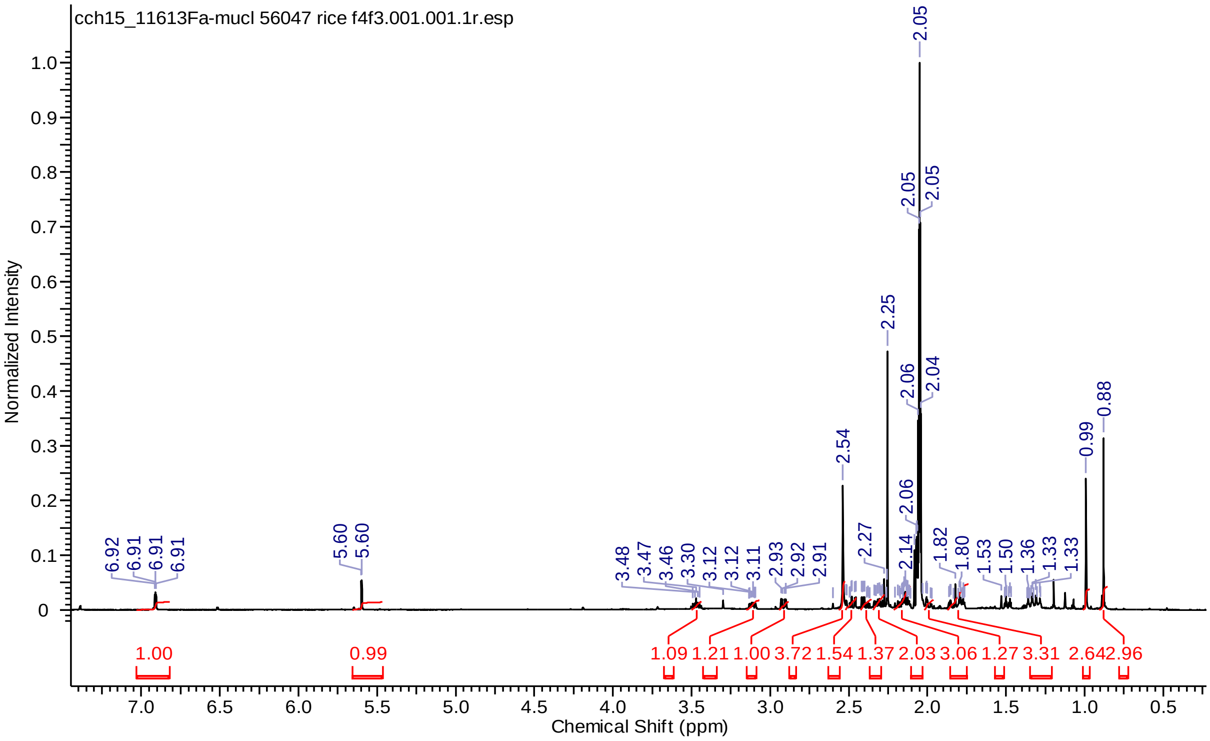

Figure 22: ^{13}C NMR spectrum of Aethiopinolone C (**3**) in acetone-d6 (125 MHz)

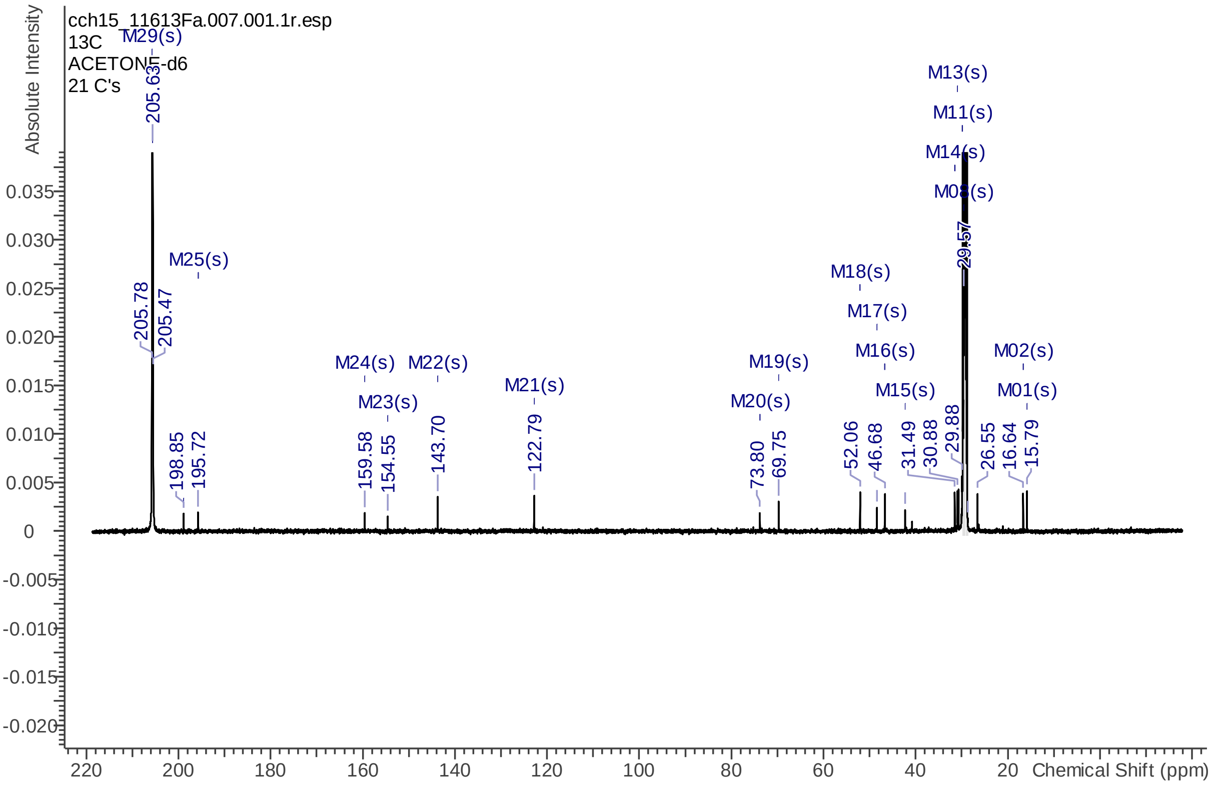

Figure 23: DEPT NMR spectrum of Aethiopinolone C (**3**) in acetone-d6 (125 MHz)

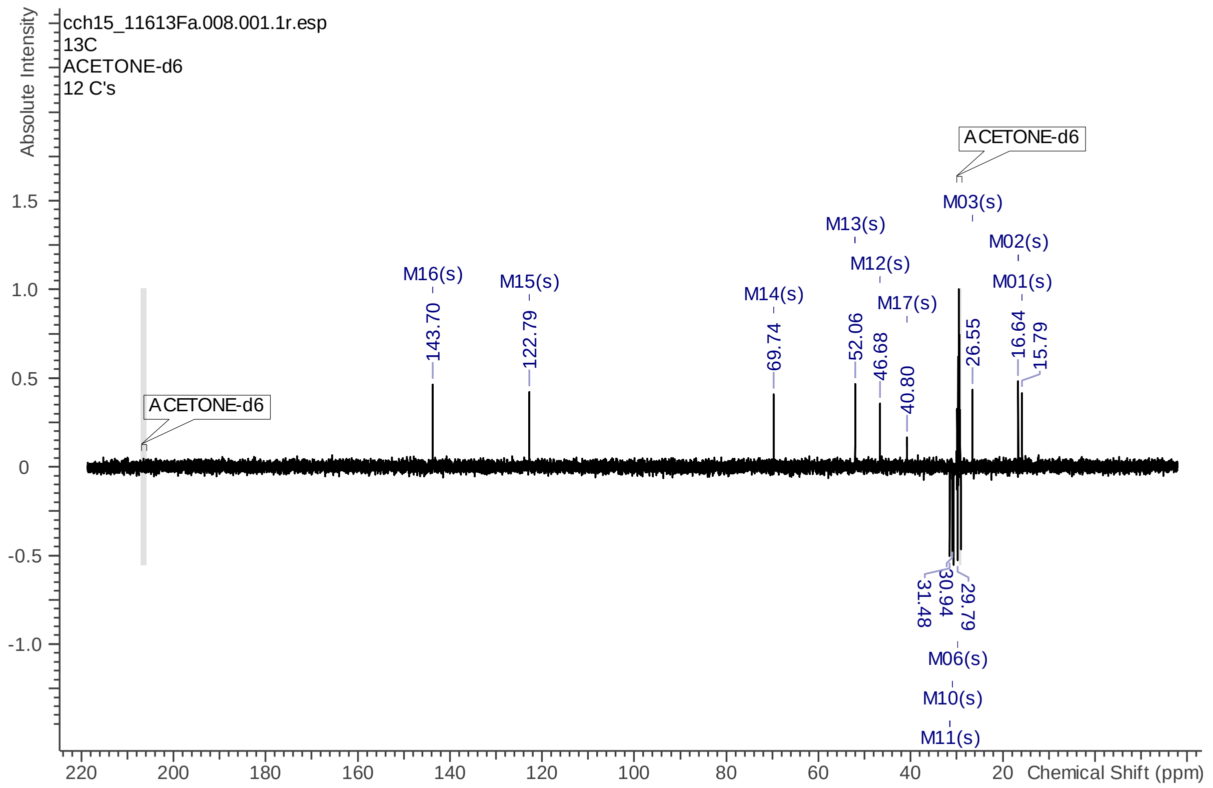

Figure 24: ^{1}H, ^{13}C HSQC spectrum of Aethiopinolone C (**3**) in acetone-d6 (500MHz, 125 MHz)

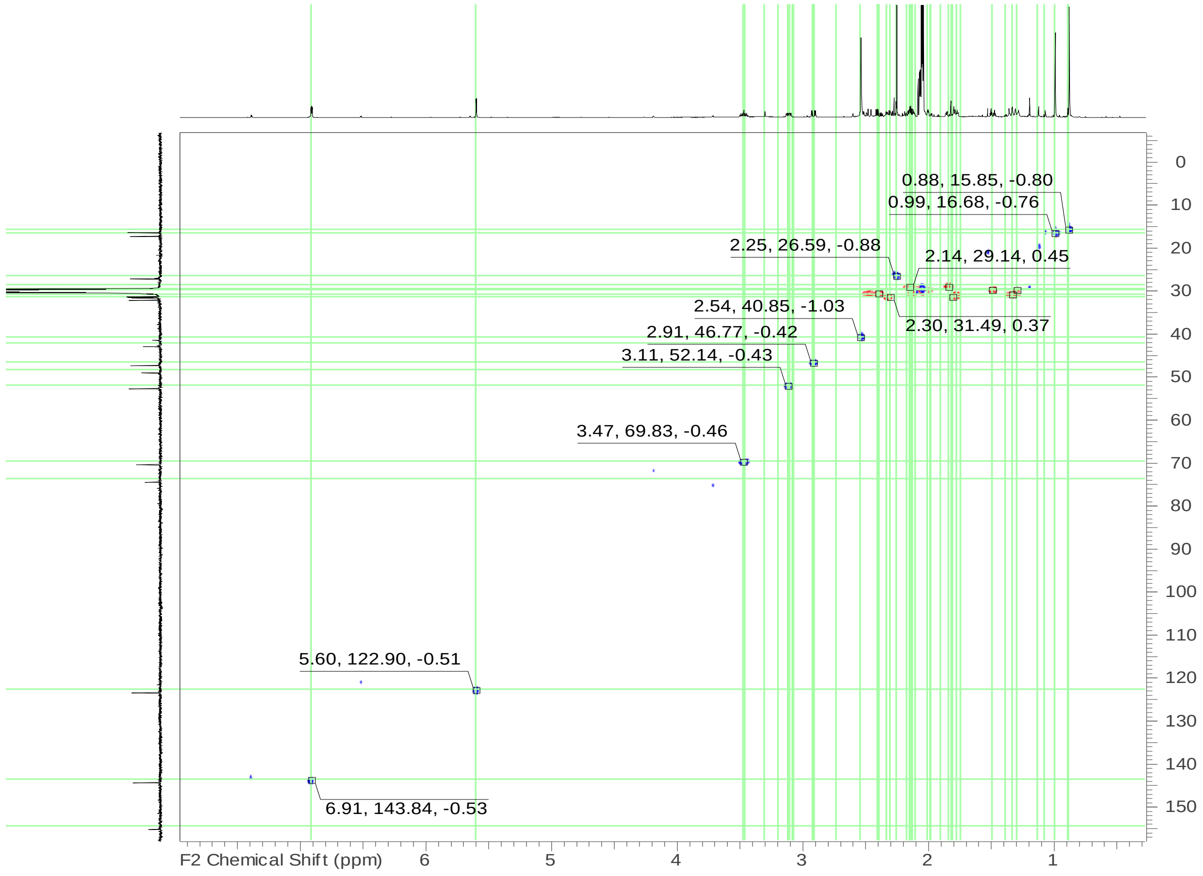

Figure 25: ^{1}H, ^{13}C HMBC spectrum of Aethiopinolone C (**3**) in acetone-d6 (500MHz, 125 MHz)

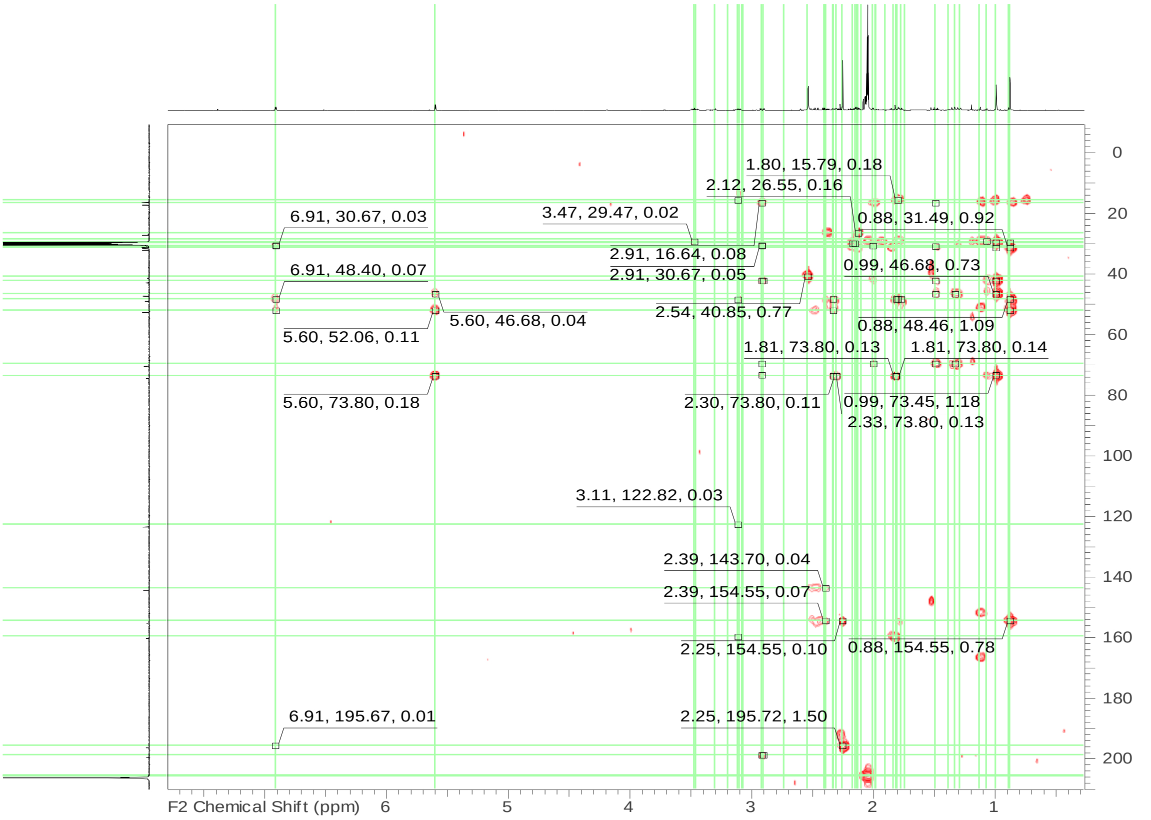

Figure 26: ^{1}H,^{1}H COSY spectrum of Aethiopinolone C (**3**) in acetone-d6 (500MHz)

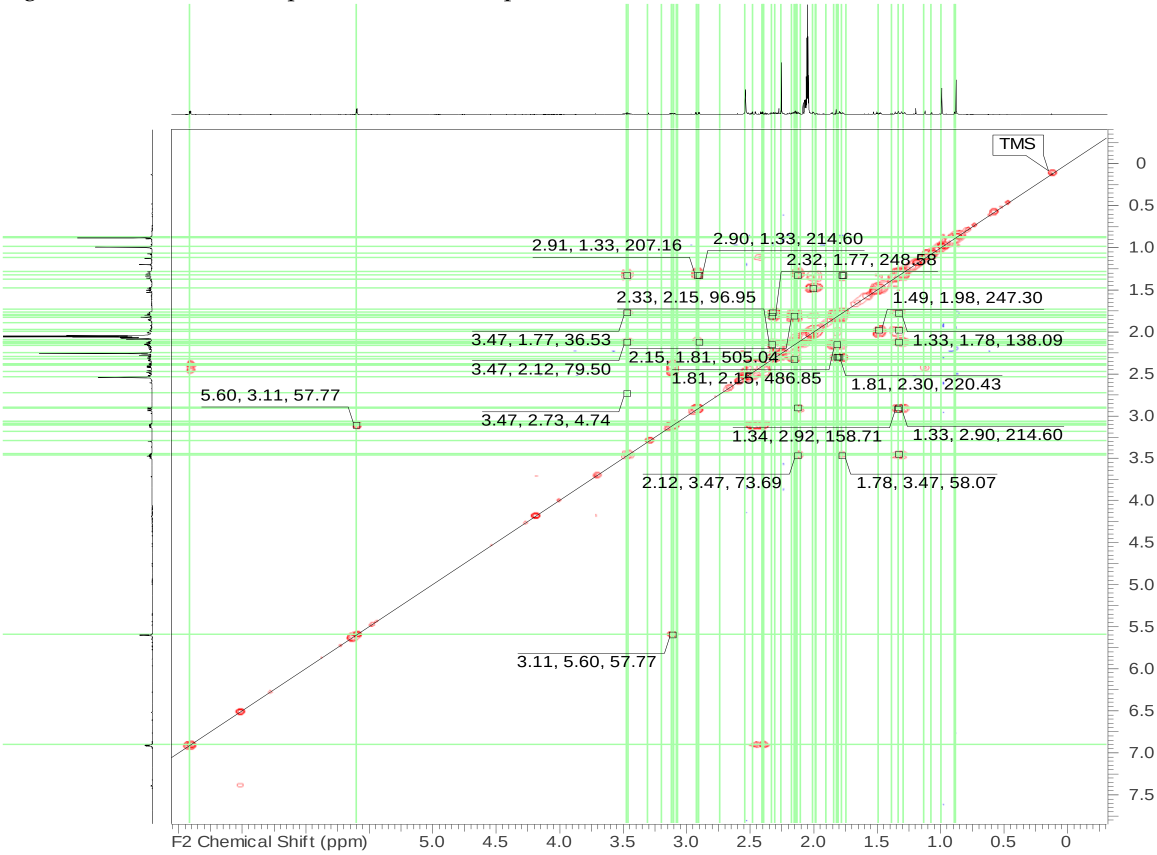

Figure 27: ^1H, ^1H ROESY spectrum of Aethiopinolone C (**3**) in acetone-d_6 (500MHz)

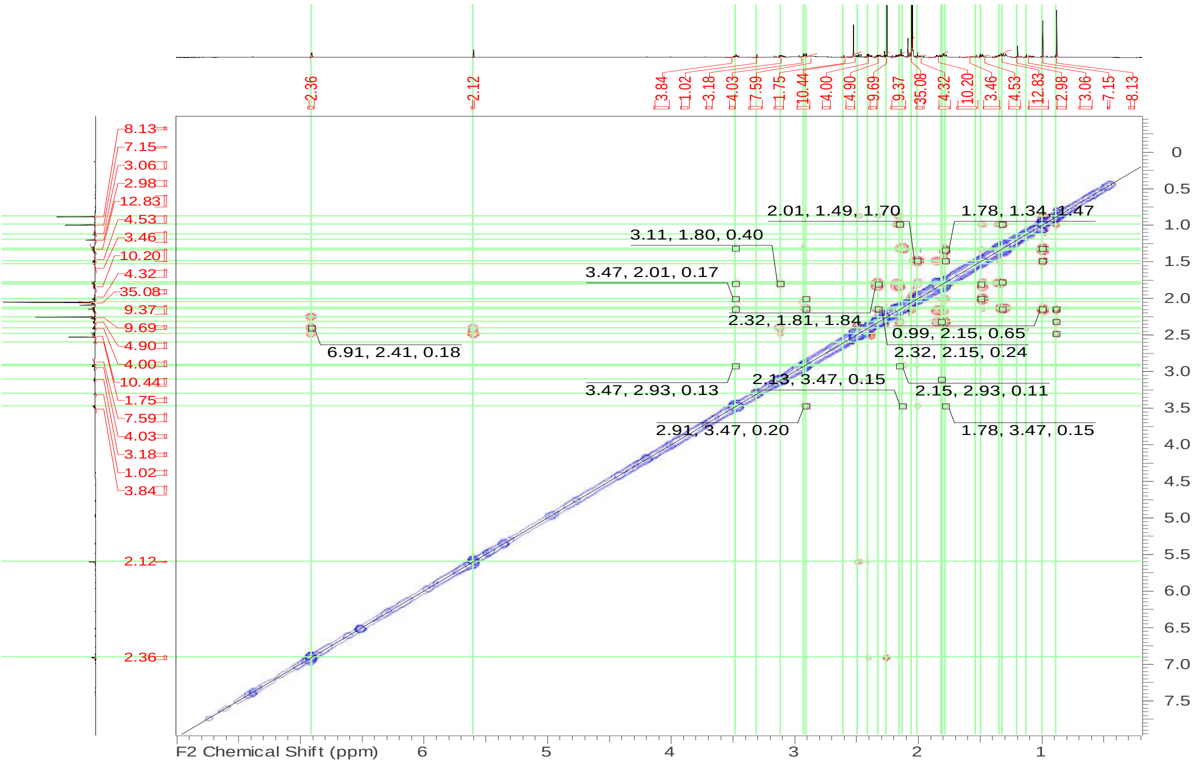

Figure 28: HRMS data for Aethiopinolone C (**3**)

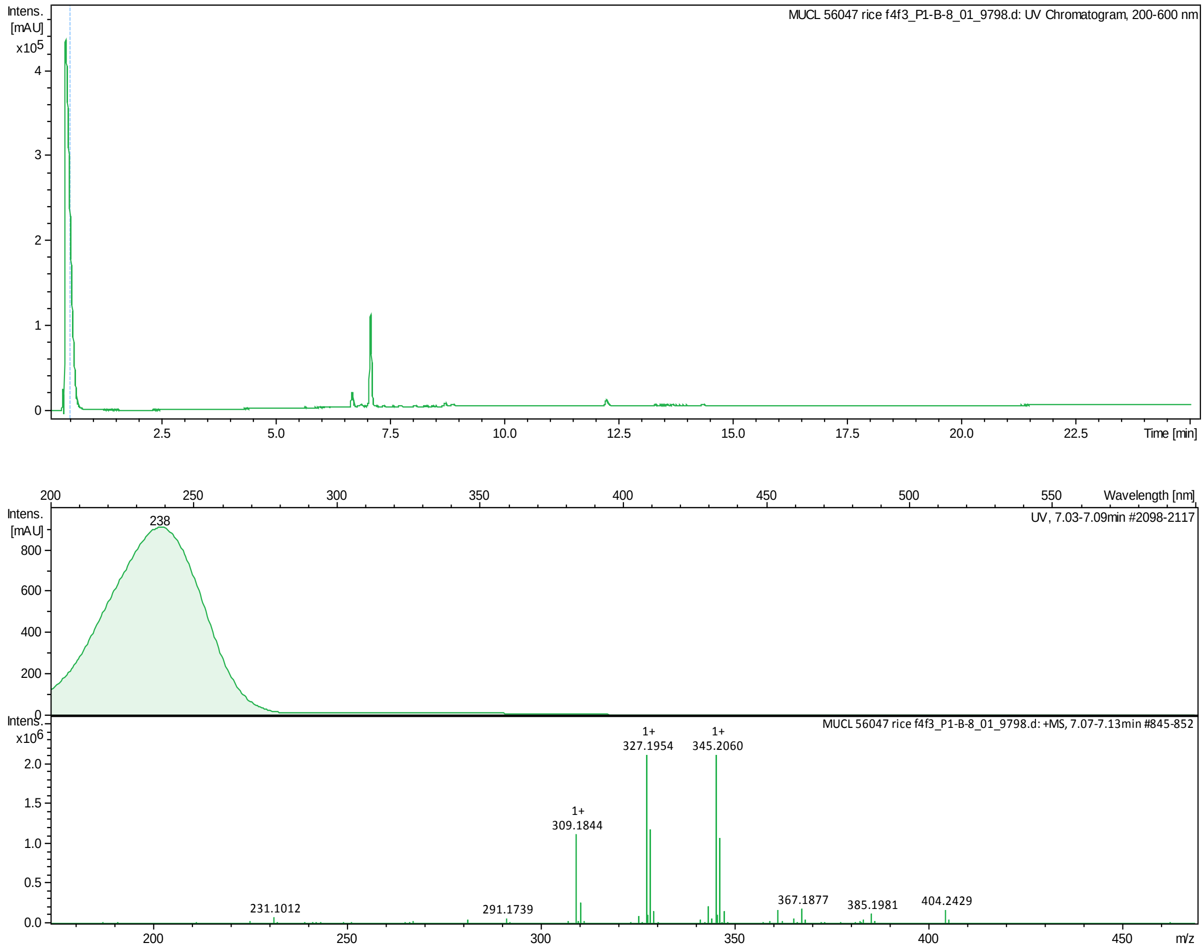

Figure 29: ^{1}H NMR spectrum of Aethiopinolone C (**3**) (*S*)- MTPA ester in pyridine –d_5 (700 MHz)

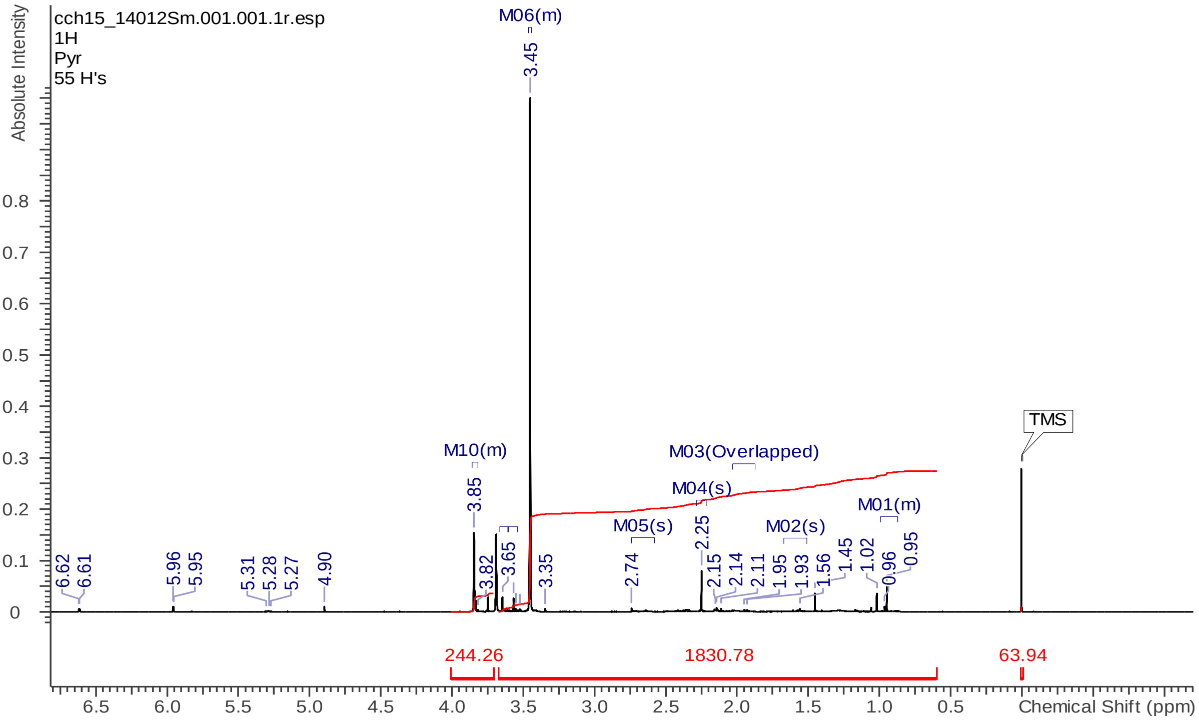

Figure 30: ^{1}H, ^{1}HCOSY spectrum of Aethiopinolone C (**3**) (S)- MTPA ester in pyridine –d_5 (700 MHz)

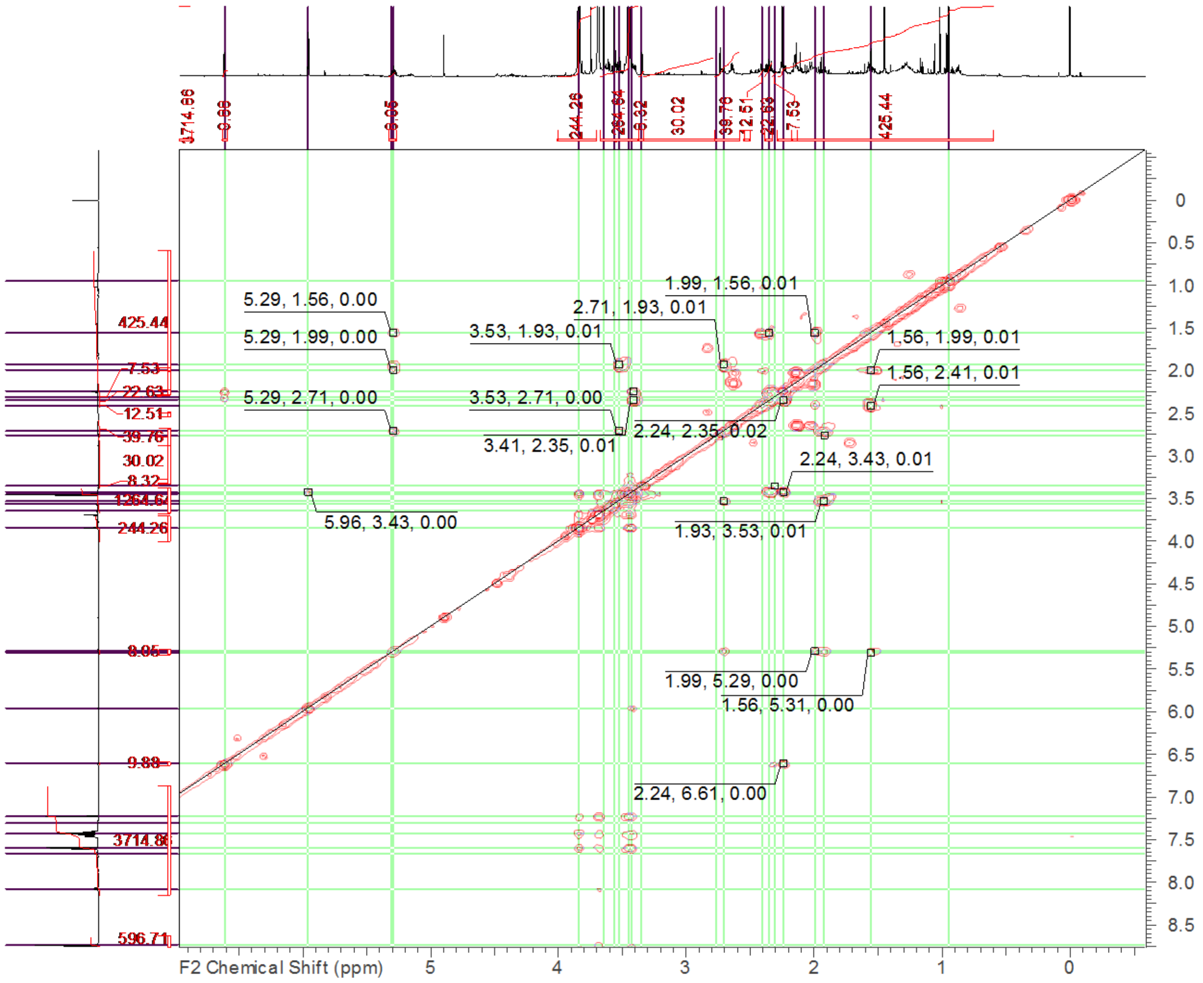

Figure 31:^{1}H NMR spectrum of Aethiopinolone C (**3**) (*R*)- MTPA ester in pyridine –d_5 (700 MHz)

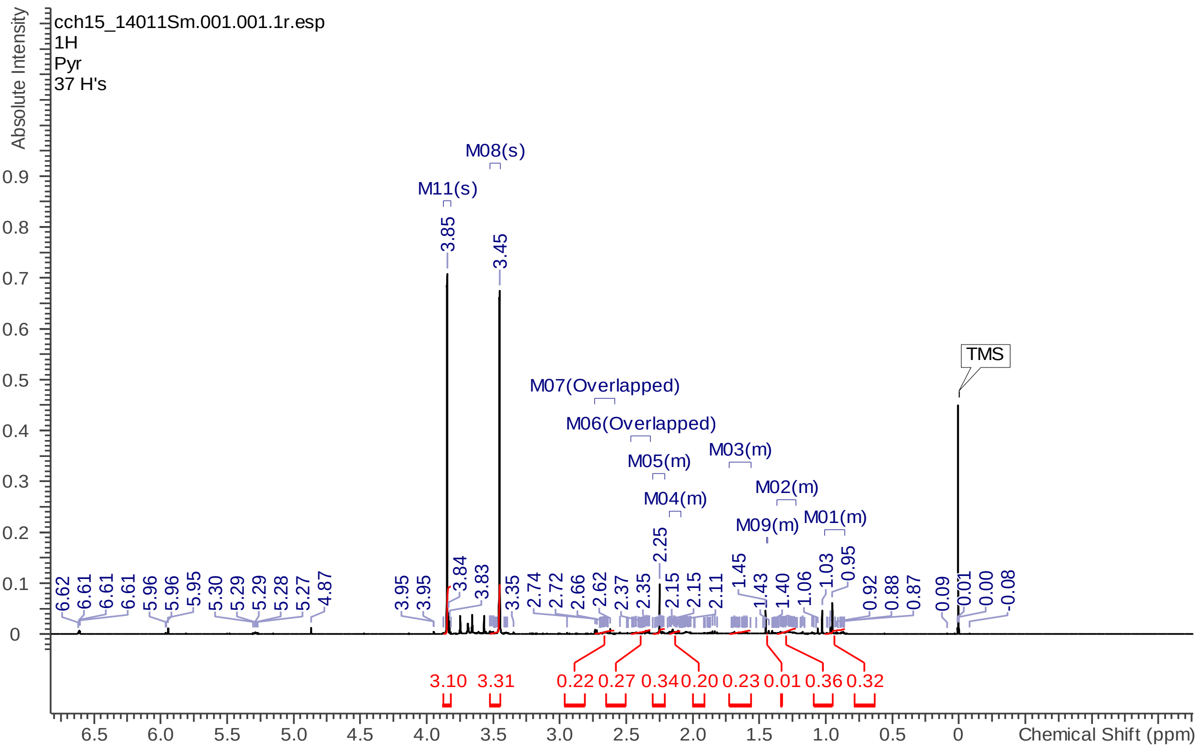

Figure 32:^{1}H, ^{1}HCOSY spectrum of Aethiopinolone C (**3**) (R)- MTPA ester in pyridine –d_5 (700 MHz)

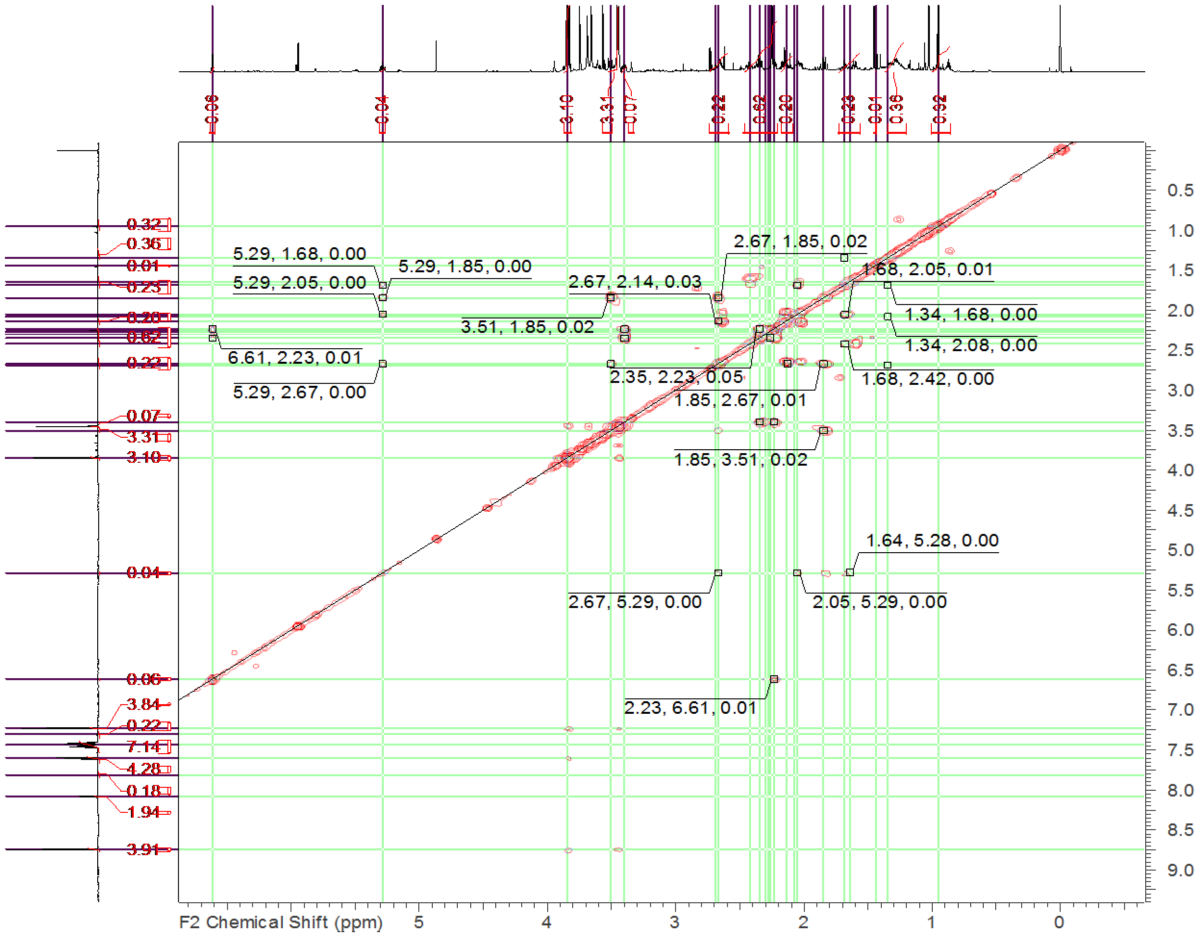

1 and 2D NMR data for Aethiopinolone D (4)

Figure 33:^{1}H NMR spectrum for Aethiopinolone D (**4**) in acetone-d₆ (175 MHz)

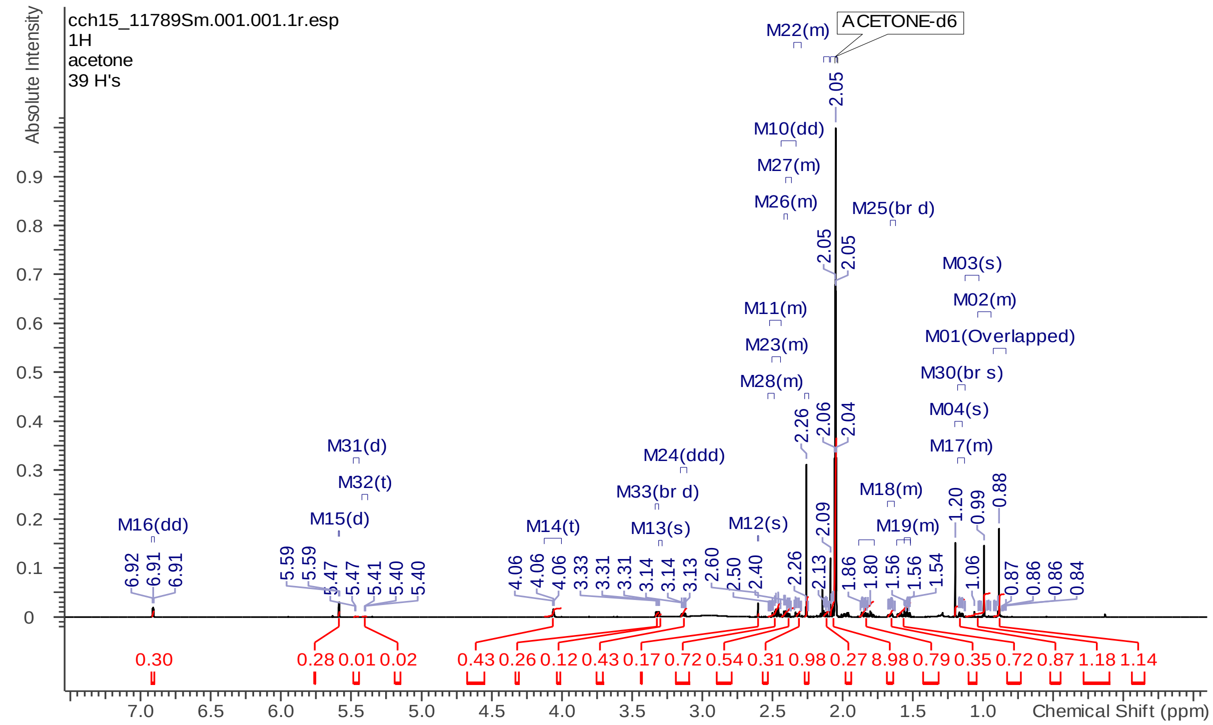

Figure 34: ^{13}C NMR spectrum of Aethiopinolone D (**4**) in acetone-d₆ (175 MHz)

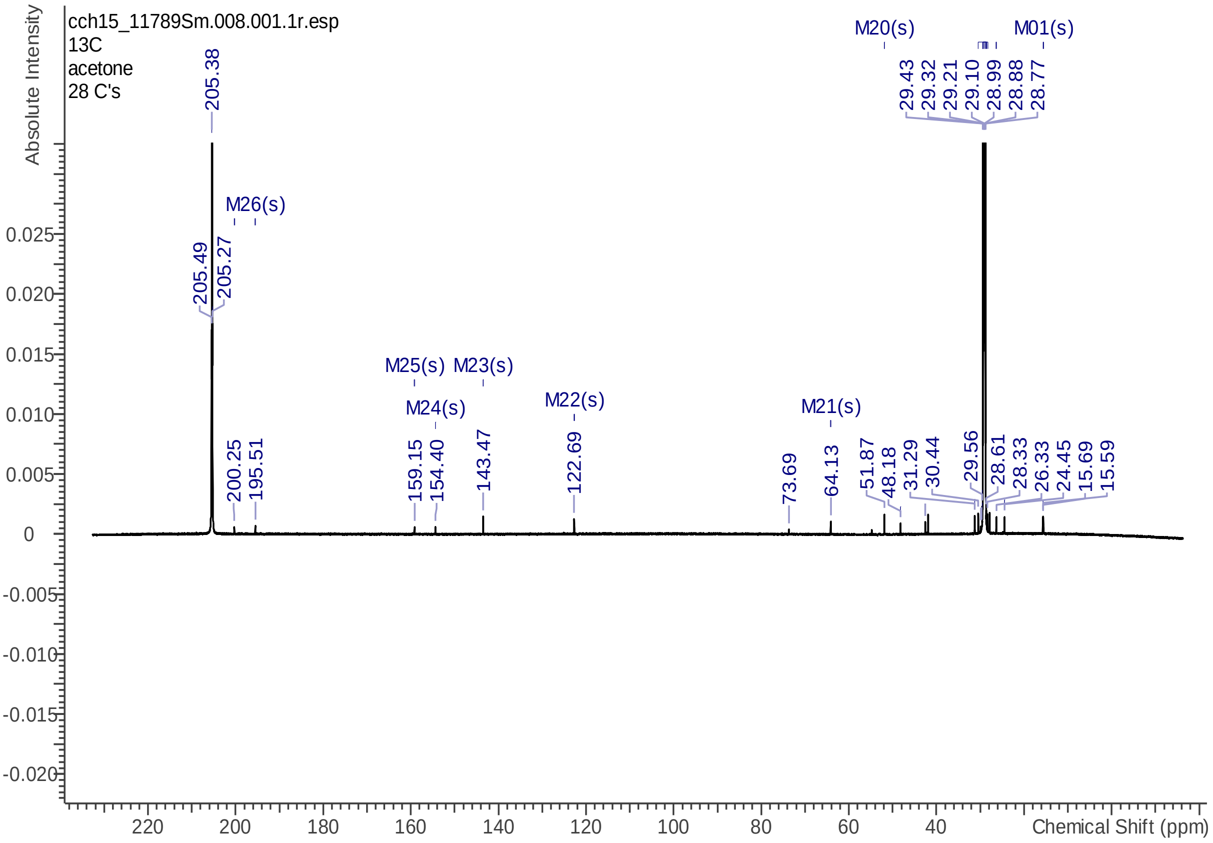

Figure 35: DEPT NMR spectrum of Aethiopinolone D (**4**) in acetone-d**6** (175 MHz)

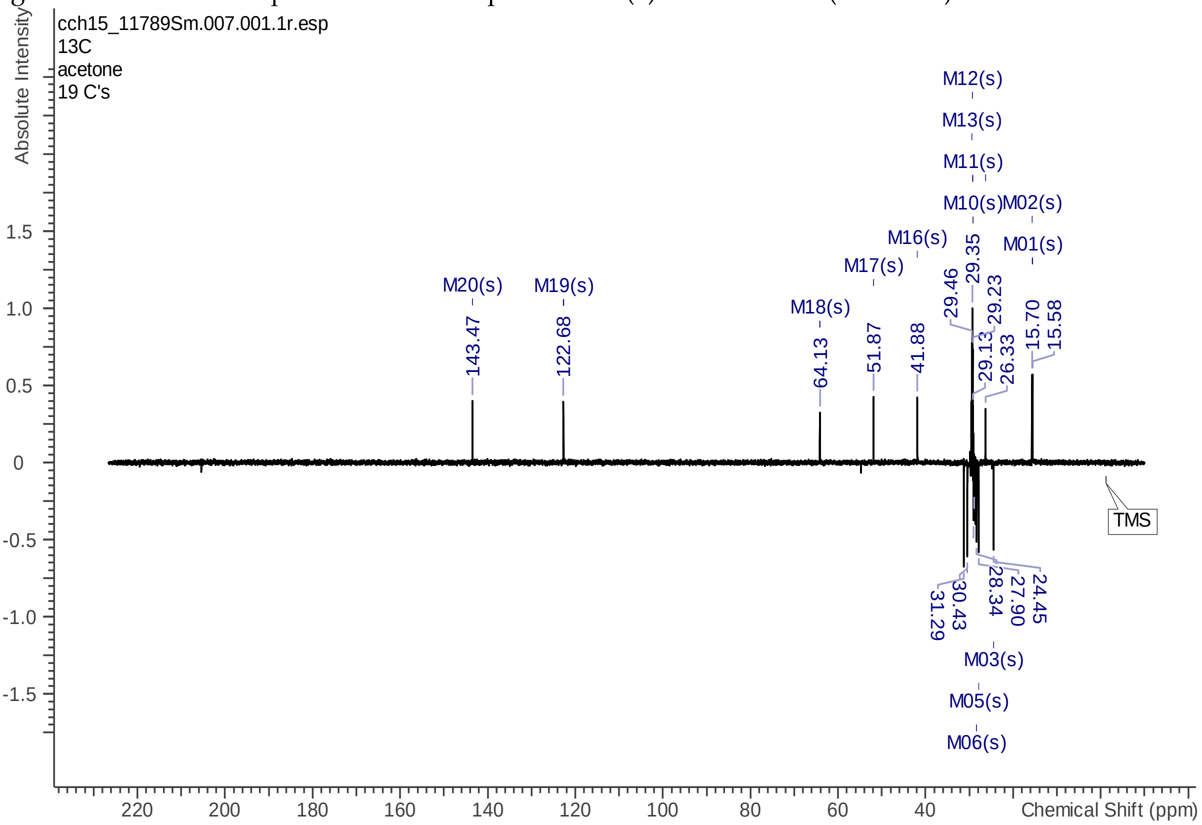

Figure 36: ^{1}H, ^{13}C HSQC spectrum of Aethiopinolone D (**4**) in acetone-d_6 (700 MHz, 175 MHz)

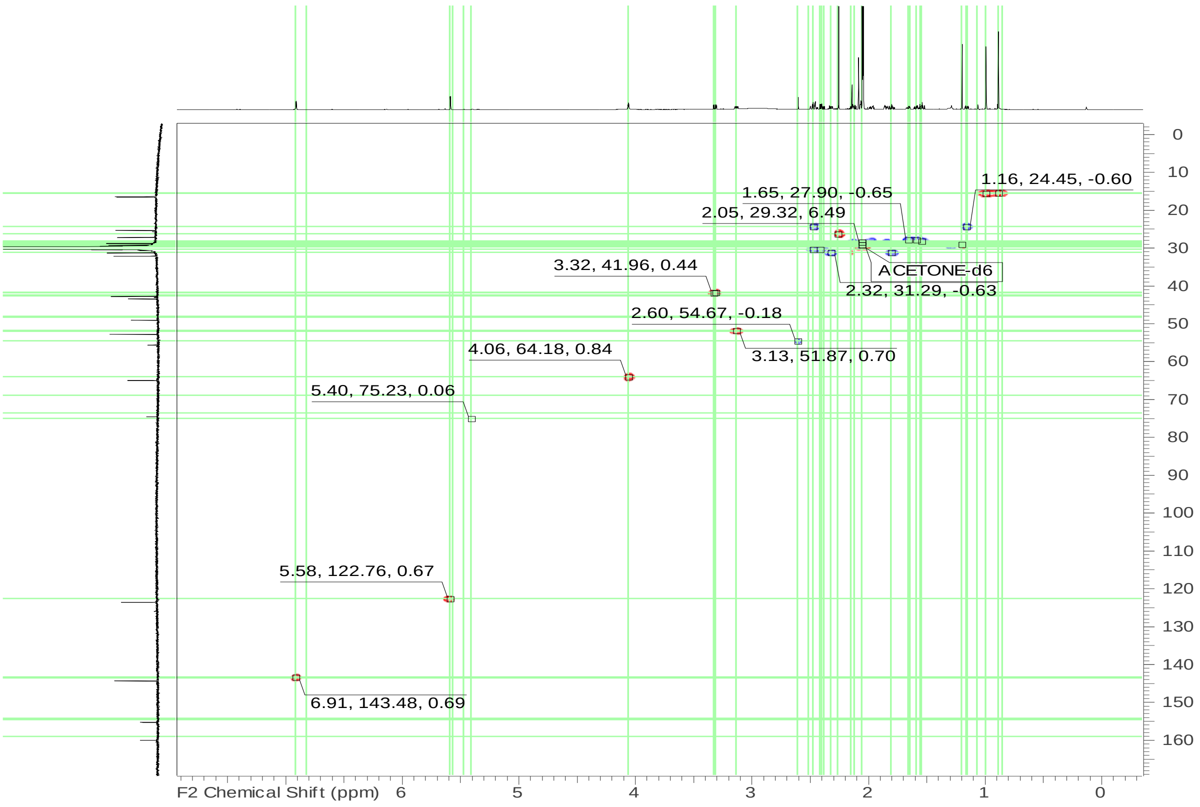

Figure 37:^{1}H,^{13}C HMBC spectrum of Aethiopinolone D (**4**) in acetone-d6 (700 MHz, 175 MHz)

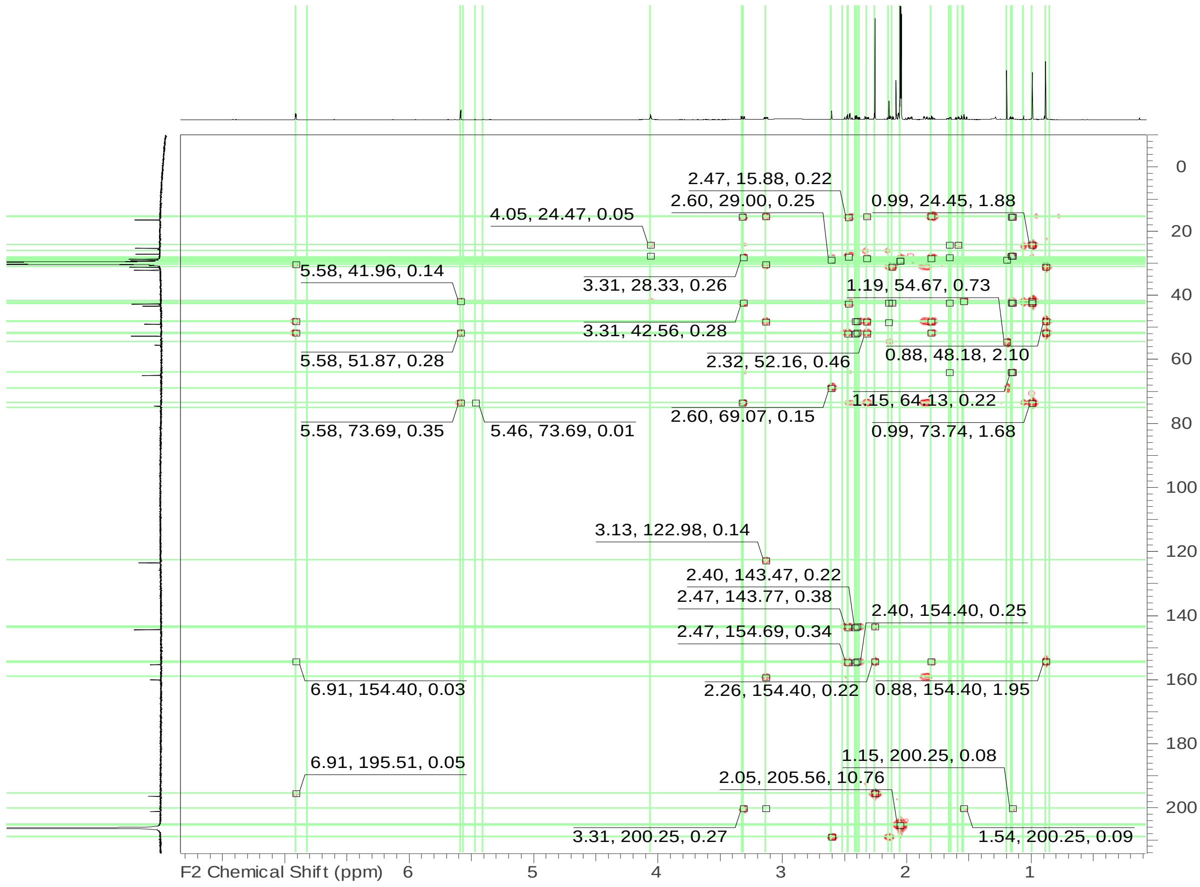

Figure 38:^{1}H, ^{1}H COSY spectrum of Aethiopinolone D (**4**) in acetone-d6 (700 MHz)

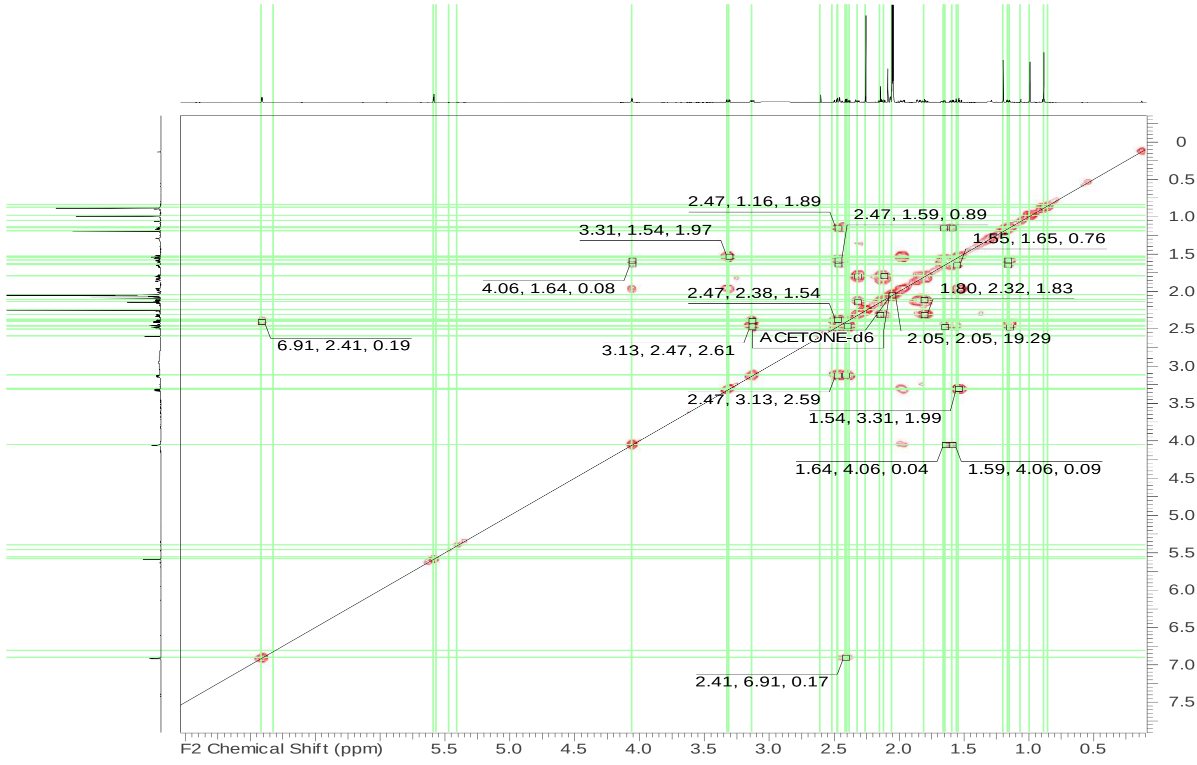

Figure 39: ^{1}H, ^{1}H ROESY spectrum of Aethiopinolone D (**4**) in acetone-d_6 (700 MHz)

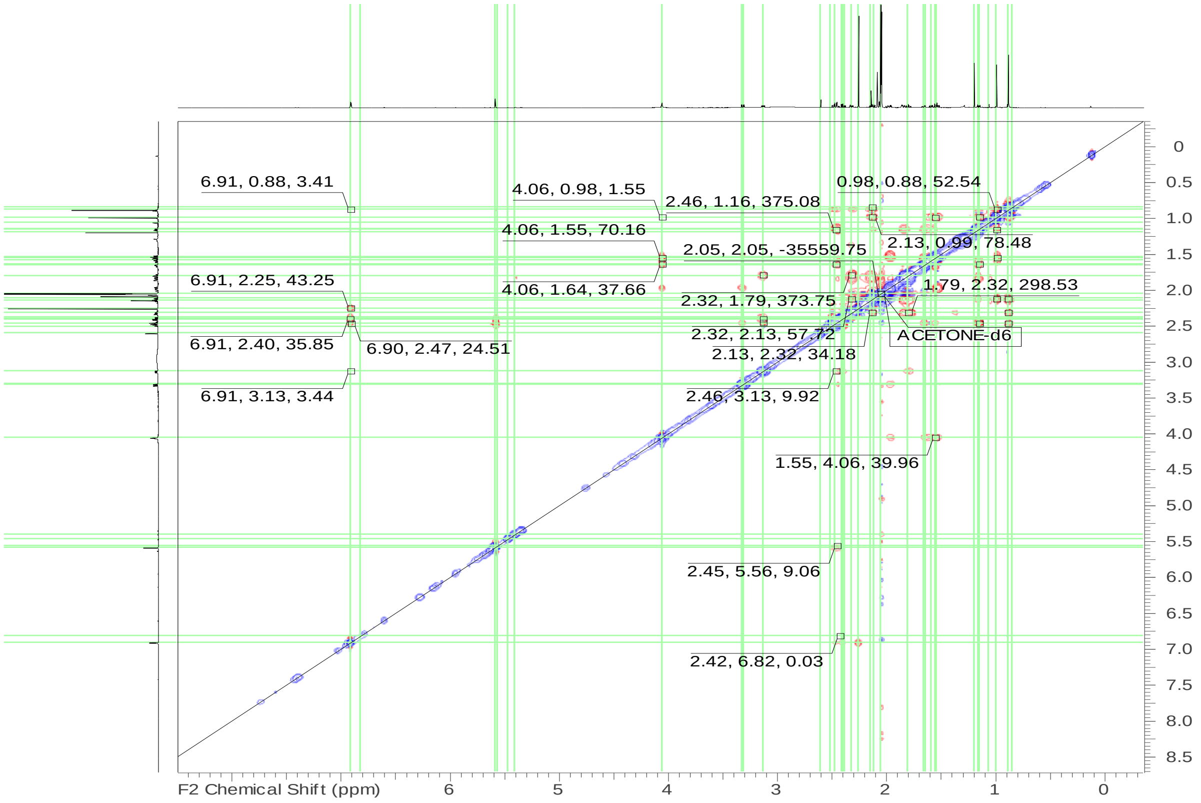

Figure 40: HRMS spectrum for Aethiopinolone D (**4**)

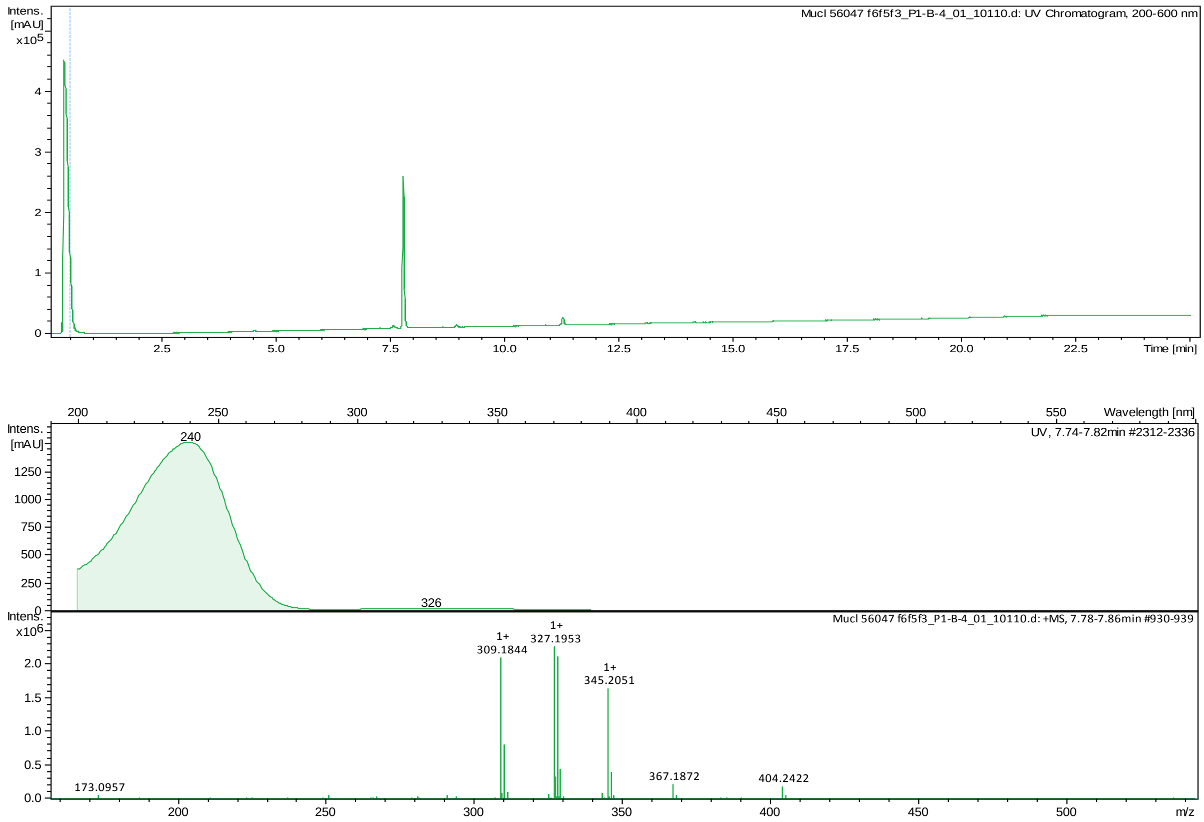

Figure 41:^{1}H NMR spectrum of Aethiopinolone D (**4**) (*S*)- MTPA ester in pyridine –d_5 (700 MHz)

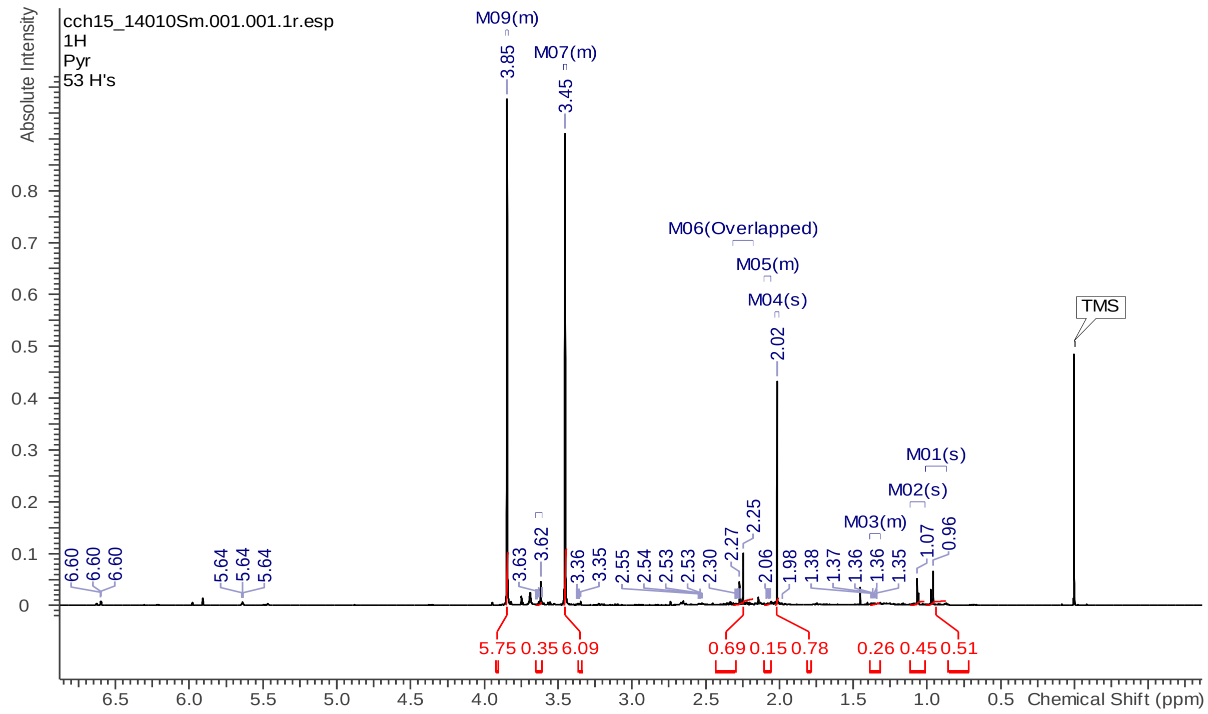

Figure 42:^{1}H, ^{1}H COSY spectrum of Aethiopinolone D (**4**) (*S*)- MTPA ester in pyridine –d_5 (700 MHz)

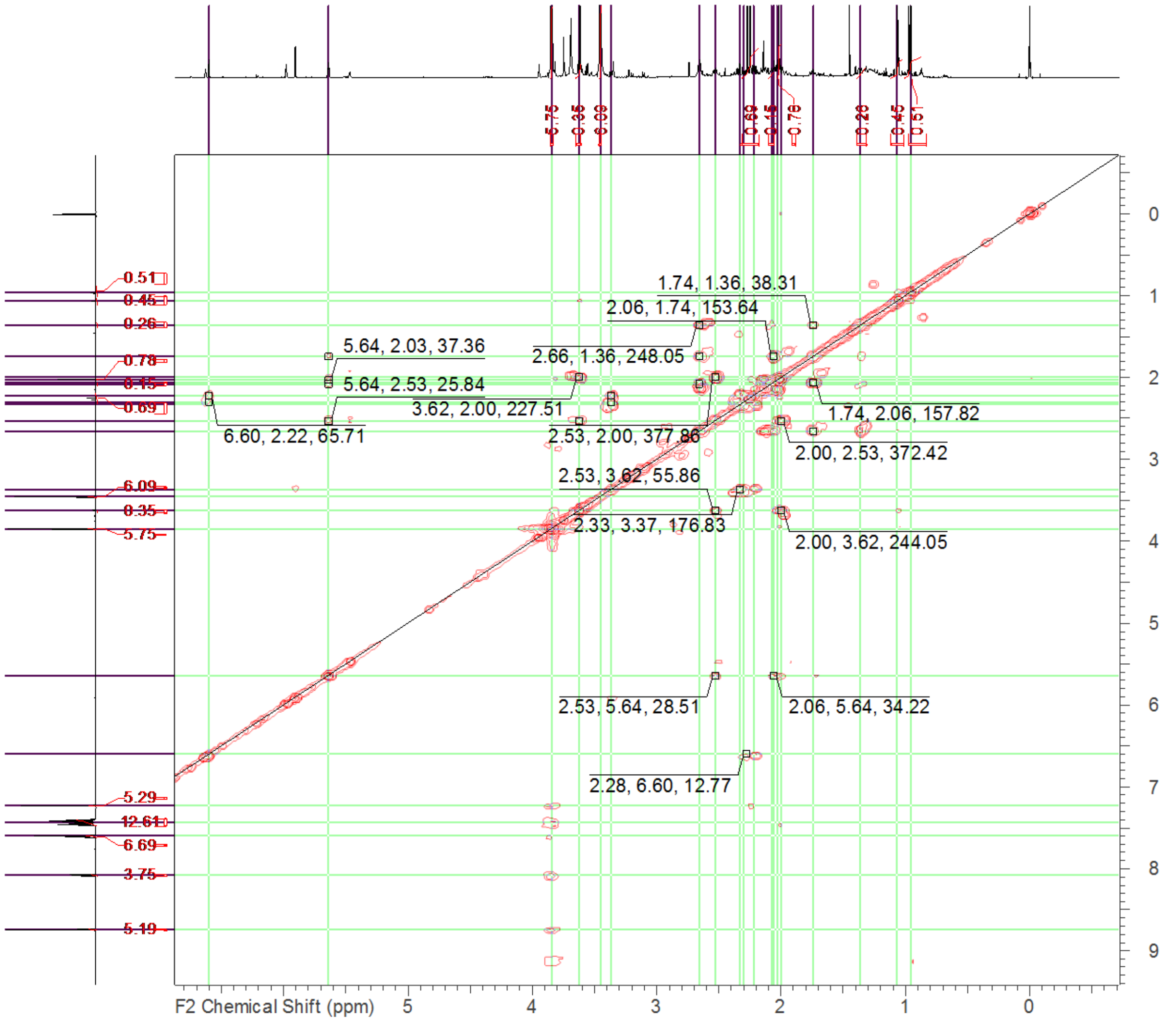

Figure 43:¹H NMR spectrum of Aethiopinolone D (**4**) (R)- MTPA ester in pyridine –d₅ (700 MHz)

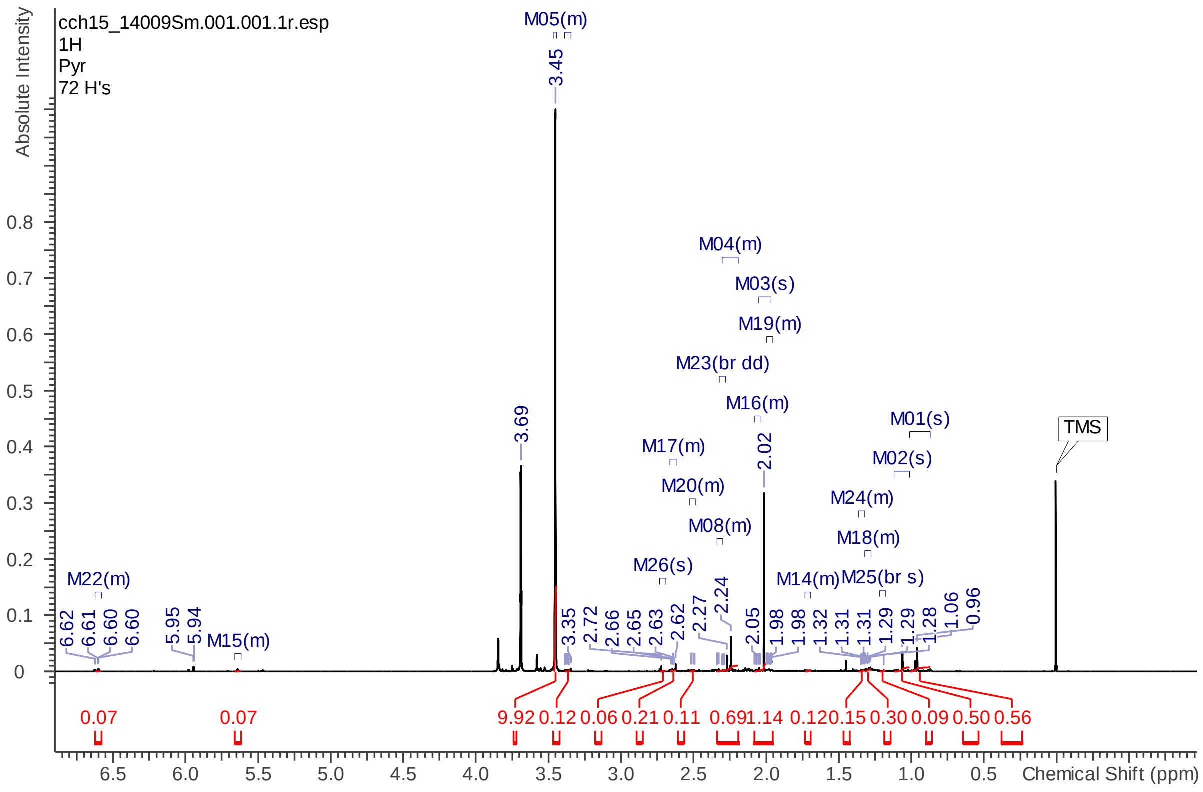

Figure 44:¹H, ¹H COSY spectrum of Aethiopinolone D (**4**) (*R*)- MTPA ester in pyridine –d₅ (700 MHz)

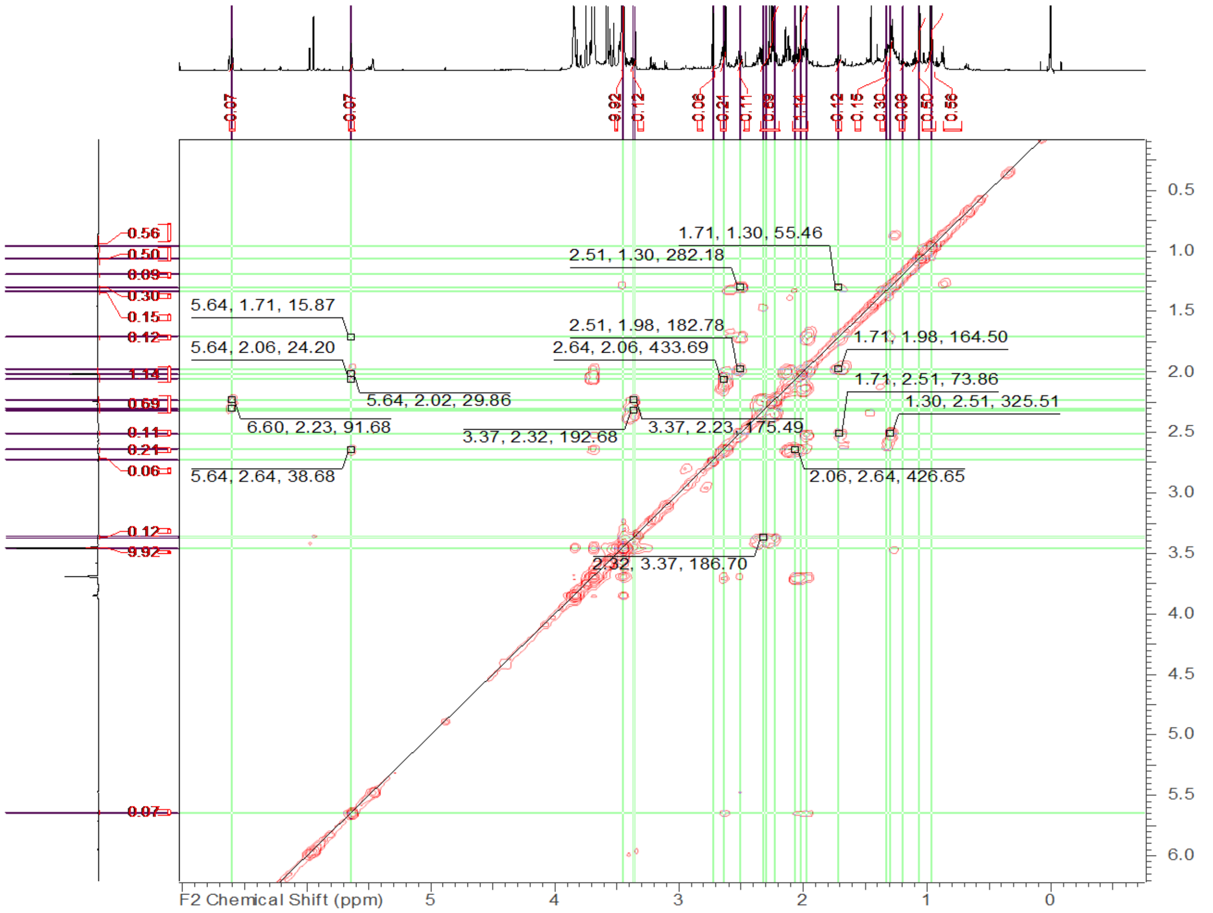

1 and 2D NMR data for Aethiopinolone E (5)

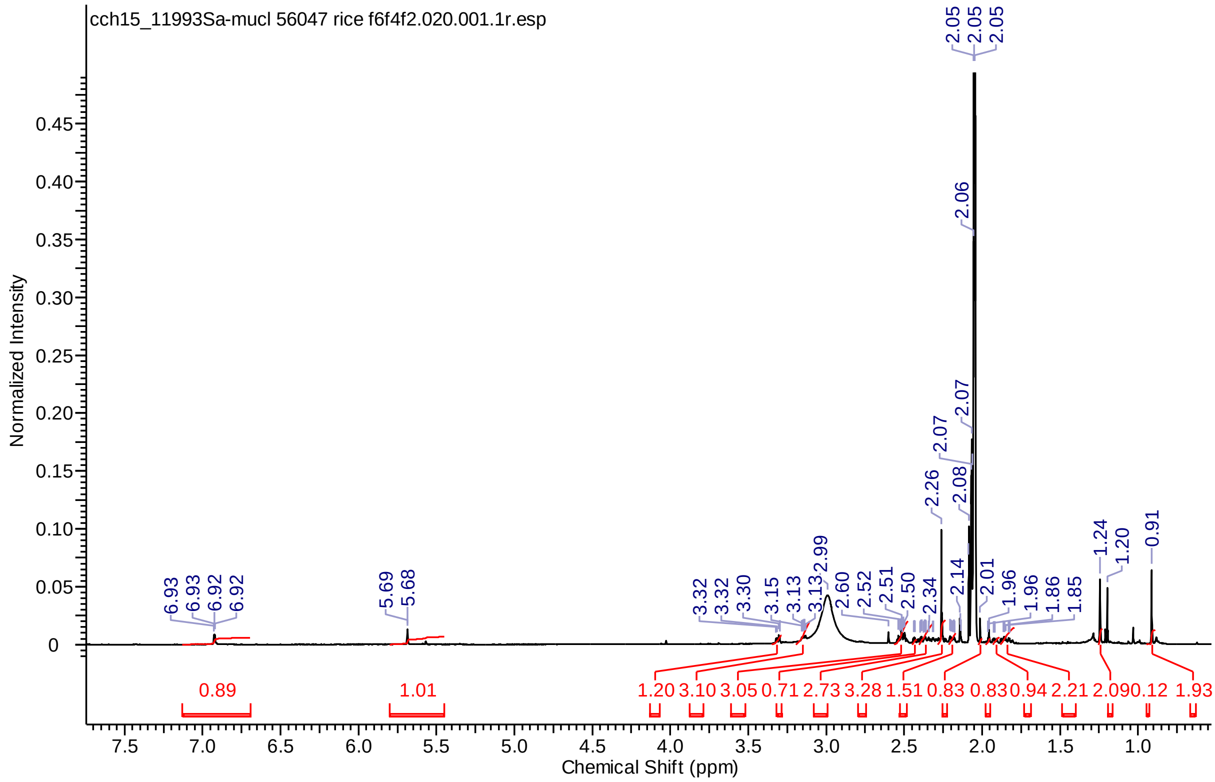

Figure 45:^{1}H NMR spectrum of Aethiopinolone E (**5**) in acetone-d6 (700 MHz)

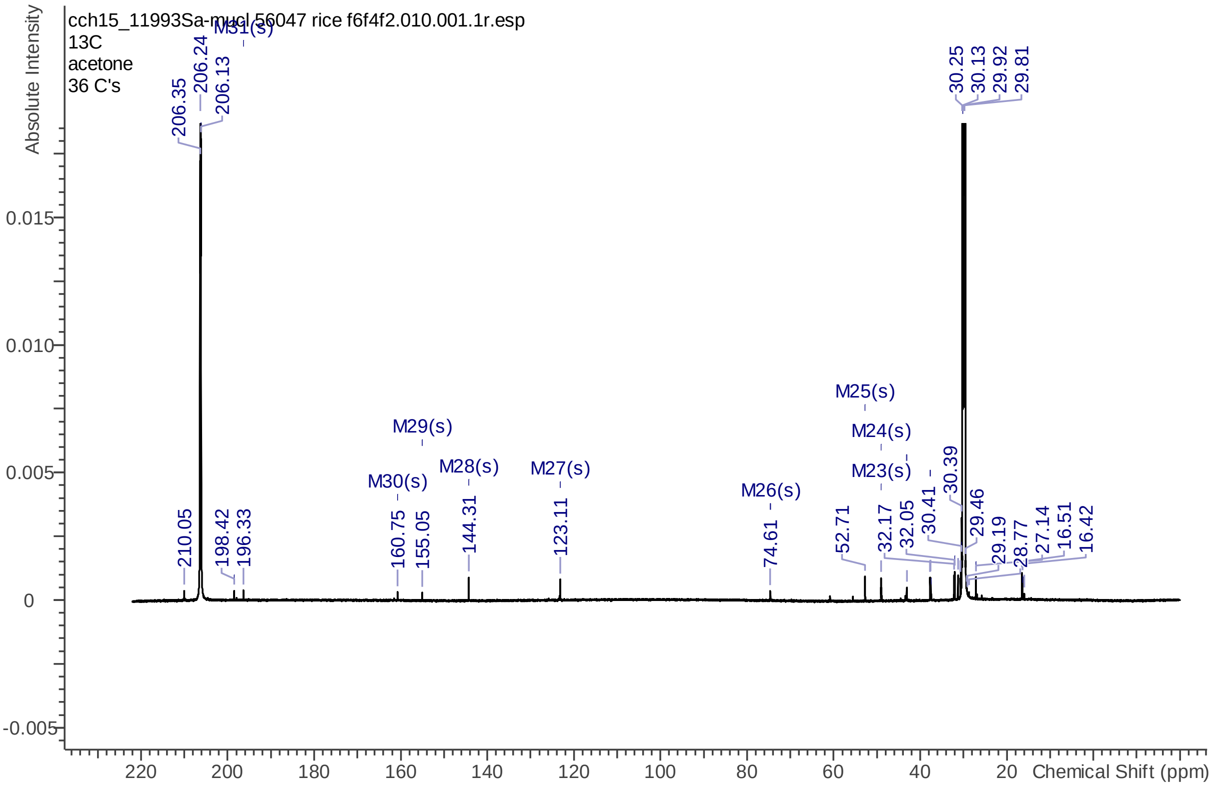

Figure 46:^{13}C NMR spectrum of Aethiopinolone E (**5**) in acetone-d6 (175 MHz)

Figure 47: DEPT NMR spectrum of Aethiopinolone E (**5**) in acetone-d6 (175 MHz)

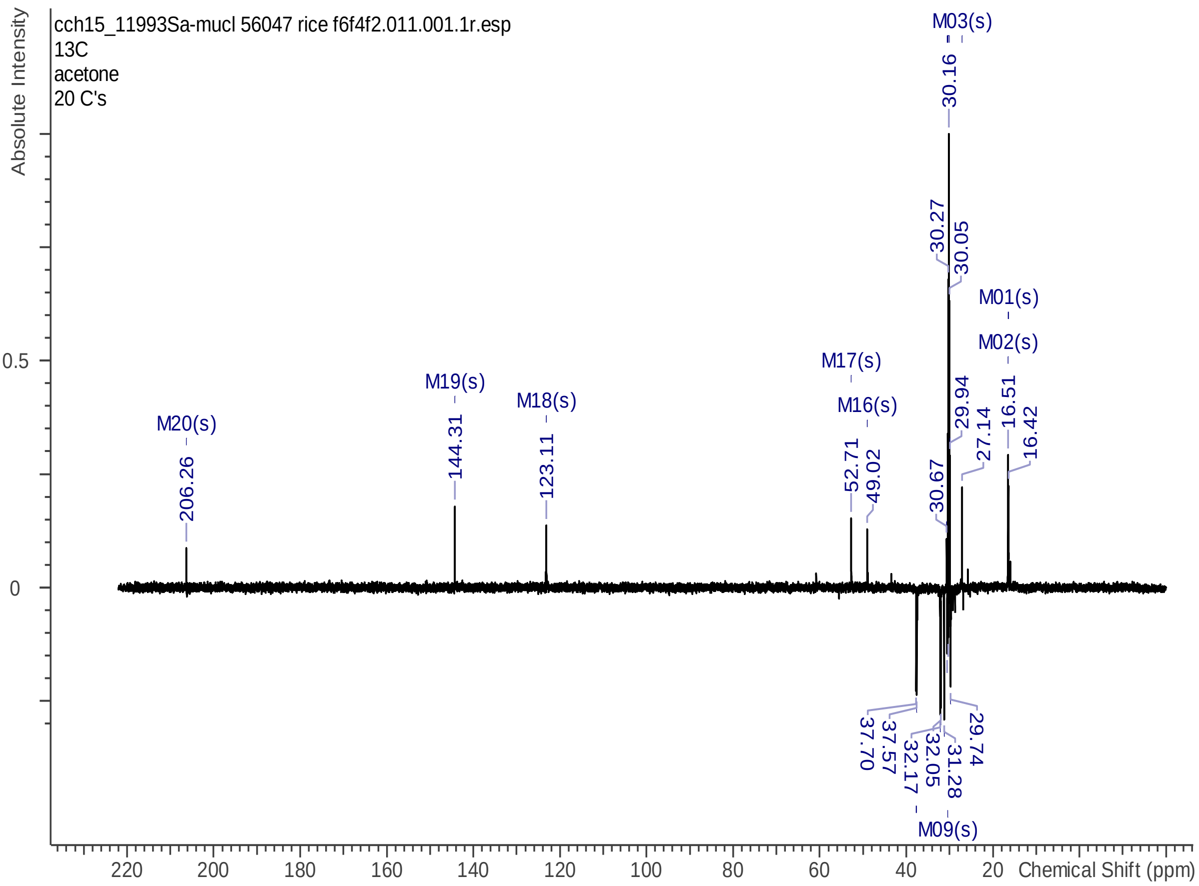

Figure 48:^{1}H, ^{13}C HSQC NMR spectrum of Aethiopinolone E (**5**) in acetone-d6 (700 MHz, 175 MHz)

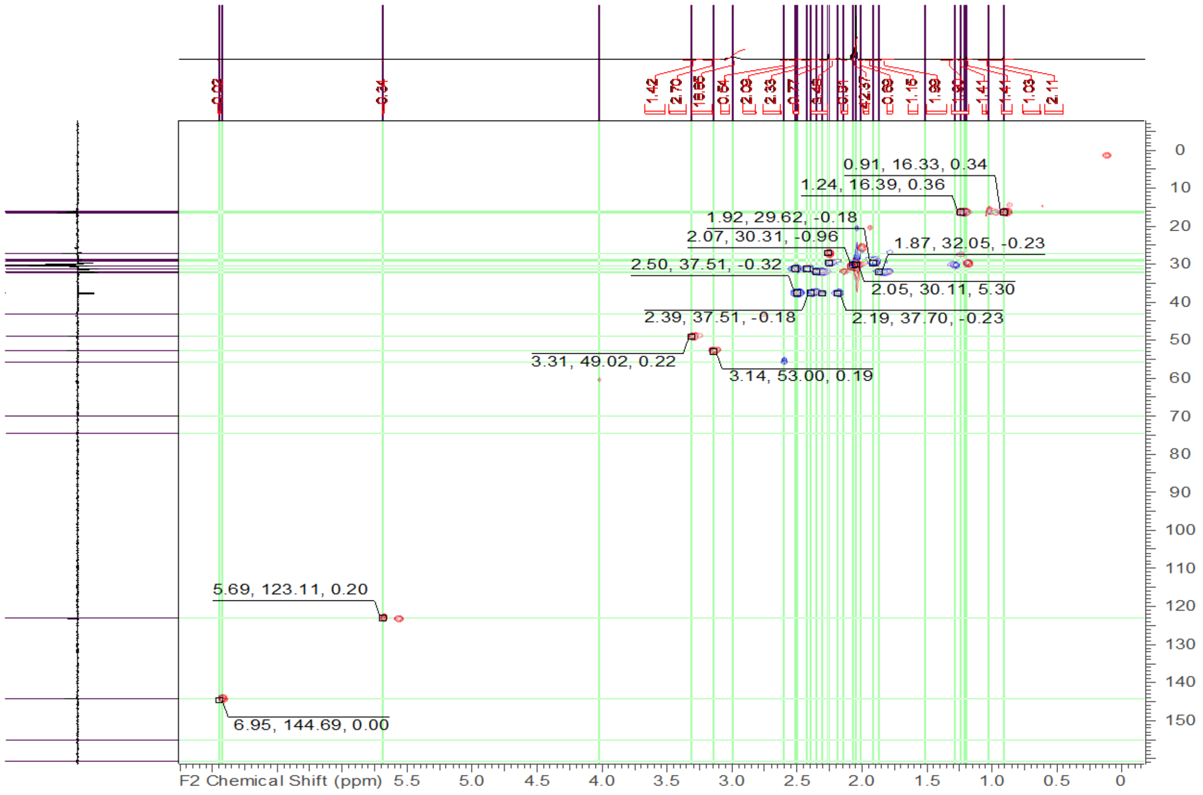

Figure 49:¹H, ¹³C HMBC NMR spectrum of Aethiopinolone E (5) in acetone-d₆ (700 MHz, 175 MHz)

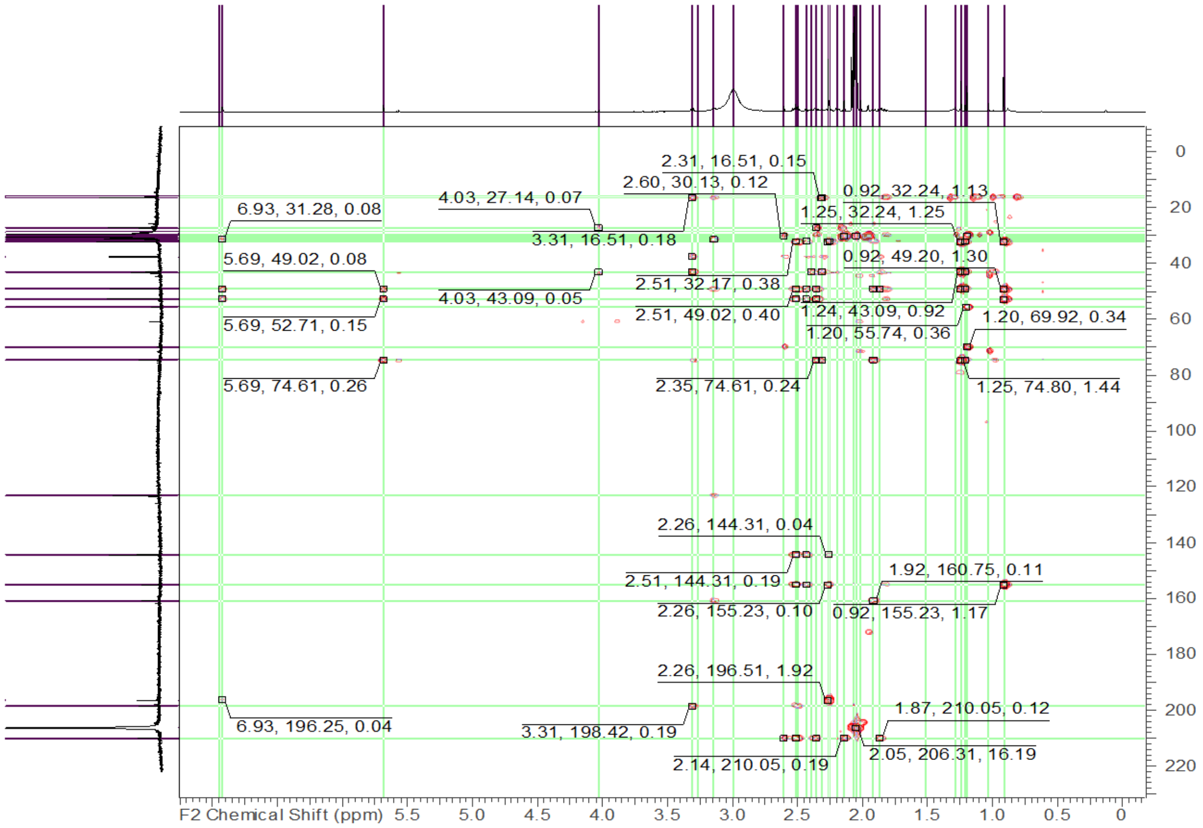

Figure 50:¹H, ¹H COSY NMR spectrum of Aethiopinolone E (5) in acetone-d₆ (700 MHz)

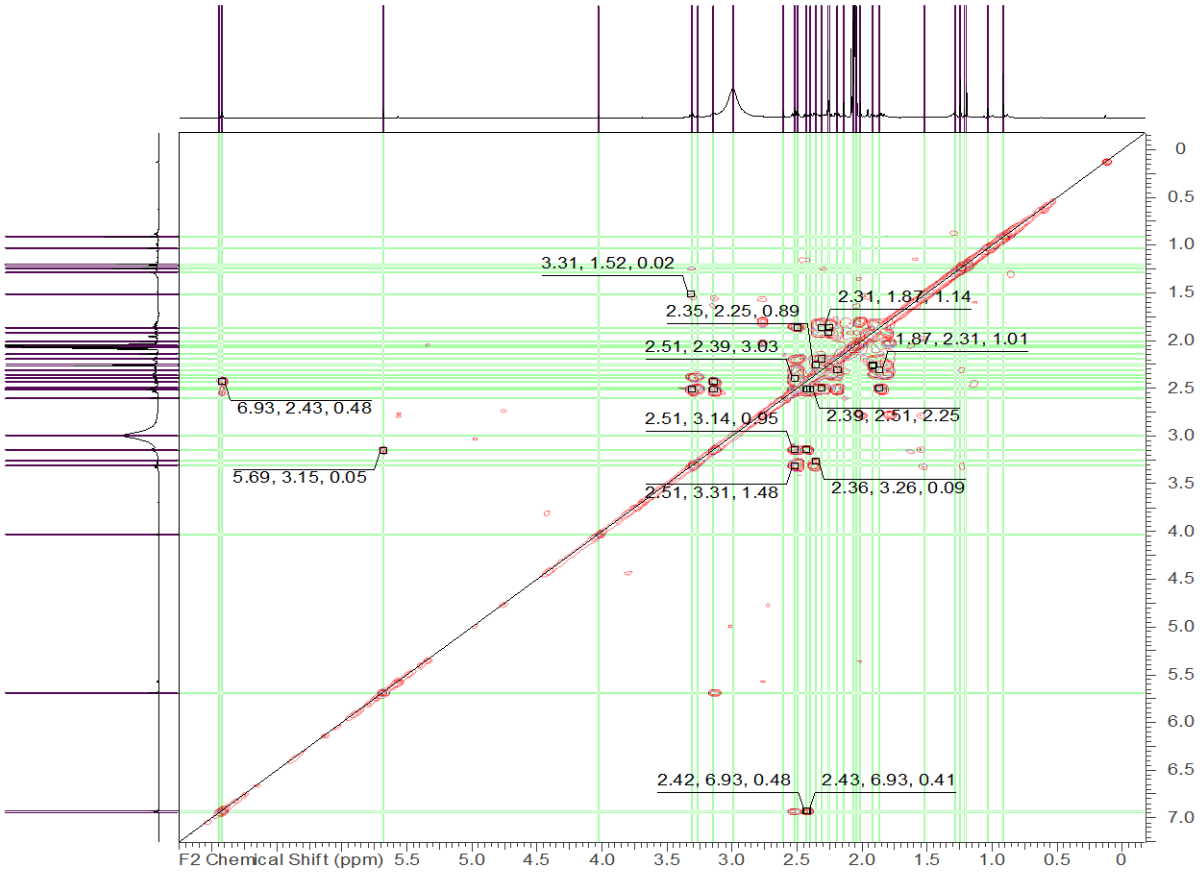

Figure 51: ^{1}H, ^{1}H ROESY NMR spectrum of Aethiopinolone E (**5**) in acetone-d_6 (700 MHz)

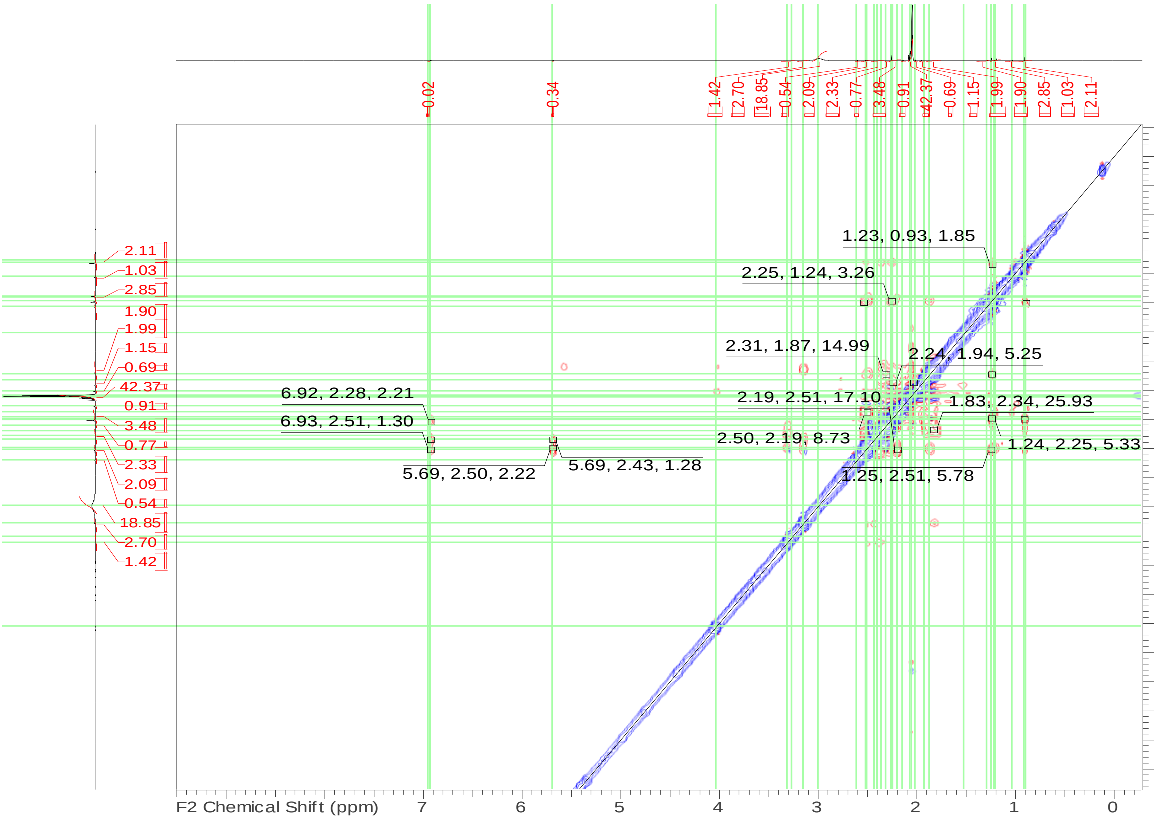

Figure 52: HRMS spectrum for Aethiopinolone **E** (**5**)

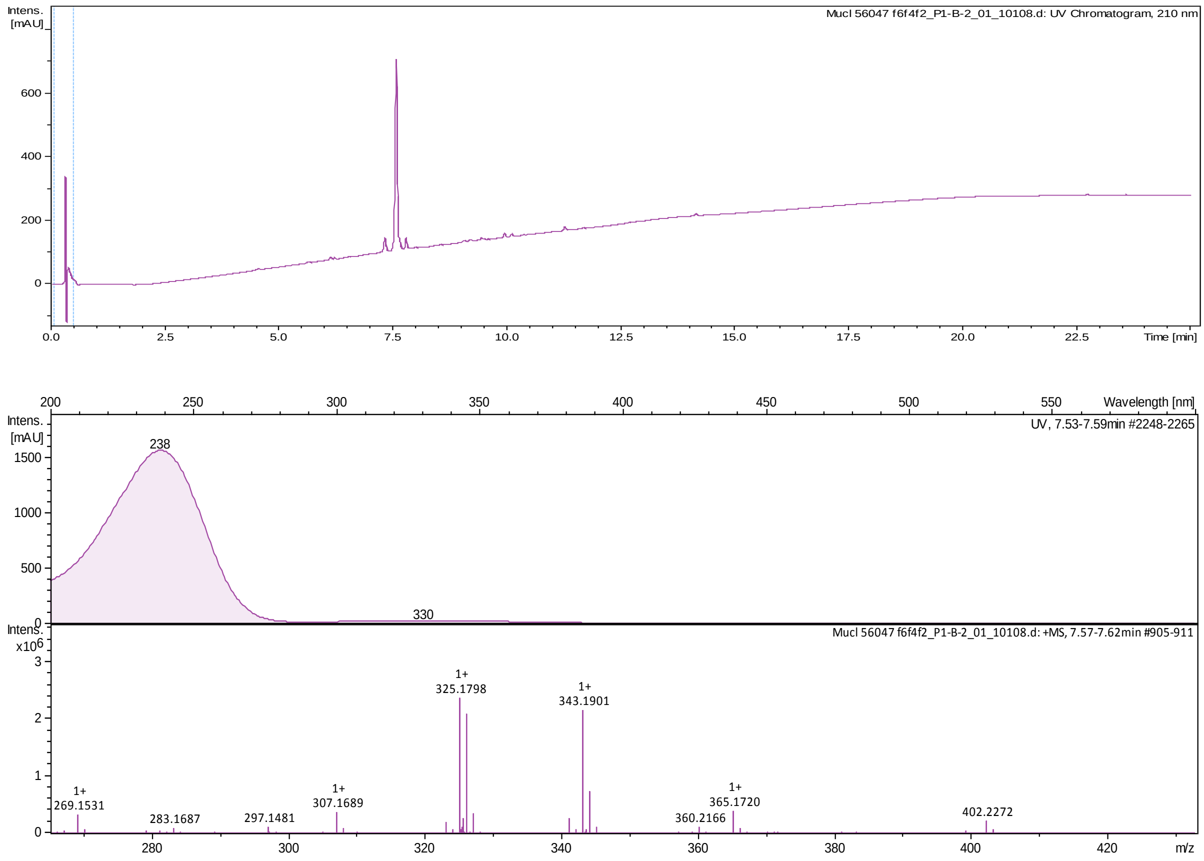

Table 1.

Table 1: *S*-MTPA ester and *R*-MTPA ester chemical shifts analysis for Aethiopinolone A (**1**)

	S	R	S-R
4α<br>4β	2.388<br>1.631	2.309<br>1.590	+0.079<br>+0.041
5	2.985	2.970	+0.015
7	5.766	5.757	+0.009
14	3.040	3.038	+0.002
17	2.470	2.468	+0.002
3	5.016	5.012	+0.004
2α<br>2β	1.945<br>1.515	2.023<br>1.625	-0.078<br>-0.110
1α<br>1β	2.011<br>1.303	2.334<br>1.364	-0.016<br>-0.061
19	1.034	1.045	-0.011
18	0.896	0.0897	-0.001

Table 2: *S*-MTPA ester and *R*-MTPA ester chemical shifts analysis for Aethiopinolone C (**3**)

	S	R	S-R
4β<br>4α	1.926<br>2.711	1.846<br>2.669	+0.080<br>+0.042
5	3.528	3.506	+0.022
3	5.290	5.287	+0.003
2β<br>2α	1.556<br>1.983	1.685<br>2.049	-0.129<br>-0.066
1α	2.397	2.418	-0.021
19	1.019	1.027	-0.008
18	0.948	0.950	-0.002

Table 3: *S*-MTPA ester and *R*-MTPA ester chemical shifts analysis for Aethiopinolone D (**4**)

	S	R	S-R
4β<br>4α	1.999<br>2.530	2.011<br>2.640	-0.012<br>-0.11
5	3.619	3.715	-0.096
3	5.639	5.640	-0.001
2β<br>2α	2.078<br>1.741	1.976<br>1.713	+0.102<br>+0.028
1α<br>1 β	2.657<br>1.361	2.507<br>1.298	+0.15<br>+0.063
19	1.067	1.062	+0.005
18	0.971	0.959	+0.012

ITS sequences of the producing organism

TTGAGGCAAGGGTCAAAAATGGTTTAAGGTAACAGAGTACCTGTCTGACACATAGGCAGACT
ATTGGAAGCAGACAGTCTAAGTAAGCACTGGTGAATATAGATAGAAAACTATTACACCAAAC
AATGCGAACTACAGTCCAGCTAATGCATTTGAGAGGAGCCGATACAGACAGTACCAGCATAC
ATATTGCCTCCAAGTCCAAGCCCCTTCTTCAATTAAGAAAAAGAGGATTGAGAATTACATGAC
ACTCAAACAGGCATGCCCCTCGGAATACCAAGGGGCGCAAGGTGCGTTCAAAGATTCGATGA
TTCACTGAATTCTGCAATTCACATTACTTATCGCATTTCGCTGCGTTCTTCATCGATGCGAGAG
CCAAGAGATCCGTTGTTGAAAGTTGTATATTTGTATTTCGCTCACAGGAGCATTACACATTCAC
AGGAACAAGAAAATGTTTGTATAGGTAAAAGTCAAAGTGTTCATAGTAAGTAAAGCCAAGAT
CATTACTACTGCCAGAAGGGGTACCC

Pictures of the herbarium and the culture of *Fomitiporia aethiopica*

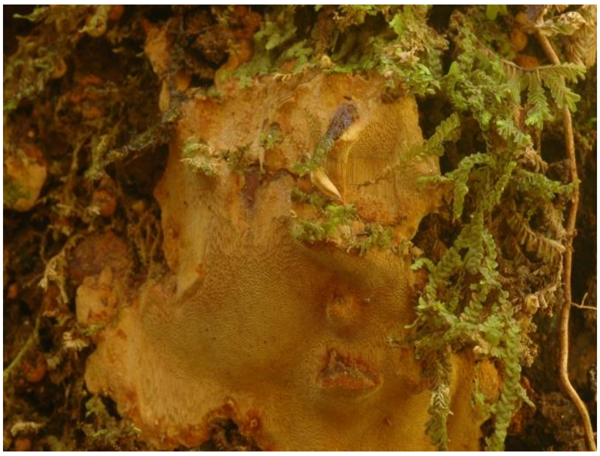

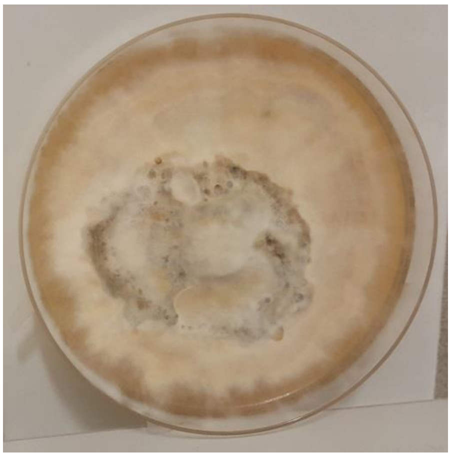

Supplement: Supplementary file 1 [file molecules-23-00369-s001.pdf]
